# KDM4C inhibition blocks tumor growth in basal breast cancer by promoting cathepsin L-mediated histone H3 cleavage

**DOI:** 10.1038/s41588-025-02197-z

**Published:** 2025-06-02

**Authors:** Zheqi Li, Guillermo Peluffo, Laura E. Stevens, Xintao Qiu, Marco Seehawer, Amatullah Tawawalla, Xiao-Yun Huang, Shawn B. Egri, Shaunak Raval, Maeve McFadden, Clive S. D’Santos, Eva Papachristou, Natalie L. Kingston, Jun Nishida, Kyle E. Evans, Ji-Heui Seo, Kendell Clement, Daniel Temko, Muhammad Ekram, Rong Li, Matthew G. Rees, Melissa M. Ronan, Jennifer A. Roth, Anton Simeonov, Stephen C. Kales, Ganesha Rai, Madhu Lal-Nag, David J. Maloney, Ajit Jadhav, Franziska Michor, Alex Meissner, Justin M. Balko, Jason S. Carroll, Matthew L. Freedman, Jacob D. Jaffe, Malvina Papanastasiou, Henry W. Long, Kornelia Polyak

**Affiliations:** 1 Department of Medical Oncology, Dana–Farber Cancer Institute, Boston, MA USA; 2 Department of Medicine, Brigham and Women’s Hospital, Boston, MA USA; 3 Department of Medicine, Harvard Medical School, Boston, MA USA; 4 Center for Functional Cancer Epigenetics, Dana–Farber Cancer Institute, Boston, MA USA; 5 The Eli and Edythe L. Broad Institute, Cambridge, MA USA; 6 Cambridge Research Institute, University of Cambridge, Cambridge, UK; 7 Department of Stem Cell and Regenerative Biology, Harvard University, Cambridge, MA USA; 8 Department of Biostatistics, Harvard T. H. Chan School of Public Health, Boston, MA USA; 9 Department of Data Sciences, Dana–Farber Cancer Institute, Boston, MA USA; 10 National Center for Advancing Translational Sciences, Bethesda, MD USA; 11 Vanderbilt University Medical Center, Nashville, TN USA

**Keywords:** Breast cancer, Epigenetics

## Abstract

Basal breast cancer is a subtype with a poor prognosis in need of more effective therapeutic approaches. Here we describe a unique role for the KDM4C histone lysine demethylase in *KDM4C*-amplified basal breast cancers, where KDM4C inhibition reshapes chromatin and transcriptomic landscapes without substantial alterations of its canonical substrates, trimethylated histone H3 lysine 9 (H3K9me3) and lysine 36 (H3K36me3). Rather, KDM4C loss causes proteolytic cleavage of histone H3 mediated by cathepsin L (CTSL), resulting in decreased glutamate–cysteine ligase expression and increased reactive oxygen species. CTSL is recruited to the chromatin by the grainyhead-like 2 (GRHL2) transcription factor that is methylated at lysine 453 following KDM4C inhibition, triggering CTSL histone clipping activity. Deletion of CTSL rescued KDM4-loss-mediated tumor suppression. Our study reveals a function for KDM4C that connects cellular redox regulation and chromatin remodeling.

## Abstract (web summary)

KDM4C regulates cathepsin L-mediated histone H3 N-terminal tail clipping through grainyhead-like 2 (GRHL2) methylation in breast cancer. This link between the cellular redox state and chromatin remodeling might represent a therapeutic target in *KDM4C*-amplified tumors.

## Main

Breast cancer is a heterogeneous disease classified into luminal, human epidermal growth factor receptor 2-positive (HER2^+^) and basal molecular subtypes^1^. Clinical classification is based on the expression of estrogen (ER), progesterone (PR) and HER2 receptors, distinguishing ER^+^, HER2^+^ and triple-negative (ER–PR–HER2^−^) disease. Triple-negative breast cancer (TNBC) is commonly associated with therapeutic resistance and high risk of distant metastasis, leading to shorter patient survival compared to other subtypes^2^. TNBC is also highly heterogeneous and is further divided into luminal, basal and mesenchymal subtypes with different mutational and therapeutic sensitivity^2–4^. Most basal breast cancers are TNBC, but there is also an *ERBB2*-amplified basal subtype.

Epigenetic regulators are key determinants of cellular states, and thus, epigenetic mutations are a major source of intratumor heterogeneity^5,6^. Post-translational histone modifications shape chromatin states and transcriptomes during normal development and in many diseases, including cancer^7,8^. Frequent somatic mutations in genes encoding histone modifiers like histone demethylases (HDMs) in human cancers underscore their roles in tumorigenesis^9,10^ and highlight them as emerging therapeutic targets in multiple human cancer types.

We and others previously described that *KDM4C*, which encodes a trimethylated histone H3 lysine 9 (H3K9me3) and lysine 36 (H3K36me3) demethylase^11^, is amplified in a subset of TNBC^12,13^. KDM4C has key roles in development and differentiation. In embryonic stem cells (ESCs), it is a target of the OCT4 transcription factor and is required for ESCs self-renewal and the generation of induced pluripotent stem cells^14,15^. The role of KDM4C in tumorigenesis is less well understood, although it is one of the few genes with germline variants associated with a multicancer phenotype^16,17^. In glioblastoma, KDM4C regulates the p53-MYC nexus^18^, while in MLL fusion-driven acute myeloid leukemia, it facilitates epigenetic remodeling by the PRMT1 methyltransferase^19^. In TNBC, KDM4C has been implicated in genomic instability via its effects on chromosome segregation^20,21^. However, the mechanisms by which KDM4C promotes breast tumorigenesis have not been delineated.

Here we report integrated multi-omic characterization of genetic or pharmacological blockade of KDM4C in *KDM4C*-amplified and non-amplified basal breast cancer models. We uncovered an unexpected function for KDM4C as a regulator of cathepsin L (CTSL)-mediated histone H3 N-terminal tail clipping in *KDM4C*-amplified tumors via modulating methylation of the grainyhead-like 2 (GRHL2) transcription factor.

## Results

### *KDM4C* is amplified in breast cancer and drives tumor growth

**Extended Data Fig. 1:**
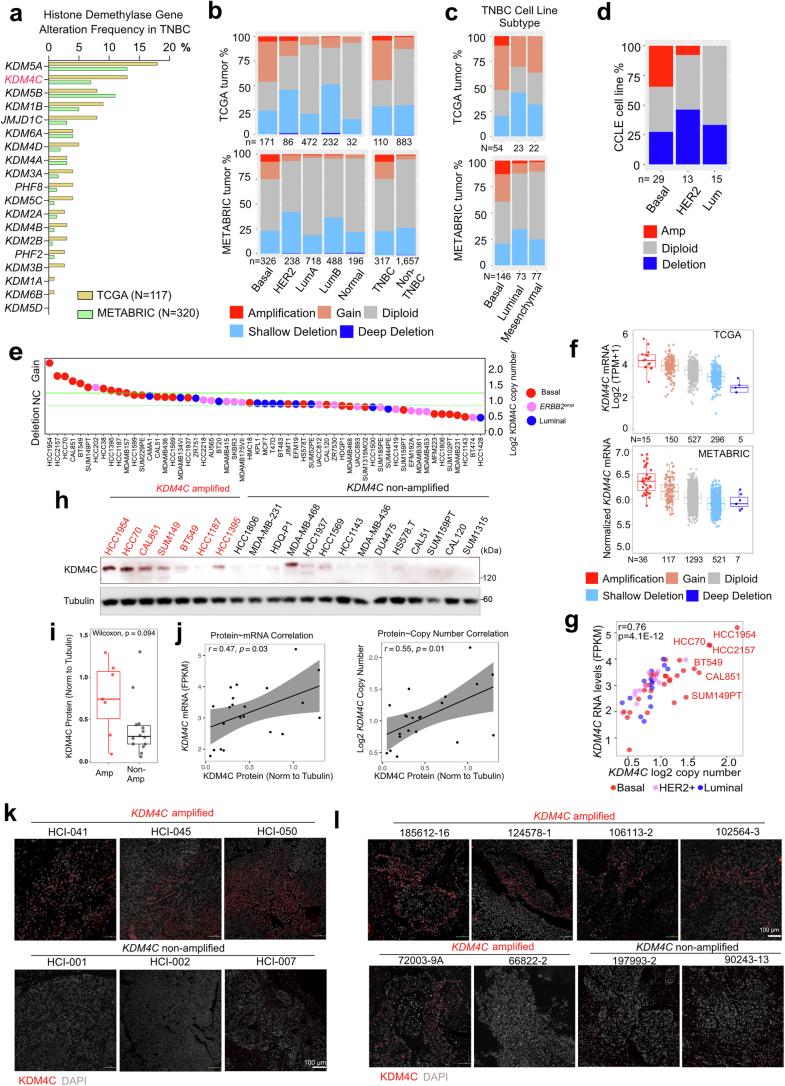
*KDM4C* is frequently amplified in TNBC. **a**, Bar plot representing alteration frequencies of 19 genes encoding histone demethylases in TCGA and METABRIC TNBC tumors. **b**, Stacked bar plots showing *KDM4C* copy number alteration distribution across PAM50 subtypes in TCGA and METABRIC cohorts. **c**, Stacked bar plots showing *KDM4C* copy number alteration distribution across TNBC transcriptomic subtypes. **d**, Stacked bar plot depicting *KDM4C* copy number alteration distribution across 57 breast cancer cell lines. **e**, Dot plot showing log_2_ copy number value of *KDM4C* in 57 breast cancer cell lines from CCLE. Colors indicate tumor subtypes. NC indicates no changes. **f**, Box plots showing *KDM4C* mRNA expression in TCGA and METABIRC TNBC tumors grouped by copy number alteration types. **g**, Scatter plot illustrating correlation of *KDM4C* mRNA expression and log_2_ copy number in 57 breast cancer cell lines. Colors indicate subtype. R and p values were determined by two-sided Pearson correlation. **h**, Immunoblot showing KDM4C protein levels across 21 basal breast cancer cell lines with or without *KDM4C* amplification. Tubulin was used as loading control. **i**, Box plot showing quantification of KDM4C protein levels normalized to tubulin from **h**. Each dot represents a cell line in **h** from n = 7 *KDM4C*-amplified and n = 14 *KDM4C* non-amplified basal breast cancer cell lines. Two-sided Mann–Whitney U test was used. **j**, Scatter plots depicting the correlation of KDM4C protein-to-mRNA and protein-to-copy number across all 21 cell lines in **h**. P and R values were calculated based on two-sided Pearson correlation. The linear regression lines with 95% confidence interval are shown. **k**,**l**, Representative images of immunofluorescence analysis of KDM4C expression in six PDX models (**k**) and eight primary TNBC clinical samples (**l**). Scale bar, 100 μm. *KDM4C* amplification status is indicated for each sample. Staining experiments for **k** and **l** have been performed once, while multiple regions were taken for each tissue. All box plots span the upper quartile (upper limit), median (center) and lower quartile (lower limit). Whiskers extend a maximum of 1.5× IQR. Specific sample sizes are labeled for **b**–**d** and **f**. Source data

**Extended Data Fig. 2:**
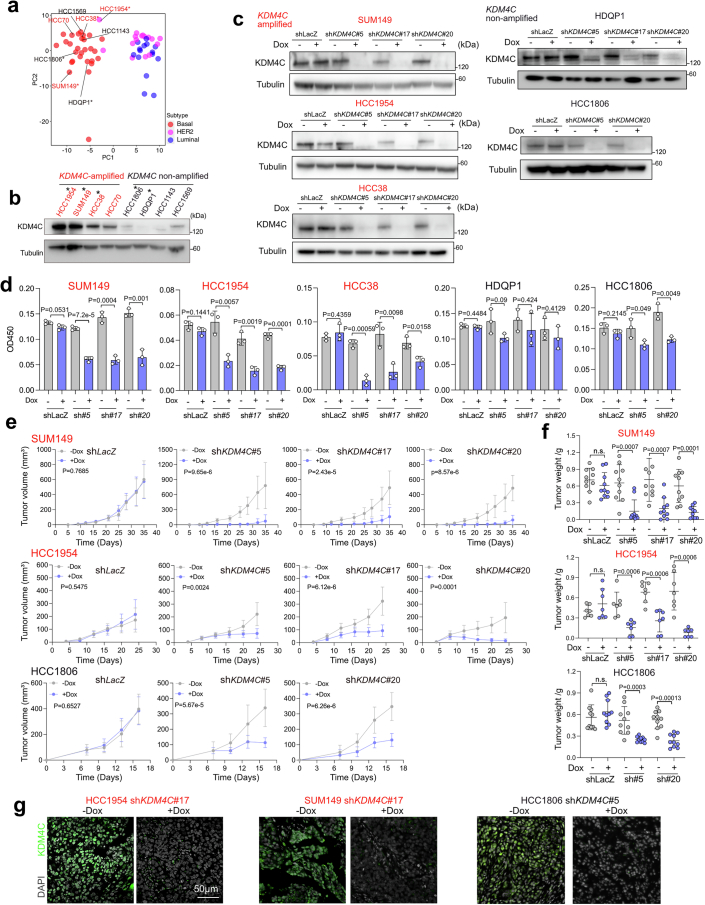
*KDM4C* knockdown diminishes basal breast tumor growth. **a**, Principal component analysis plot showing the clustering of 59 breast cancer cell lines based on PAM50 gene expression panel profiled by CCLE. The names of the eight cell lines used in this study are indicated, and five of these, used for doxycycline-inducible sh*KDM4C* model engineering, are highlighted with an asterisk. **b**, Immunoblot analysis for KDM4C in four *KDM4C*-amplified and four non-amplified cell lines used in this study. Tubulin was used as a loading control. This experiment has been performed once. **c**, Immunoblot showing KDM4C protein levels in the doxycycline-inducible *KDM4C* knockdown models; 2–3 hairpins were used in each line. Tubulin was used as loading control. Longer exposure for HCC1806 and HDQP1 models was used, given their low baseline levels. All experiments were repeated twice with similar results, except HDQP1, which was done once. **d**, Bar plots depicting quantification of colony growth assays in the indicated models in the presence or absence of 1 μg/ml doxycycline. Data represent mean ± s.d. from n = 3 of a representative experiment from at least two independent trials with similar results. Two-sided ordinary one-way ANOVA was used in each cell line. **e**, Growth curves depicting tumor volumes of HCC1954, SUM149 and HCC1806 xenografts expressing sh*LacZ* or sh*KDM4C* Dox-inducible shRNA on control or doxycycline diet. Each dot represents mean ± s.d. of ten tumors per group. Two-sided repeated-measure two-way ANOVA was used for comparing tumor growth ratios within each model. **f**, Dot plots depicting tumor weights at endpoint. Data represent mean ± s.d. of seven (HCC1954) or ten (SUM149 and HCC1806) tumors per group. Two-sided Kruskal–Wallis test was used. **g**, Representative images of KDM4C immunofluorescence in HCC1954, SUM149 and HCC1806 xenografts in the presence or absence of doxycycline diet treatment. DAPI was used to stain nuclei. Scale bar, 50 μm. Source data

*KDM4C* is one of the most frequently mutated genes encoding HDMs in TNBC in The Cancer Genome Atlas (TCGA)^22^ and Molecular Taxonomy of Breast Cancer International Consortium (METABRIC)^23^ cohorts (Extended Data Fig. 1a). Within TNBC, *KDM4C* amplification is associated with the basal TNBC subtype, and *KDM4C*-amplified cell lines are also more commonly basal or triple-negative (Extended Data Fig. 1b–e). *KDM4C* amplification correlates with mRNA and protein levels in patient-derived xenograft (PDX) models, primary TNBC tumors and cell lines (Extended Data Fig. 1f–l). Based on these analyses, we selected four *KDM4C*-amplified basal breast cancer cell lines (SUM149, HCC1954, HCC38 and HCC70) and four *KDM4C*-non-amplified lines (HCC1806, HDQP1, HCC1143 and HCC1569) to assess the functional relevance of KDM4C in basal breast cancer (Extended Data Fig. 2a,b).

**Extended Data Fig. 3:**
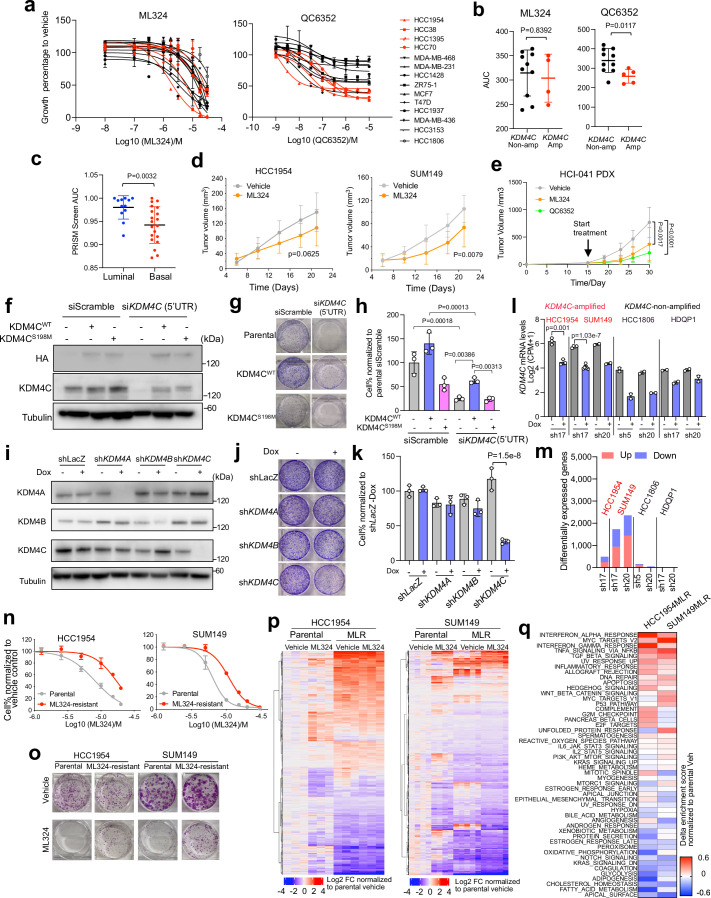
Pharmacological and transcriptomic characterization of KDM4C blockade. **a**, Dose–response curve to ML324 and QC6352 in *KDM4C*-amplified (n = 5 for QC6532 and n = 4 for ML324) and non-amplified (n = 10) breast cancer cell lines (mean ± s.d. with n = 6 from one experiment). **b**, Quantification of area under the curve (AUC) in **a** (two-sided Mann–Whitney U test). **c**, Dot plots showing PRISM screen-derived QC6352 AUC between luminal and (n = 12) and basal (n = 20) breast cancer cell lines (two-sided Mann–Whitney U test). **d**,**e**, Plots depicting tumor volume of HCC1954 and SUM149 xenografts (**d**) and HCI-041 PDX (**e**). Mean ± s.d. from n = 10 (**d**) or n = 4–5 (**e**) tumors per group are shown (two-sided repeated-measure two-way ANOVA) **f**,**i**, Immunoblot for KDM4C and HA in SUM149 cells overexpressing HA-tagged KDM4C^WT^ and KDM4C^S198M^ with siRNA interference against *KDM4C* 5′UTR region for 3 days (**f**) and KDM4A/KDM4B/KDM4C from SUM149 Dox-inducible cell models with or without 1 μg/ml doxycycline for 3 days (**i**) with tubulin was loading controls. (three (**f**) and two (**i**) independent repeats with similar results). **g**,**h**, Representative images (**g**) and quantification (**h**) of colony growth assay with mean ± s.d. from n = 3 of SUM149 parental and KDM4C overexpression models transfected with *KDM4C* 5′UTR siRNA. (two-sided ordinary one-way ANOVA and two independent repeats with similar results). **j**,**k**, Representative images (**j**) and quantification (**k**) of colony growth assay of SUM149-inducible cell models from one experiment (mean ± s.d. with n = 3 and two-sided ordinary one-way ANOVA). **l**, Bar plot showing *KDM4C* mRNA levels from RNA-seq with mean ± s.d. from n = 3 (HCC1954), n = 4 (SUM149 sh17 − Dox), n = 6 (SUM149 sh17 + Dox) and n = 2 (others; two-sided Student’s t test for HCC1954 and SUM149 sh17 models). **m**, Stacked bar plot showing numbers of differentially expressed genes (DEGs). **n**, ML324 dose–response curves of parental and ML324-resistant (MLR) cells (mean ± s.d. with n = 3) **o**, Representative images of colony growth assay in parental and MLR cells treated with or without 10 μm ML324 for 2 weeks. **p**, Heatmaps showing the union of DEGs induced by ML324 or in MLR compared to parental cells normalized to the parental-vehicle controls. **q**, Heatmap depicting the alteration of the Hallmark signature enrichment scores in MLR models. Source data

We first downregulated *KDM4C* using lentiviral doxycycline (Dox)-inducible shRNAs in five cell lines (three amplified versus two non-amplified) and confirmed effective knockdown by immunoblot analysis (Extended Data Fig. 2c). KDM4C downregulation substantially decreased tumor cell growth in vitro and in vivo in all *KDM4C*-amplified cell lines and in a non-amplified cell line (HCC1806) with moderate KDM4C expression (Extended Data Fig. 2c–g). We found that sh*KDM4C* expression had no measurable effects in HDQP1 cells, likely due to negligible endogenous KDM4C levels (Extended Data Fig. 2b). Similarly, treatment with small-molecule inhibitors of the KDM4 family of enzymes, ML324 (ref. ^24^) and QC6352 (ref. ^25^), substantially decreased cell viability in a large panel of cell lines, with QC6352 showing greater selectivity and potency (Extended Data Fig. 3a,b). A PRISM compound screen^26^, including 32 breast cancer cell lines, demonstrated higher sensitivity to QC6352 in basal than in luminal lines (Extended Data Fig. 3c and Supplementary Table 1). ML324 also substantially decreased SUM149 xenograft growth, with a similar trend observed in HCC1954 xenografts (Extended Data Fig. 3d). Both ML324 and QC6352 impaired the growth of a *KDM4C*-amplified TNBC PDX (HCI-041)^27,28^ (Extended Data Fig. 3e).

KDM4C-loss-induced growth inhibition was dependent on its HDM activity because it was rescued by exogenous expression of wild type (WT) but not a catalytically inactive-mutant (S198M) KDM4C in SUM149 cells^29,30^ (Extended Data Fig. 3f–h). Downregulation of two other KDM4 family members, *KDM4A* or *KDM4B*, did not affect the growth of the cell line tested (Extended Data Fig. 3i–k), confirming the specific requirement for KDM4C in basal breast cancer.

Overall, our data in clinical samples and experimental models suggest an oncogenic role for KDM4C in a subset of basal breast tumors.

### KDM4C-blockade-induced transcriptomic and chromatin changes

**Fig. 1:**
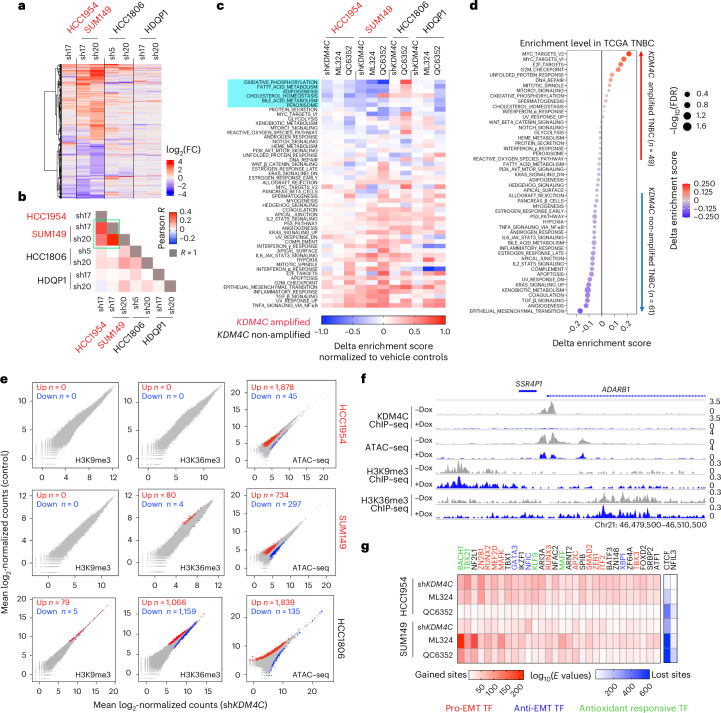
KDM4C inhibition-induced transcriptomic and chromatin remodeling. **a**, Heatmap showing the log_2_(fold change (FC)) of the union of all DEGs between vehicle versus sh*KDM4C* merged from all the indicated Dox-inducible sh*KDM4C* cell models. Gene expression FCs were normalized to each hairpin control. **b**, Heatmap illustrating the Pearson correlation *R* value of log_2_(FCs) of all DEGs from each pairwise comparison. Correlations among *KDM4C*-amplified basal cell lines are highlighted in a green rectangle. **c**, Heatmap depicting alterations of the 50 Hallmark gene signature enrichment scores induced by downregulation of *KDM4C* and KDM4 inhibitor treatments. Delta enrichment scores were calculated by subtracting the scores of control groups from each treatment condition. Pathways were ranked from the most decreased to the most increased upon KDM4C inhibition. Metabolic pathways commonly repressed in *KDM4C*-amplified lines are highlighted by light blue rectangle. **d**, Dot plot showing the 50 Hallmark gene signature enrichment score differences between TNBCs with (*n* = 49) or without (*n* = 61) *KDM4C* copy number gain in the TCGA cohort. Delta enrichment scores were calculated by subtracting the mean values of *KDM4C*-non-amplified group from *KDM4C*-amplified group. **e**, Scatter plots representing the log_2_-normalized counts of H3K9me3 and H3K36me3 ChIP–seq (5 kb bin) and merged ATAC–seq peaks between control and sh*KDM4C* groups in all 3 cell lines. Numbers of differential regions and directionality (up or down) are indicated on each plot. **f**, Genomic track view of KDM4C, ATAC–seq, H3K36me3 and H3K9me3 signals at the *ADARB1* gene locus in SUM149 cells with or without KDM4C knockdown. Chr21, chromosome 21. **g**, Heatmap showing the top 30 and 2 consistently and uniquely enriched motifs in gained and lost ATAC sites, respectively, normalized to vehicle groups in the indicated cell lines. log_10_(*E* values) represent the significance of enrichment. Motifs of transcription factors associated with EMT (pro-EMT or anti-EMT) or antioxidant response are highlighted with different colors. TF, transcriptional factor. Source data

To investigate mechanisms underlying KDM4C-loss-mediated growth inhibition, we first performed RNA sequencing (RNA-seq) in four Dox-inducible sh*KDM4C*-expressing basal breast cancer cell lines, including *KDM4C*-amplified (HCC1954 and SUM149) and *KDM4C*-non-amplified (HCC1806 and HDQP1) cells. Analysis of the RNA-seq confirmed efficient downregulation of *KDM4C* (Extended Data Fig. 3l and Supplementary Table 2), and more substantial and tightly correlated transcriptional changes were found in *KDM4C*-amplified compared to non-amplified cell lines (Fig. 1a,b, Extended Data Fig. 3m and Supplementary Table 2). Functional annotation by gene set variation analysis (GSVA) with Hallmark signature collections revealed that KDM4C genetic depletion or pharmacological inhibition resulted in consistent repression of multiple major metabolic pathways (for example, cholesterol homeostasis and oxidative phosphorylation) uniquely in *KDM4C*-amplified basal cell lines except for QC6352 treatment in HDQP1 cells (Fig. 1c). In line with this observation, cholesterol homeostasis and oxidative phosphorylation were among the top differentially enriched pathways between *KDM4C*-amplified and *KDM4C*-non-amplified TNBCs in the TCGA cohort together with key regulators of cell proliferation (for example, MYC targets; Fig. 1d). We also observed activated transforming growth factor β (TGF-β) signaling and epithelial-to-mesenchymal transition (EMT) in all four cell lines tested, indicating a shift in cell states.

We also generated ML324-resistant derivatives of HCC1954 and SUM149 cell lines (HCC1954-MLR and SUM149-MLR) by prolonged culture with ML324 (10 μm) to help delineate mechanisms of response and acquired resistance to KDM4 inhibitors (Extended Data Fig. 3n,o). RNA-seq of MLR lines revealed transcriptional patterns similar to those of ML324-treated parental cells but also identified resistance-specific changes (Extended Data Fig. 3p). Only a few pathways were commonly altered between the two MLR lines, but TGF-β signaling and cholesterol homeostasis were among the top upregulated and downregulated pathways, respectively, highlighting their importance in KDM4C-driven tumors (Extended Data Fig. 3q).

**Extended Data Fig. 4:**
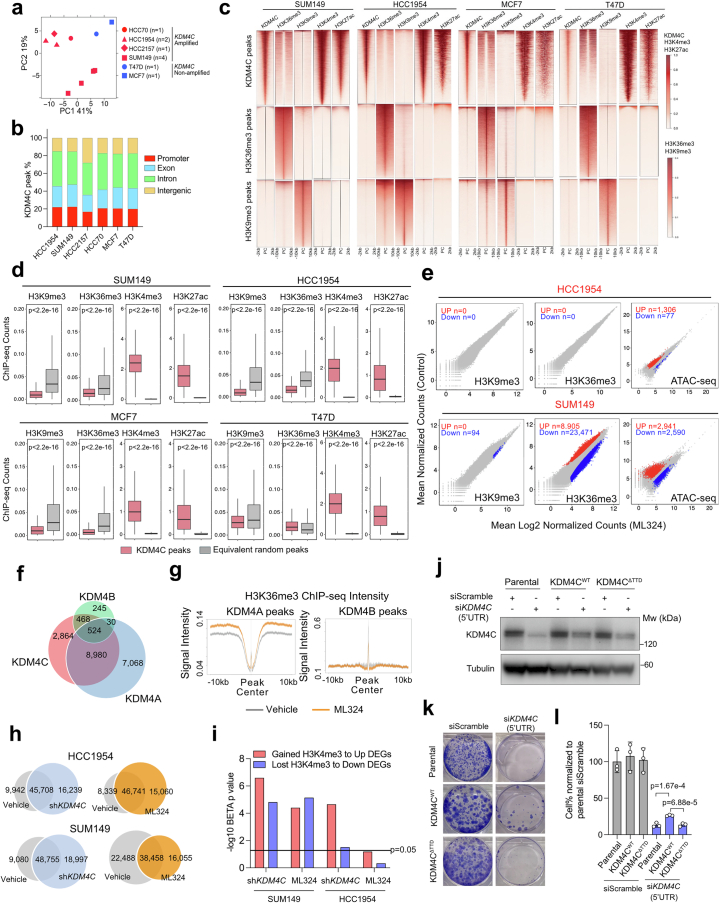
Chromatin alterations following KDM4C inhibition. **a**, Principal component analysis of KDM4C peaks in the indicated cell lines. **b**, Stacked bar plots showing the distribution of KDM4C peaks in the indicated cell lines in distinct genomic regions. **c**, Heatmap illustrating ChIP–seq peaks for the indicated marks and cell lines. Windows of peak center with ±2 kb or ±10 kb are shown. **d**, Box plots showing the ChIP–seq read counts for the indicated histone mark projected on KDM4C peaks or the equivalent number of random peaks (HCC1954, n = 21,768; SUM149, n = 19,675; MCF7, n = 18,160; T47D, n = 19,244). Box plots span the upper quartile (upper limit), median (center) and lower quartile (lower limit). Whiskers extend a maximum of 1.5× IQR (two-sided Mann–Whitney U test). **e**, Scatter plots representing the log_2_-normalized counts of H3K9me3 and H3K36me3 ChIP–seq (5 kb bin) and merged ATAC–seq peaks between controls and ML324-treated groups in two cell lines. Numbers of differential regions are indicated. **f**, Venn diagram showing the intersection of KDM4A, KDM4B and KDM4C ChIP–seq peaks in the SUM149 cell line. **g**, Plot depicting H3K36me3 ChIP–seq signal in the presence or absence of ML324 treatment at KDM4A or KDM4B peaks. Windows of peak center with ±10 kb are shown. The 95% confidence interval of each curve is presented. **h**, Venn diagrams showing intersections of H3K4me3 peaks between vehicle and sh*KDM4C* or ML324-treated groups in two cell lines. **i**, Bar plots showing −log_10_(p) calculated by the BETA algorithm representing the association of differential H3K4me3 peaks with differentially expressed genes from RNA-seq in the indicated contexts. **j**, Immunoblot analysis for KDM4C in cell lysates of SUM149 cells with or without KDM4C^WT^ or KDM4C^ΔTTD^ overexpressing with 5′UTR region siRNA interference for 3 days. Tubulin was used as loading control. This experiment was repeated independently three times with similar results. **k**,**l**, Representative images (**k**) and quantification (**l**) of colony growth assay of SUM149 parental and KDM4C^WT^ or KDM4C^ΔTTD^ mutant overexpression models transfected with siRNA against *KDM4C* 5′UTR region. Data are presented as mean ± s.d. with n = 3 normalized to parental with siScramble group from a representative experiment from three independent repeats with similar results (two-sided ordinary one-way ANOVA). Source data

Next, we performed chromatin immunoprecipitation followed by sequencing (ChIP–seq) for KDM4C in four *KDM4C*-amplified basal (HCC1954, SUM149, HCC70 and HCC2157) and two non-amplified ER^+^ luminal (T47D and MCF7) cell lines and found strong cell-type specificity of KDM4C chromatin peaks but similar distributions at promoter and non-promoter loci (Extended Data Fig. 4a,b). Integrating KDM4C binding and histone modification patterns demonstrated mutual exclusivity of KDM4C with its substrates H3K9me3 and H3K36me3 in most cell lines, except H3K36me3 in T47D, and substantial co-occurrence with H3K4me3 and H3K27ac peaks in all four lines (Extended Data Fig. 4c,d). We also assessed global differences in H3K9me3 and H3K36me3 signal intensity after KDM4C blockade, but neither sh*KDM4C* nor ML324 treatment caused substantial changes in the two *KDM4C*-amplified basal cell lines except for H3K36me3 in ML324-treated SUM149 cells (Fig. 1e and Extended Data Fig. 4e). In contrast, pronounced differences in H3K9me3 and H3K36me3 were observed following KDM4C downregulation in HCC1806 cells, a *KDM4C*-non-amplified cell line with moderate KDM4C expression (Fig. 1e). The differences between ML324 treatment and shRNA knockdown could potentially be explained by the inhibition of other KDM4 family members by ML324. Thus, we also performed ChIP–seq for KDM4A and KDM4B and found distinct overlap in genomic binding sites among all three KDM4 enzymes, and KDM4A-binding sites were associated with the elevated H3K36me3 levels after ML324 treatment (Extended Data Fig. 4f,g).

Assay for transposase-accessible chromatin using sequencing (ATAC–seq) showed extensive remodeling of accessible chromatin after KDM4C suppression in all three cell lines, albeit to a variable degree. There was a predominant gain in open chromatin in HCC1954 and HCC1806 cells, while SUM149 cells showed a less pronounced increase (Fig. 1e). Chromatin changes in *KDM4C*-amplified cells were not associated with changes in H3K9me3 or H3K36me3 signal intensity, as exemplified by the *ADARB1* genomic locus (Fig. 1f). Moreover, transcription factor motifs enriched in top gained ATAC sites were related to EMT (for example, ZEB1 and SMAD2) and antioxidant pathways (for example, BACH1 and NF2L1), while CTCF and NFIL3 were the only motifs enriched in lost ATAC sites (Fig. 1g).

We also analyzed changes in H3K4me3 signal following KDM4C inhibition due to the prominent overlap detected between KDM4C and H3K4me3 peaks (Extended Data Fig. 4c,d), which has also been reported in ESCs^31^. Downregulation or inhibition of KDM4C led to substantial gains (22.1% in HCC1954 and 22.8% in SUM149) and losses (12.9% in HCC1954 and 27% in SUM149) in H3K4me3 peaks, although most peaks were still detected in all conditions (Extended Data Fig. 4h). Alterations in H3K4me3 peaks were substantially associated with transcriptomic changes except in ML324-treated HCC1954 cells (Extended Data Fig. 4i). A prior study showed that KDM4C is mainly recruited to H3K4me3 marks via its tandem tudor domain (TTD)^32^. To test if the TTD domain is required for KDM4C knockdown-induced cell growth suppression, we downregulated endogenous KDM4C and exogenously expressed WT KDM4C or a mutant lacking the TTD domain (ΔTTD) in SUM149 cells (Extended Data Fig. 4j). We found that only the WT KDM4C was able to partially rescue the growth inhibitory phenotype (Extended Data Fig. 4k,l), suggesting that the KDM4C–H3K4me3 interaction may be required for this function.

Overall, these data showed that in *KDM4C*-amplified lines, KDM4C blockade induced substantial changes in chromatin patterns with limited changes in its canonical substrates H3K9me3 and H3K36me3.

### KDM4C inhibition induces proteolytic cleavage of histone H3

**Fig. 2:**
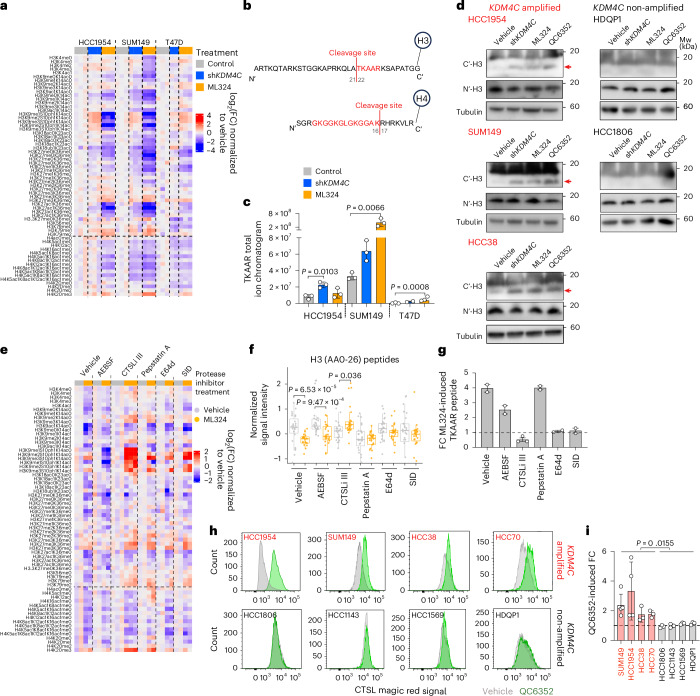
KDM4C inhibition induces proteolytic cleavage of histone tails. **a**, Heatmap showing histone peptide abundance by MS in the indicated cell lines expressing Dox-inducible sh*KDM4C* following control (no Dox, dimethyl sulfoxide (DMSO)), sh*KDM4C* induction (1 μg ml^−1^ Dox, DMSO) or 10 μm ML324 (no Dox) treatment. Peptide abundances were normalized to the mean values of vehicle group within each cell line and ranked from N to C terminus. **b**, Schematic illustration of proteolytic cleavage sites in histones H3 and H4 after *KDM4C* blockade. **c**, Bar plot showing the clipped H3 peptide (TKAAR) total ion chromatogram signal intensity in the indicated groups. Mean ± s.d. are shown. Two-sided Dunnett’s test was used within each cell line for groups with biological triplicates, except T47D sh*KDM4C* group. **d**, Immunoblot for histone H3 using C (C′-H3) and N (N′-H3) terminal antibodies in 5 cell lines with inducible sh*KDM4C* infection, following control (DMSO, no Dox), 1 μg ml^−1^ Dox (sh*KDM4C*), 10 μm ML324 (no Dox) or 1 μm QC6352 (no Dox) for 5 days. Tubulin was used as a loading control. Clipped H3 bands are marked with red arrow. Experiments were repeated independently three times (HCC1954, SUM149 and HCC38) or twice (HDQP1 and HCC1806) with similar results. **e**, Heatmap showing histone peptide abundance by MS in SUM149 cell line following DMSO (vehicle) or 10 μm ML324 treatment in the presence or absence of the indicated protease inhibitors (100 μm AEBSF HCl, 100 μm pepstatin A, 10 μm SID2668150, 10 μm E64d and 5 μm CTSLi-III) for 24 h. Peptide abundances were normalized to the mean values of vehicle group within each cell line and ranked from N to C terminus. **f**, Box plots depicting differences in N-terminal histone H3 (amino acid positions 0–26) peptide abundances between vehicle and KDM4C-inhibited samples following the indicated protease treatment in the SUM149 cell line. Box plots span the upper quartile (upper limit), median (center) and lower quartile (lower limit). Whiskers extend a maximum of 1.5× IQR. Statistical significance of differences was determined by two-sided Kruskal–Wallis test. **g**, Bar plot showing the ML324-induced FC of clipped H3 peptide (TKAAR) total ion chromatogram signal intensity in the indicated groups. Mean ± s.d. are shown for each group with *n* = 3 (CTSLi-III group) and *n* = 2 (all the rest) biological replicates. **h**, Representative flow cytometry plots depicting the shift of CTSL magic red signal after 1 μm QC6352 treatment for 5 days. **i**, Bar plot summarizing the QC6352-induced FCs in CTSL activity merging from *n* = 5 (SUM149), *n* = 4 (HCC1954) and *n* = 3 (other cell lines) independent experiments in each cell line (mean ± s.d.). Two-sided Mann–Whitney *U* test was used to compare average FCs between four *KDM4C*-amplified and four non-amplified cell lines. SID, SID2668150; AEBSF, 4-(2-aminoethyl)benzenesulfonyl fluoride hydrochloride. Source data

**Extended Data Fig. 5:**
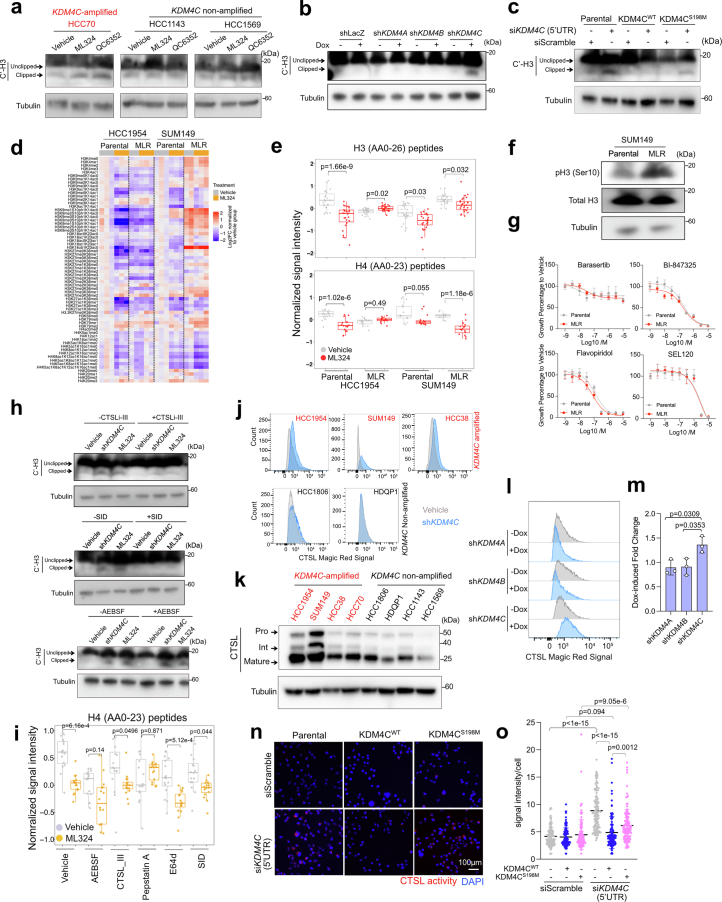
KDM4C inhibition induces proteolytic cleavage of histone tails. **a**–**c**, Immunoblot for C′-H3 in three cell lines following DMSO, 10 μm ML324 or 1 μm QC6352 treatment for 5 days (**a**), SUM149 doxycycline-inducible models with or without 1 μg/ml doxycycline for 5 days (**b**) and SUM149 cells overexpressing KDM4C^WT^ and KDM4C^S198M^ with siRNA against *KDM4C* 5′UTR for 5 days (**c**; two independent repeats for all with similar results). **d**, Heatmap showing normalized histone peptide abundance in parental and MLR models with or without ML324 treatment. **e**,**i**, Box plots showing average N-terminal peptides abundances of histone H3 (n = 27 peptides) or H4 (n = 16 peptides) in the indicated groups (two-sided Kruskal–Wallis test). Box plots span the upper quartile (upper limit), median (center) and lower quartile (lower limit). Whiskers extend a maximum of 1.5× IQR. **f**, Immunoblot for total and phospho-histone H3 (Ser10) in SUM149 parental and MLR cells (two independent repeats with similar results). **g**, Cell viability curves normalized to vehicle group in response to CDK8 or AURKA/AURKB inhibitors in SUM149 parental and MLR cells (mean ± s.d. of n = 6 from one experiment). **h**, Immunoblot for C′-H3 in SUM149-inducible-sh*KDM4C* cells with either 1 μg/ml doxycycline or 10 μm ML324 with different protease inhibitors (5 μm CTSL-inhibitor III, 10 μm SID2668150 or 100 μm AEBSF), with tubulin as loading control (three independent repeats for CTSLi-III experiment with similar results and one experiment for the rest). **j**, Representative plots depicting the CTSL activity signal in the indicated sh*KDM4C* cell models with 1 μg/ml doxycycline treatment for 5 days. **k**, Immunoblot analysis for CTSL in the indicated cell lines from one experiment. **l**,**m** Representative plot (**l**) and quantification of mean ± s.d. from three independent experiments (**m**) of indicated SUM149 models with or without 1 μg/ml doxycycline for 5 days (two-sided ordinary one-way ANOVA). **n**,**o**, Representative images of CTSL activity signal in SUM149 cells overexpressing KDM4C^WT^ and KDM4C^S198M^ with siRNA interference against *KDM4C* 5′UTR for 5 days (**n**) and quantification of signals of 120 cells from three to four representative regions in mean ± s.d. (two-sided ordinary one-way ANOVA; **o**). Scale bar, 100 μm. Tubulin was used as loading control for all the immunoblots. Source data

Due to the discrepancy between extensive global accessible chromatin remodeling and limited H3K9me3 and H3K36me3 alterations following KDM4C inhibition, we investigated global changes in histone modification patterns in a comprehensive and unbiased manner using histone mass spectrometry (MS). Surprisingly, we detected the loss of nearly all histone marks corresponding to the N-terminal parts of histones H3 and H4 following ML324 treatment in SUM149 and HCC1954 cells, with *KDM4C* knockdown also exhibiting a similar but weaker trend (Fig. 2a). Generally, a decrease in the levels of some histone marks is compensated by an increase in others. Thus, the concomitant loss of most N-terminal marks could potentially be explained by a proteolytic cleavage event that removed the N terminus of these histones. To investigate this hypothesis, we performed an unbiased proteomic analysis of the extracted histones and found peptides consistent with non-canonical cleavage events at H3A21 and H4K16 (Fig. 2b). We quantified the non-canonical TKAAR peptide from H3 and discerned a general induction of clipped H3 peptides following KDM4C blockade in HCC1954 and SUM149 cells with limited changes in T47D cells (Fig. 2c). Immunoblot analysis using a C-terminal H3 antibody confirmed the increase in N-terminal tail clipping after *KDM4C* knockdown or blockade specifically in the four *KDM4C*-amplified cell lines, while the N-terminal H3 antibody did not detect noticeable differences, likely due to the rapid degradation of the cleaved peptide (Fig. 2d and Extended Data Fig. 5a). Downregulation of KDM4A or KDM4B did not induce histone tail clipping (Extended Data Fig. 5b), and KDM4C-induced clipping was prevented by ectopic overexpression of WT but not catalytically inactive-mutant KDM4C^S198M^ (Extended Data Fig. 5c), indicating that it is specific to KDM4C and requires its demethylase function. Histone MS of ML324-resistant derivatives demonstrated higher baseline H3 clipping in HCC1954-MLR cells that was not further increased by ML324, whereas limited baseline and higher ML324-induced H3 clipping was observed in SUM149-MLR cells (Extended Data Fig. 5d,e). We also noticed a strong increase in H3Ser10 phospho-peptide in SUM149-MLR cells, which was confirmed by immunoblot (Extended Data Fig. 5f). However, SUM149-MLR cells did not show differential sensitivity toward inhibitors of H3Ser10 kinases (AURKA/AURKB and CDK8; Extended Data Fig. 5g), suggesting a lack of functional relevance.

To identify the endopeptidase that mediates KDM4C-loss-induced histone H3 and H4 tail clipping, we repeated the MS analyses in the presence of various protease inhibitors. Quantification of histone H3 total and cleaved peptides, as well as immunoblot validation, revealed that inhibition of CTSL (by CTSLi-III and SID2668150) or cysteine protease inhibitor E64d, but not serine or aspartyl proteases, reduced histone clipping (Fig. 2e–g and Extended Data Fig. 5h), identifying CTSL as the strongest candidate responsible for H3 clipping. In contrast, loss of H4 peptide was partially rescued only by aspartyl protease inhibitor pepstatin A (Extended Data Fig. 5i), suggesting different mechanisms for histone H3 and H4 proteolysis. CTSL has previously been reported to cleave H3 in mouse ESCs at exactly the same amino acid position (H3A21) as we observed^33,34^. Further investigation revealed increased cellular CTSL activity following KDM4C blockade or downregulation only in the *KDM4C*-amplified cell lines (Fig. 2h,i, Extended Data Fig. 5j and Supplementary Fig. 1), strengthening the link between histone H3 clipping and CTSL activity. The increase in CTSL activity after KDM4 inhibition was more pronounced in SUM149 and HCC1954 cells, likely due to higher baseline CTSL expression (Extended Data Fig. 5k), which was specific to KDM4C because it was not observed following knockdown of KDM4A or KDM4B (Extended Data Fig. 5l,m). Ectopic expression of WT but not catalytically inactive KDM4C mutant diminished CTSL activation (Extended Data Fig. 5n,o), indicating the requirement for demethylase function.

**Fig. 3:**
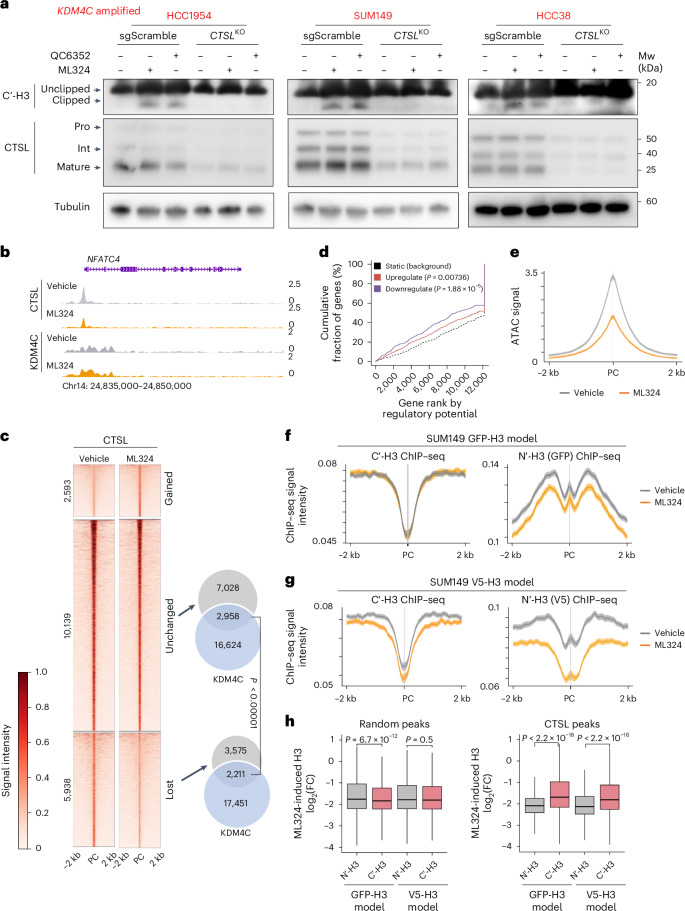
CTSL is a chromatin-bound histone H3 protease activated by KDM4C inhibition. **a**, Immunoblot showing H3 protein detected with C-terminal antibodies and CTSL in three *KDM4C*-amplified cell lines after 5 days of treatments with DMSO, 10 μm ML324 or 1 μm QC6352 in sgScramble and *CTSL* KO models. Tubulin was used as a loading control. All experiments were repeated independently at least twice with similar results. **b**, Genomic track view of KDM4C and CTSL binding signals in SUM149 cell lines with or without ML324 treatment at the *NFATC4* gene locus. Chr14, chromosome 14. **c**, Heatmap showing differential and unchanged CTSL peaks after ML324 treatment in SUM149 cell line. Signal intensity is illustrated in a 4 kb window. Venn diagram on the right side illustrating the intersection of unchanged and lost CTSL peaks with KDM4C binding sites. Fisher’s exact test (two-sided) was used. **d**, Line plot showing Binding and Expression Target Analysis (BETA) to assess the association between lost CTSL sites and DEGs in SUM149 cells following ML324 treatment. Statistical comparison to the background genes was performed using one-sided Kolmogorov–Smirnov test. **e**–**g**, Intensity plots representing ATAC–seq signal at CTSL peaks lost following ML324 treatment in SUM149 cell line (**e**), histone H3 signal using the indicated antibodies for ChIP in vehicle and ML324-treated SUM149 cells expressing N-terminal GFP- (**f**) or V5-tagged (**g**) histone H3 at CTSL binding sites at the range of ±2 kb of the PC. The 95% confidence interval of each curve is presented. **h**, Box plots showing ML324-induced H3 signal changes in each indicated ChIP–seq sample at CTSL peaks or at the same number of random peaks (*n* = 16,141 peaks). Box plots span the upper quartile (upper limit), median (center) and lower quartile (lower limit). Whiskers extend a maximum of 1.5× IQR. Two-sided Mann–Whitney *U* test was used. PC, peak center. Source data

**Extended Data Fig. 6:**
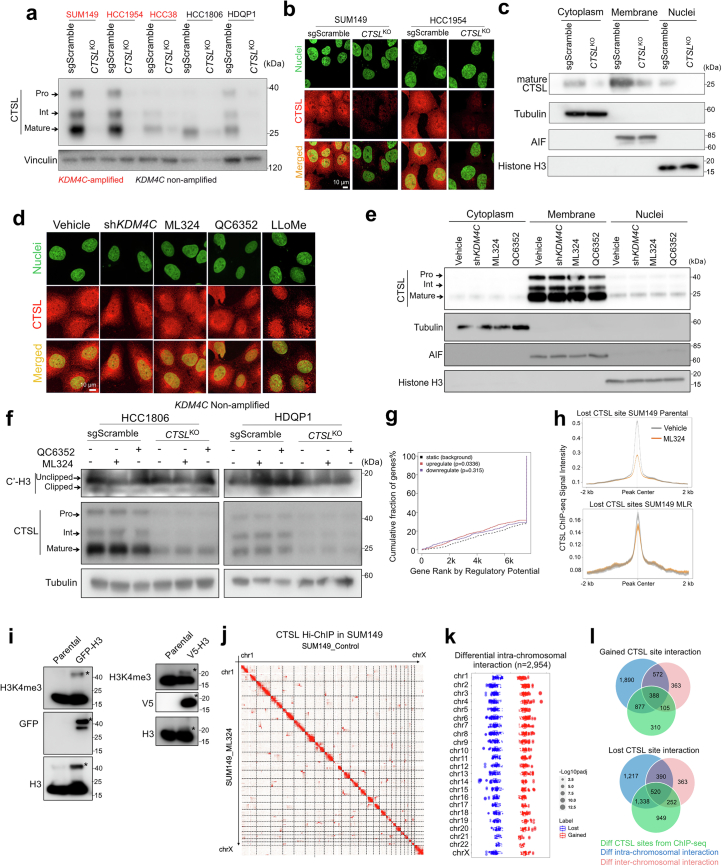
CTSL is a chromatin-bound histone H3 protease activated by KDM4C inhibition. **a**, Immunoblot depicting CTSL in sgScramble and *CTSL*^KO^ models in the indicated cell lines with vinculin as a loading control (two independent repeats with similar results). **b**, Immunofluorescence staining of CTSL and nuclei in the corresponding sgScramble and *CTSL*^KO^ derivatives from one experiment. Scale bar, 10 μm. **c**, Immunoblot depicting CTSL in different fractions in SUM149 sgScramble and *CTSL*^KO^ derivatives with tubulin, AIF and histone H3 as controls for subcellular fractionation (two independent repeats with similar results). **d**, Immunofluorescence for CTSL and nuclei in SUM149-inducible-sh*KDM4C* cells treated with DMSO, 1 μg/ml doxycycline (sh*KDM4C*), 10 μm ML324 or 1 μm QC6352 for 3 days from one experiment. Treatment of 1 μm LLoMe for 24 h was a positive control. Scale bar, 10 μm. **e**, Immunoblot for CTSL in the indicated fractions of SUM149-inducible-sh*KDM4C* cells under the same treatment as **d** for 5 days (three independent repeats with similar results). **f**, Immunoblot showing C′-H3 and CTSL in HCC1806 and HDQP1 sgScramble and *CTSL*^KO^ derivatives with 10 μm ML324 or 1 μm QC6352 treatment for 5 days with tubulin as loading control (two independent repeats with similar results). **g**, Binding and Expression Target Analysis showing association between ML324-induced gained CTSL sites and differentially expressed genes in SUM149 cells (one-sided Kolmogorov–Smirnov test). **h**, Intensity plots depicting CTSL ChIP–seq signal in vehicle and ML324-treated SUM149 parental and MLR cells at lost CTSL sites at ±2 kb range of the peak centers. The 95% confidence interval is presented. **i**, Immunoblot showing H3K4me3, total H3 levels in SUM149 cells ectopically expressing GFP- (left) and V5-tagged (right) histone H3 from one experiment. Ectopic proteins were differentiated by molecular weight and indicated by an asterisk. **j**, Heatmap showing intrachromosomal and interchromosomal CTSL interactions in SUM149 cells with vehicle or ML324 treatment. **k**, Box plot showing ML324-induced differential intrachromosomal CTSL interactions with frequency >10 and adjusted p value < 0.05. Box plots span the upper quartile (upper limit), median (center) and lower quartile (lower limit). Whiskers extend a maximum of 1.5× IQR. **l**, Venn diagrams showing intersections among ML324-induced gained or lost CTSL binding sites in ChIP–seq and intrachromosomal or interchromosomal interaction sites in Hi-ChIP. Source data

To prove a role for CTSL in KDM4C-blockade-induced histone clipping, we deleted *CTSL* in five basal breast cancer cell lines (three *KDM4C*-amplified and two non-amplified) using CRISPR–Cas9 (Extended Data Fig. 6a). CTSL immunofluorescence and immunoblot using fractionated cell lysates demonstrated nuclear localization in parental cell lines, but a lack of nuclear signal in *CTSL* knockout *(CTSL*^KO^) lines (Extended Data Fig. 6b,c). The subcellular localization of CTSL and its maturation were not affected by KDM4C blockade (Extended Data Fig. 6d,e), suggesting that KDM4C-loss-induced histone cleavage is not due to lysosomal rupture-induced translocation of CTSL to the nucleus, as has been reported for external stressors such as viral infection^35^. Immunoblot analyses using antibodies against the C terminus of H3 revealed a decrease in KDM4 inhibition-induced H3 clipping in *CTSL*^KO^ lines in all three *KDM4C*-amplified cell lines (Fig. 3a and Extended Data Fig. 6f).

Overall, these results established a link between KDM4C demethylase activity and chromatin remodeling via CTSL-mediated histone H3 cleavage in *KDM4C*-amplified cell lines.

### Nuclear CTSL is activated by KDM4C inhibition

To confirm the localization of CTSL to the chromatin, we performed CTSL ChIP–seq in SUM149 cells that showed the most pronounced H3 clipping. We detected sharp CTSL peaks resembling transcription factor binding and marked changes after ML324 treatment (Fig. 3b,c). CTSL peaks lost after ML324 treatment (effective CTSL sites) were associated with ML324-induced differentially expressed genes (DEGs) and decreased chromatin accessibility (Fig. 3d,e), whereas gained peaks showed limited association with transcriptional changes (Extended Data Fig. 6g). CTSL peak intensity was not changed in SUM149-MLR cells after ML324 treatment, suggesting that acquired resistance to ML324 is associated with diminished histone H3 tail clipping (Extended Data Fig. 6h). CTSL peaks lost after ML324 treatment substantially overlapped with KDM4C binding sites compared to unchanged peaks (Fig. 3c), highlighting the importance of CTSL loss in ML324-induced chromatin remodeling and the involvement of KDM4C in this process.

To further substantiate N-terminal H3 clipping following KDM4C inhibition and to eliminate the possibility of an unknown N-terminal H3 modification blocking antibody binding, we expressed N-terminal GFP or V5-tagged H3 in SUM149 cells (Extended Data Fig. 6i) and performed ChIP–seq using antibodies against GFP or V5 as well as the C terminus of H3. We detected a more pronounced decrease in overall H3 ChIP–seq signal using GFP or V5 antibodies than C-terminal H3 antibody following ML324 treatment at CTSL binding sites, which was not observed at randomly selected peaks (Fig. 3f–h). The alterations were more pronounced in the V5-tagged than in the GFP-tagged H3 model, potentially due to the steric hindrance associated with the larger GFP tag.

We also performed CTSL Hi-C and ChIP–seq (Hi-ChIP)^36^ in SUM149 cells to assess ML324-induced global changes in chromatin organization mediated by CTSL. We identified 2,954 and 4,992 differential intrachromosomal and interchromosomal interaction regions induced by ML324 (Extended Data Fig. 6j,k), which overlapped with the majority (74%) of differential CTSL ChIP–seq peaks (Extended Data Fig. 6l), implying that these higher chromatin conformational changes were outcomes of CTSL acting in *cis*.

These data establish that KDM4C-inhibition-induced H3 tail clipping occurs at CTSL peaks that are lost after KDM4C inhibition and results in transcriptional reprogramming via changes in chromatin accessibility and conformation.

### GRHL2 mediates CTSL chromatin binding and activity

**Fig. 4:**
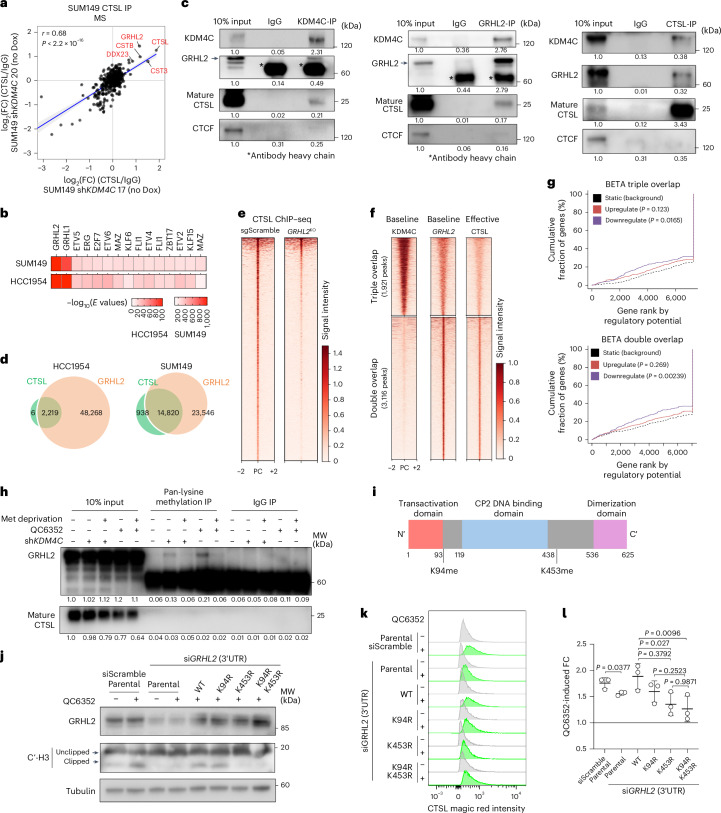
GRHL2 recruits CTSL to the chromatin, and its methylation modulates CTSL activity. **a**, Scatter plot showing the correlation of log_2_(FC) (normalized to IgG control) MS signal of proteins detected in CTSL immunoprecipitants in SUM149 cell models sh*KDM4C* #17 and sh*KDM4C* #20 at baseline without Dox treatment. The linear regression line with 95% confidence interval is shown. *P* values were derived from two-sided Pearson correlation. **b**, Heatmap depicting rank order of transcription factor binding site motifs enriched in CTSL binding sites of vehicle-treated cells in SUM149 and HCC1954 cell lines. log_10_(*E* values) were used to define the significance of enrichment. **c**, Immunoblot analysis of KDM4C, GRHL2 and CTSL immunoprecipitants and 10% of input detected with the indicated antibodies in SUM149 cells. CTCF was used as negative control. Signal intensity normalized to input is indicated for each protein. All experiments were repeated at least twice independently with similar results. **d**, Venn diagrams showing intersections of CTSL and GRHL2 binding sites in HCC1954 and SUM149 cells. **e**, Heatmap showing overall intensities of CTSL chromatin binding in scramble control and *GRHL2* KO SUM149 cell line. Signal intensity is illustrated in a 4 kb window (PC). **f**, Heatmaps illustrating triple (CTSL^+^GRHL2^+^KDM4C^+^) and double (CTSL^+^GRHL2^+^) overlapping peaks in SUM149 cell lines. Signal intensity is depicted in a 4 kb window. **g**, Line plot showing BETA for assessing the association of triple and double overlapping peaks with differentially expressed genes in SUM149 following ML324 treatment. One-sided Kolmogorov–Smirnov test was applied to calculate the *P* values. **h**, Immunoblot for GRHL2 and CTSL in 10% input and immunoprecipitants of pan-lysine methylation and IgG antibody from cells with the indicated treatments. GRHL2 and CTSL signal normalized to input is indicated for each condition. The experiment was repeated three times independently with similar results. **i**, Schematic view of GRHL2 protein structure indicating the location of the lysine methylation sites. **j**, Immunoblot for GHRL2 and C-terminal histone H3 following 3 days of treatment with vehicle or 1 μm QC6352 of SUM149 cells expressing WT or the indicated mutant GRHL2. This experiment was repeated twice independently with similar results. **k**, Representative flow cytometry plots depicting the shift of CTSL activity signal in SUM149 cells with the indicated conditions. **l**, Bar plot summarizing the QC6352-induced CTSL magic red FCs in SUM149 cell models merging three independent experiments (mean ± s.d.). Two-sided ordinary one-way analysis of variance (ANOVA) test was used. Source data

**Extended Data Fig. 7:**
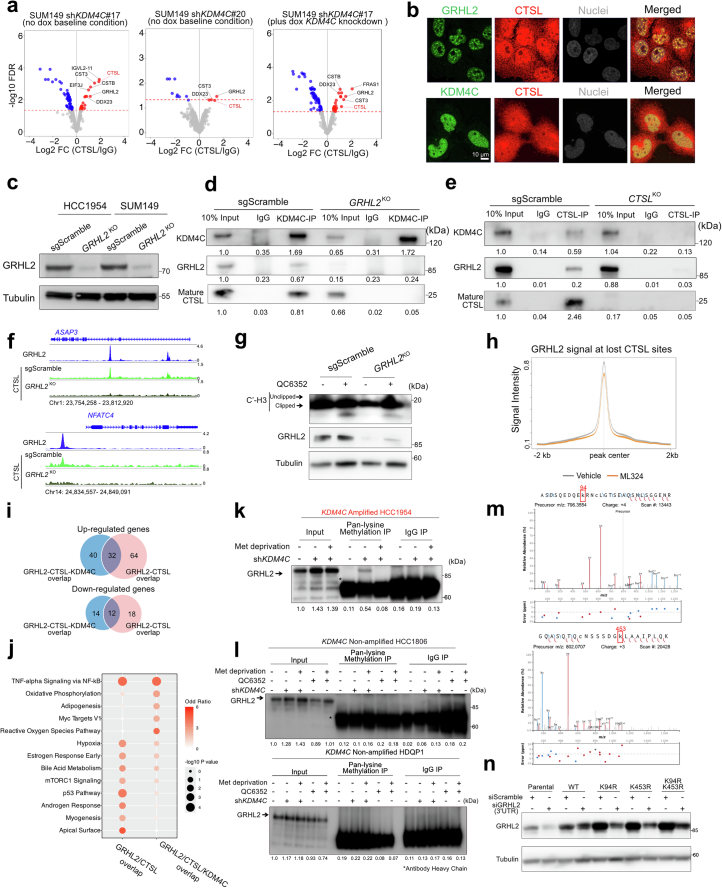
Regulation of CTSL chromatin binding and activity by GRHL2. **a**, Volcano plots showing CTSL-interacting proteins in SUM149 sh*KDM4C* models (17 and 20 without doxycycline and 17 with doxycycline treatment) identified by mass spectrometry of CTSL immunoprecipitants. Red and blue indicate targets with FDR < 0.05 compared to IgG control. **b**, Immunofluorescence staining of CTSL (red), nuclei (gray) and GRHL2 or KDM4C (green) in SUM149 cell line from one experiment. Scale bar, 10 μm. **c**, Immunoblot depicting the expression of GRHL2 in HCC1954 and SUM149 sgScramble and *GRHL2*^KO^ derivatives with tubulin as a loading control (two independent repeats with similar results). **d**,**e**, Immunoblot for KDM4C, GRHL2 and CTSL in the indicated immunoprecipitants of KDM4C in SUM149 *GRHL2*^KO^ (**d**) and CTSL in *CTSL*^KO^ (**e**) derivates for one experiment. **f**, Genomic track view of GRHL2 (in parental cells) and CTSL ChIP–seq signal in SUM149 sgScramble and *GRHL2*^KO^ models at the *ASAP3* and *NFATC4* loci. **g**, Immunoblot for C′-H3 and GRHL2 of SUM149 sgScramble and *GRHL2*^KO^ models following 1 µM of QC6352 treatment for 5 days with tubulin as a loading control (two independent repeats with similar results). **h**, Intensity plot depicting GRHL2 ChIP–seq signal in vehicle and ML324-treated SUM149 cells on lost CTSL binding sites. **i**, Venn diagram showing the intersection of upregulated or downregulated differentially expressed genes associated with triple (KDM4C + GRHL2 + CTSL) and double (GRHL2 + CTSL) overlap peaks. **j**, Dot plot depicting Hallmark signature enrichment predicted from top 300 triple (KDM4C + GRHL2 + CTSL) and double (GRHL2 + CTSL) overlap peaks-associated genes. (Fisher’s exact test using Enrichr). **k**,**l**, Immunoblot for GRHL2 in 10% input and immunoprecipitants of pan-lysine methylation and IgG antibody in *KDM4C*-amplified HCC1954 (**k**) and *KDM4C* non-amplified HCC1806 and HDQP1 cells (**l**) grown in the indicated conditions from one experiment. **m**, Annotated mass spectra for methylated lysine 94 and 453. Lowercase amino acids indicate these are modified (**c** is alkylated cysteine, and **k** is monomethylated lysine). **n**, Immunoblot of GRHL2 in SUM149 cells following 3 days of siRNA transfection targeting the 3′UTR region of GRHL2, following 3 days of WT or mutant GRHL2 lentiviral infection (two independent repeats with similar results). Target proteins from IP experiments were quantified by normalizing to the corresponding input and labeled below each band. Source data

Next, we investigated how CTSL is recruited to the chromatin because it does not have a known DNA-binding domain. We first performed rapid immunoprecipitation MS (RIME)^37^ for KDM4C in control and ML324-treated cells but did not detect any CTSL peptides, suggesting that KDM4C may not directly bind CTSL or it is not detectable by this technique (Supplementary Table 3). To analyze CTSL-associated proteins in an unbiased manner, we performed MS of immunoprecipitated CTSL in SUM149 cells at baseline (Dox-inducible sh*KDM4C* #17 and #20 lines without Dox induction). CTSL itself and known CTSL-interacting proteins, including CTS3 and CTSB cysteine protease inhibitors, were among the most abundant peptides consistently enriched in CTSL immunoprecipitants compared to IgG controls (Fig. 4a, Extended Data Fig. 7a and Supplementary Table 4). We detected very few previously uncharacterized CTSL binding proteins, including the GRHL2 transcription factor and DEAD-Box helicase 23. Downregulation of KDM4C did not alter the interaction of CTSL with any of these proteins (Extended Data Fig. 7a and Supplementary Table 4). Intriguingly, GRHL2 was also the top predicted transcription factor motif enriched in CTSL peaks in HCC1954 and SUM149 cells (Fig. 4b).

We confirmed the association of GRHL2, CTSL and KDM4C in the nucleus by co-immunoprecipitation experiments for each of the three proteins (Fig. 4c) and by multicolor immunofluorescence analyses (Extended Data Fig. 7b). Notably, deletion of GRHL2 in SUM149 cells using CRISPR–Cas9 led to the loss of CTSL in KDM4C immunoprecipitants (Extended Data Fig. 7c,d), and deletion of CTSL led to the loss of GRHL2 and KDM4C in CTSL immunoprecipitants (Extended Data Fig. 7e), excluding the possibility that the observed results are due to the cross-reactivity of CTSL antibody with GRHL2 or KDM4C.

To verify the colocalization of CTSL and GRHL2 on the chromatin, we performed GRHL2 ChIP–seq and found that nearly all CTSL binding sites overlapped with GRHL2 peaks in both HCC1954 and SUM149 cell lines (Fig. 4d). Furthermore, *GRHL2*KO in SUM149 cells led to the nearly complete loss of CTSL chromatin binding (Fig. 4e and Extended Data Fig. 7f). Deletion of GRHL2 also diminished KDM4C-inhibition-induced histone H3 cleavage, again confirming the role of nuclear CTSL in this process (Extended Data Fig. 7g). GRHL2 peak signal was not markedly affected by ML324 treatment at CTSL sites lost after ML324 treatment, confirming direct GRHL2 chromatin binding independent of CTSL (Extended Data Fig. 7h). We also analyzed overlap in binding between KDM4C and GRHL2 peaks and CTSL sites lost after ML324 treatment. Although 38% of ML324-induced lost CTSL peaks (effective CTSL sites) overlapped with both GRHL2 and KDM4C (triple overlap), the CTSL peak intensity was substantially lower in these regions compared to only CTSL–GRHL2 overlapping peaks (Fig. 4f), implying that the GRHL2–CTSL complex may have KDM4C-independent functions. Both triple (KDM4C/GRHL2/CTSL) and double (GRHL2/CTSL) overlapping peaks were substantially associated with repression in gene expression after KDM4C blockade (Fig. 4g), and the DEGs and enriched pathways showed considerable overlap (Extended Data Fig. 7i,j). Thus, KDM4C inhibition may alter gene expression via CTSL-mediated histone cleavage through GRHL2 recruitment in both a direct and indirect manner.

Next, we explored potential mechanisms by which KDM4C inhibition induces CTSL-dependent histone H3 cleavage. We hypothesized that either GRHL2 or CTSL is a non-histone substrate of KDM4C and KDM4C inhibition leads to its increased methylation, which then triggers CTSL-mediated histone H3 clipping. To test this hypothesis, we first performed immunoprecipitation using a pan-lysine methylation antibody, followed by immunoblot for GRHL2 or CTSL after KDM4C knockdown or inhibition. Cells were grown in complete medium or lacking methionine to decrease the levels of intracellular *S*-adenosyl methionine (SAM), a cofactor required for protein methylation. We detected GRHL2 in pan-lysine methyl antibody immunoprecipitants after KDM4C blockade and only in *KDM4C*-amplified cells growing in complete medium (Fig. 4h and Extended Data Fig. 7k,l). To identify the specific methylation sites in GRHL2, we performed MS on in-gel digested GRHL2 immunoprecipitants from QC6352-treated SUM149 cells. We identified two lysine monomethylated sites in GHRL2 at residues K94 and K453 (Fig. 4i and Extended Data Fig. 7m). To determine the functional relevance of these GRHL2 lysine methylation events, we exogenously expressed WT GRHL2 or its single or double lysine-to-arginine mutant variants (that is, K94R, K453R and K94R/K453R) that cannot be methylated^38^, following the downregulation of endogenous GHRL2 using 3′UTR-targeting siRNAs (Extended Data Fig. 7n). We found that endogenous GRHL2 knockdown diminished both KDM4C-inhibition-induced histone H3 clipping and CTSL activation, which could be rescued by exogenous expression of the WT GRHL2 or the GRHL2^K94R^ mutant (Fig. 4j–l). In contrast, expression of GRHL2^K453R^ or GRHL2^K94R/K453R^ double mutant was unable to rescue the GRHL2-loss phenotype (Fig. 4j–l). These data demonstrate that mono-methylation of GRHL2 at K453 is required for KDM4C-loss-induced CTSL activation and histone H3 clipping.

### Metabolic shift from KDM4C inhibition aids histone clipping

**Fig. 5:**
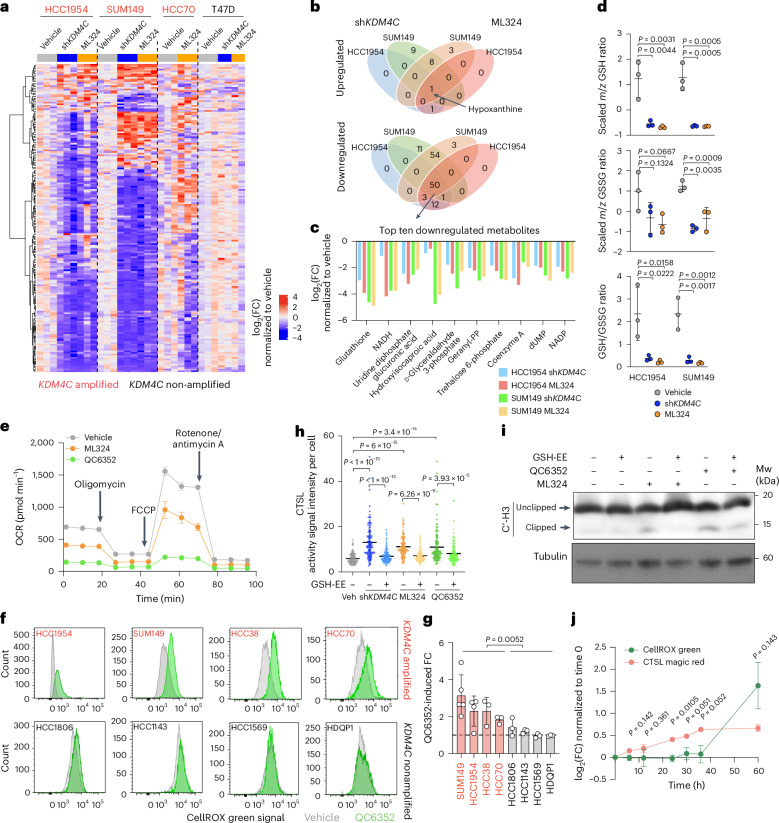
KDM4C blockade causes redox imbalance that activates CTSL. **a**, Heatmap showing clustering of 248 polar metabolites in Dox-inducible sh*KDM4C-*infected HCC1954, SUM149 and T47D cells following control (no Dox, DMSO), sh*KDM4C* induction (1 μg ml^−1^ Dox, DMSO) or 10 μm ML324 (no Dox) treatment and in HCC70 parental cells with or without 10 μm ML324 treatment. Metabolite abundances in each condition were normalized to the mean value of vehicle group of each cell line. **b**, Venn diagrams showing intersections of upregulated or downregulated metabolites in sh*KDM4C*-expressing HCC1954 and SUM149 cells with either Dox (sh*KDM4C*) or ML324 treatment. **c**, Bar plot representing the top ten consistently decreased metabolites in sh*KDM4C*-expressing HCC1954 and SUM149 cells with either Dox or ML324 treatments. **d**, Dot plots depicting normalized reduced (GSH) and oxidized (GSSG) GSH levels and their ratios in HCC1954 and SUM149 cell lines with sh*KDM4C* or ML324 treatment. Mean ± s.d. from *n* = 3 is shown. Dunnett’s test (two-sided) was used. *m*/*z*, mass-to-charge ratio of ions. **e**, Line plot depicting oxygen consumption rate (OCR) changes recorded by seahorse mito-stress assay in SUM149 cell lines treated with DMSO, 10 μm ML324 or 1 μm QC6352 for 3 days. Three time points were recorded for each state. This experiment was repeated three times independently with similar results. **f**, Representative flow cytometry plots depicting the shift of CellROX green signal in 8 cell lines after 1 μm QC6352 for 5 days. **g**, Bar plot showing QC6352-induced FCs in CellROX green signal merging five (SUM149), four (HCC1954) and three (all the other cell lines) independent experiments of each cell line (mean ± s.d.). Mann–Whitney *U* test was used to compare average FCs between four *KDM4C*-amplified and four non-amplified cell lines. **h**, Dot plot depicting quantification of CTSL activity signal quantified from 120 individual cells from 3 representative fluorescence images of inducible sh*KDM4C*-expressing SUM149 cells treated with DMSO (vehicle), 1 μg ml^−1^ Dox (sh*KDM4C*), 10 μm ML324 or 1 μm QC6352 or combined with 2 mM GSH-EE for 5 days. Mean and s.d. are shown. Statistical significance of differences was determined by two-sided ordinary one-way ANOVA. **i**, Immunoblot analysis of histone H3 using antibodies for the C terminus in SUM149 cells treated with 1 µM of QC6352 in the presence or absence of 2 mM GSH-EE for 3 days. Tubulin was used as a loading control. Experiment was repeated three times independently with similar results. **j**, Line plot illustrating the QC6352-induced log_2_(FCs) of CTSL magic red and CellROX green signals at the indicated time points in SUM149 cells. Data represent mean ± s.d. merged from three independent experiments. Two-sided two-way ANOVA at each time point was used for statistical comparison. Source data

**Extended Data Fig. 8:**
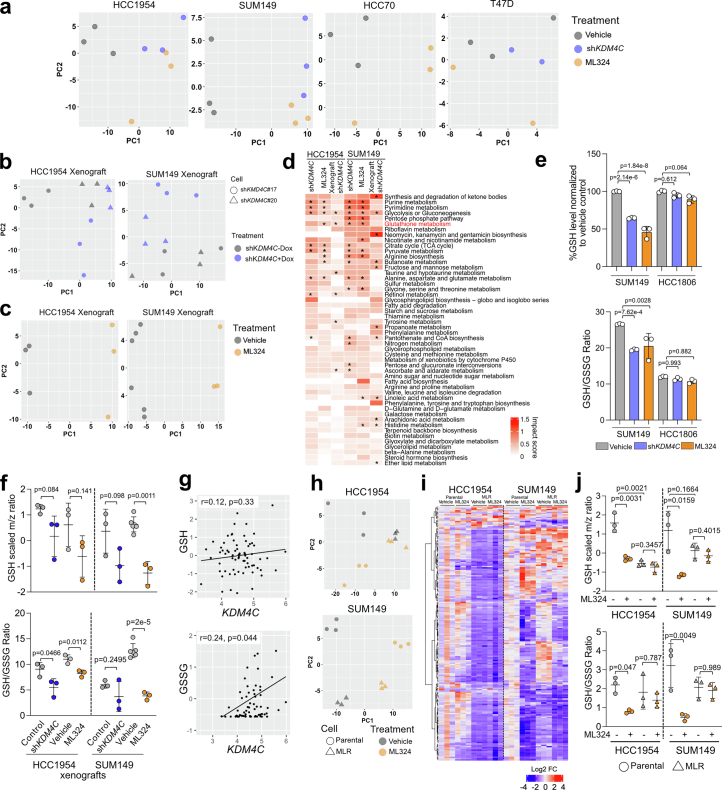
KDM4C-blockade-induced metabolic alteration. **a**–**c**, Principal component analysis plots of metabolomic profiles of the indicated doxycycline-inducible sh*KDM4C* models treated with vehicle (DMSO, no doxycycline), 0.1 μg/ml doxycycline (Dox) or 10 μm ML324 (no doxycycline) for 5 days (**a**), HCC1954 and SUM149 doxycycline-inducible sh*KDM4C* cells-derived xenografts with regular (−Dox) or doxycycline (+Dox) diets (**b**) or vehicle and ML324 treatments (**c**). **d**, Heatmap illustrating the impact scores of metabolic pathways significantly altered by doxycycline (sh*KDM4C*) or ML324 treatment after integration of metabolomics and RNA-seq data in cell lines or in tumors. Metabolic pathways were ranked starting from the strongest consistent impact (asterisk: pathways with p value < 0.05). **e**, Bar plots showing intracellular GSH levels and GSH/GSSG ratio in SUM149 and HCC1806 inducible-sh*KDM4C*-expressing cell models treated vehicle (DMSO), 1 μg/ml doxycycline or 10 μm ML324 for 2 days. Data are presented as mean ± s.d. from n = 3 from one experiment (two-sided ordinary one-way ANOVA). **f**, Dot plot showing GSH levels and GSH/GSSG ratio measured by mass spectrometry in HCC1954 and SUM149-inducible sh*KDM4C* model-derived xenografts from mice fed regular (control) or doxycycline diets (sh*KDM4C*) or treated with vehicle or ML324. Data represent mean ± s.d. with n = 6 tumors (SUM149 vehicle group) or n = 3 tumors for all the other groups (two-sided Student’s t test). **g**, Scatter plot representing correlations between *KDM4C* mRNA expression and GSH or GSSG abundance in 72 TNBC tumors profiled in the FUSCC cohort^44^. R and p values were derived from two-sided Pearson correlation. **h**,**i**, Principal component analysis plots (**h**) and a heatmap of unsupervised clustering of 248 polar metabolites (**i**) of metabolomic profiles of HCC1954 and SUM149 parental or ML324-resistant (MLR) models treated with DMSO or 10 μm ML324 for 5 days. Metabolite abundances in each condition were normalized to the mean value of vehicle group of each parental cell line. **j**, Dot plots depicting the GSH levels and GSH/GSSG ratio measured by mass spectrometry in HCC1954 and SUM149 parental or MLR models treated with DMSO or 10 μm ML324 for 5 days. Data represent mean ± s.d. of n = 3 biological replicates per group (two-sided ordinary one-way ANOVA). Source data

Our RNA-seq data identified multiple altered metabolic pathways upon KDM4C blockade (Fig. 1b), and the activity of both KDM4C and CTSL is regulated by metabolic factors. KDM4C is an oxygen- and α-ketoglutarate-dependent enzyme^39^, while optimal CTSL activity is at pH 3.0–6.5 when thiol compounds are present^40^. To test the hypothesis that CTSL-mediated histone clipping is also regulated by KDM4C-associated metabolic changes, we performed polar metabolite profiling in four cell lines (SUM149, HCC1954, HCC70 and T47D) and SUM149 and HCC1954 mouse xenografts with and without KDM4C blockade. Clustering of 248 metabolites showed widespread metabolomic changes by KDM4C inhibition in the three *KDM4C*-amplified basal cell lines (Fig. 5a, Extended Data Fig. 8a and Supplementary Table 5) and xenografts (Extended Data Fig. 8b,c), whereas minimal differences were detected in the luminal ER^+^ *KDM4C*-non-amplified T47D cell line (Fig. 5a and Extended Data Fig. 8a). Intersection of upregulated metabolites in both HCC1954 and SUM149 cell lines and KDM4C-blockade treatment conditions revealed hypoxanthine as the single overlap (Fig. 5b), while 50 metabolites were commonly downregulated including reduced glutathione (GSH) as the top affected and other metabolites involved in GSH metabolism (Fig. 5c). Both reduced (GSH) and oxidized GSH (GSSG) and their ratio (GSH/GSSG) were decreased upon KDM4C blockade (Fig. 5d), suggesting an overall redox imbalance due to suppression of GSH biosynthesis pathway. Integration of metabolomic and transcriptomic changes also identified GSH metabolism as the top consistently downregulated pathway in both *KDM4C*-amplified cell lines and tumors along with glycolysis, gluconeogenesis and pentose phosphate pathways (Extended Data Fig. 8d). In line with this observation, KDM4C inhibition dampened general mitochondrial respiration (Fig. 5e), consistent with previous reports on outcomes of redox imbalance^41,42^.

We confirmed a decrease in GSH and GSH/GSSG ratio following KDM4C downregulation or inhibition by luminescence assays in SUM149 cells (Extended Data Fig. 8e). A similar trend but higher variability was observed in metabolomic profiles in HCC1954 and SUM149 xenografts (Extended Data Fig. 8f). A substantial positive association was also observed between *KDM4C* mRNA and GSSG levels in 72 TNBC tumor samples from a recent multi-omic profiling study^43^ validating the clinical relevance of our findings (Extended Data Fig. 8g). GSH showed a similar trend but no substantial association potentially due to high levels of reactive oxygen species (ROS) converting GSH to GSSG. Metabolomic profiling of HCC1954-MLR and SUM149-MLR resistant models depicted cell line-dependent differences, with HCC1954-MLR cells showing ML324-induced reduction in many metabolites even at baseline level and no further changes after ML324, while SUM149-MLR cells showed higher baseline and ML324-induced differences (Extended Data Fig. 8h,i). ML324 did not affect GSH levels and GSH/GSSG ratios in MLR cells, indicating their potential role associated with resistance development, and HCC1954-MLR cells particularly have lower baseline GSH levels than parental cells (Extended Data Fig. 8j). These data imply that KDM4C may be a key modulator of redox balance in *KDM4C*-amplified basal breast cancer cell lines through the GSH pathway.

**Extended Data Fig. 9:**
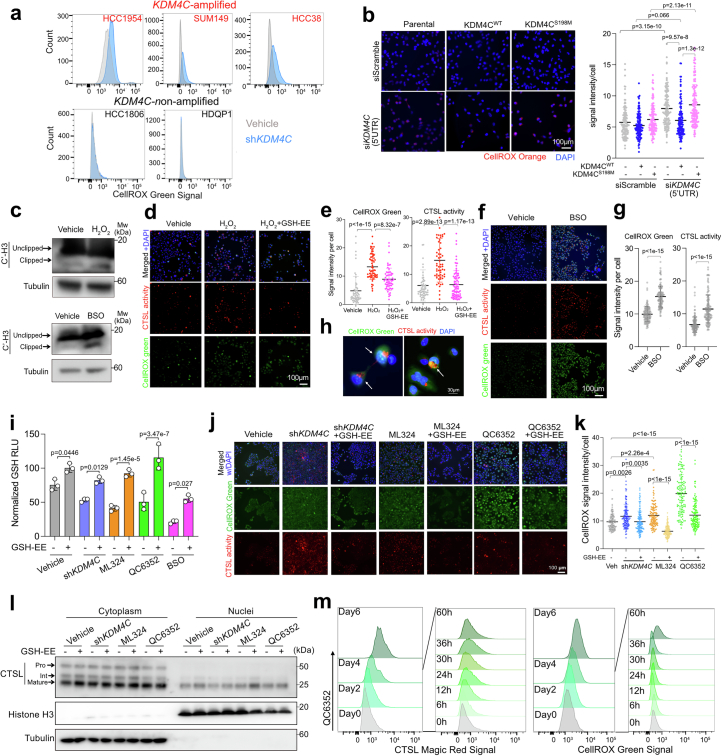
KDM4C-blockade-induced redox imbalance aids histone clipping. **a**, Plots showing the CellROX green signal of five inducible-sh*KDM4C* models treated with or without 1 μg/ml doxycycline for 5 days. **b**, Representative images (left) and quantification (right) of CellROX orange signal in SUM149 cells overexpressing KDM4C^WT^ and KDM4C^S198M^ with siRNA against *KDM4C* 5′UTR for 5 days. Mean ± s.d. of 120 cells from three to four representative regions are shown (two-sided ordinary one-way ANOVA). Scale bar, 100 μm. **c**, Immunoblot for C′-H3 from SUM149 cells treated with 0.5 mM H_2_O_2_ for 1 day (top) or 1 μm BSO for 3 days (bottom; two (BSO) and three (H_2_O_2_) independent repeats with similar results). **d**,**f**, Representative images of CTSL activity and CellROX green signal in SUM149 cells treated with water, 0.5 mM H_2_O_2_ with or without 2 mM GSH-EE for 1 day (**d**) or 1 μm BSO for 3 days (**f**). Scale bar, 100 μm. **e**,**g**, Quantification of **d** and **f**. Mean ± s.d. are shown from 60 (**e**) or 120 (**g**) cells from three representative regions of each condition (two-sided ordinary one-way ANOVA for **e** and Student’s t test for **g**). **h**, Magnified images of H_2_O_2_-treated SUM149 cells. Overlapped CTSL and ROS nuclei signals are highlighted. Scale bar, 30 μm. **i**, Bar plot showing intracellular GSH levels in inducible-sh*KDM4C* SUM149 cells with DMSO, 1 μg/ml doxycycline (sh*KDM4C*), 10 μm ML324 or 1 μm QC6352 with or without 2 mM GSH-EE for 2 days normalized to the corresponding cell numbers. Mean ± s.d. are shown from n = 3 from one experiment (two-sided ordinary one-way ANOVA). **j**, Representative images of CTSL activity and CellROX green signal under the same conditions as **i** for 5 days. Scale bar, 100 μm. **k**, Mean ± s.d. are shown for ROS signal of 120 cells from three representative regions (two-sided ordinary one-way ANOVA). **l**, Immunoblot of CTSL isoforms in different fractions of SUM149 cells under the same conditions as **i** for 5 days with tubulin and histone H3 as loading controls (three independent repeats with similar results). **m**, Plots showing CTSL activity and CellROX green signals in SUM149 cells treated with 1 μm QC6352 for the indicated time. Representative experiment from three independent repeats is shown. Source data

Next, we investigated the interplay between KDM4C-inhibition-induced reduction in GSH levels and CTSL-mediated histone H3 tail clipping. Similar to the pattern of histone cleavage and CTSL activation, KDM4C inhibition or downregulation induced a more pronounced elevation of ROS in *KDM4C*-amplified basal breast cancer cell lines (Fig. 5f,g and Extended Data Fig. 9a). Increased ROS activity following KDM4C blockade was rescued by overexpression of WT but not catalytically inactive-mutant KDM4C (Extended Data Fig. 9b). Elevation of ROS levels by direct stimulation of H_2_O_2_ or blockade of GSH biosynthesis by glutamate–cysteine ligase inhibitor buthionine sulfoximine (BSO) increased histone cleavage (Extended Data Fig. 9c) and CTSL activity (Extended Data Fig. 9d–g), and high ROS and active CTSL were detected in the same cells (Extended Data Fig. 9h). In contrast, neutralization of ROS by addition of GSH ethyl ester (GSE-EE), a cell-permeable form of GSH, efficiently reduced CTSL activity in SUM149 cells (Fig. 5h and Extended Data Fig. 9i–k). GSH-EE treatment also reduced QC6352-induced histone H3 clipping in SUM149 cells (Fig. 5i). We also tested if KDM4C blockade or GSE-EE administration changes the CTSL maturation process. Immunoblot analysis following cell fractionation did not show a notable difference in mature CTSL ratios across the treatments in cell nuclei and cytoplasm (Extended Data Fig. 9l), suggesting that the observed CTSL activation changes were not due to altered CTSL maturation.

Given that both ROS and KDM4C–GRHL2 interaction trigger CTSL activation and histone tail clipping, we conducted a time course experiment to determine the temporal order of these events. We found that both ROS and CTSL activity showed a gradual increase during 6 days of KDM4C inhibitor treatment, but CTSL activation occurred as early as 24 h after treatment, while ROS levels started to elevate between 36 and 60 h (Fig. 5j and Extended Data Fig. 9m). These data suggest that an increase in GRHL2 K453 methylation following KDM4C inhibition might serve as an initial trigger of CTSL activation, while GSH repression and ROS induction are downstream events that further boost CTSL activity through positive feedback.

### KDM4C inhibition decreases GCLC, leading to redox imbalance

**Fig. 6:**
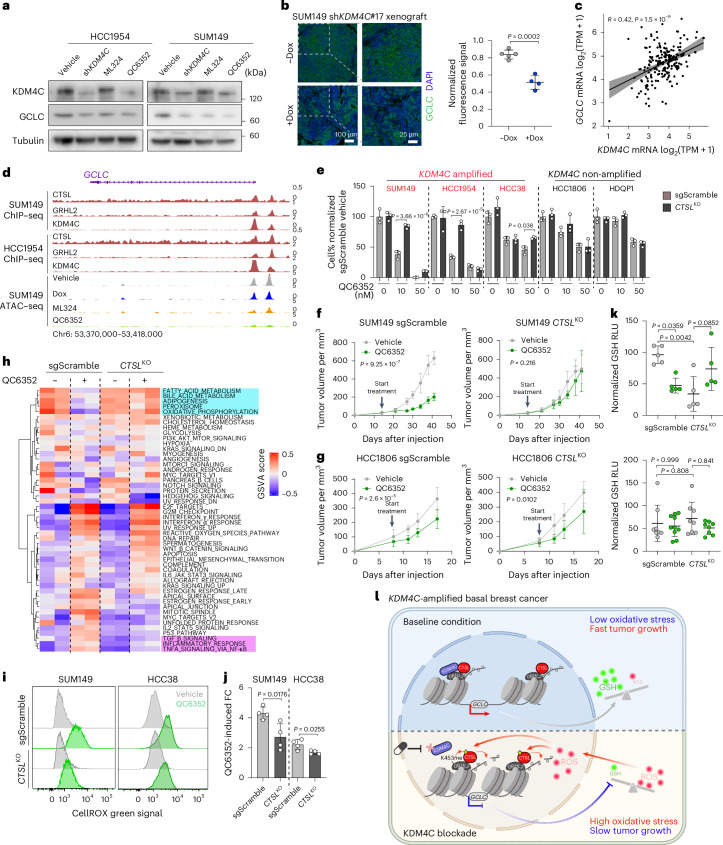
KDM4C blockade decreases *GCLC* expression via CTSL. **a**, Immunoblot analysis of KDM4C and GCLC protein levels in HCC1954 and SUM149 Dox-inducible sh*KDM4C*-expressing cell lines treated with control (no Dox, DMSO), sh*KDM4C* induction (1 μg ml^−1^ Dox, DMSO), 10 μm ML324 (no Dox) or 1 μm QC6352 (no Dox) treatment for 5 days. Tubulin was used as loading control. Experiment was repeated three times independently with similar results. **b**, Representative images of GCLC immunofluorescence staining of xenografts derived from SUM149 cells expressing Dox-inducible sh*KDM4C* from mice fed with (*n* = 4) or without (*n* = 5) Dox diet. Signal intensity of each tumor was quantified by calculating the mean of three representative regions and shown as mean ± s.d. Two-sided Student’s *t* test was used. **c**, Scatter plot depicting correlation between *KDM4C* and *GCLC* mRNA levels in 190 basal breast tumors from the TCGA cohort. Two-sided Pearson correlation was used to calculate the *P* value. The linear regression line with 95% confidence interval is shown. TPM, transcripts per million. **d**, Genomic track view of CTSL, GRHL2 and KDM4C binding in HCC1954 and SUM149 cells at *GCLC* genomic locus. ATAC peaks from Dox-inducible sh*KDM4C*-expressing SUM149 cells treated with vehicle, Dox, ML324 and QC6352 are also displayed using the same scaling. Chr6, chromosome 6. **e**, Bar plot showing the cell percentage normalized to sgScramble cell models treated with DMSO in the indicated groups. Results are shown as mean ± s.d. from *n* = 3 as representative experiments from at least 2 independent trials. Two-sided ordinary one-way ANOVA was used within each cell line. **f**,**g**, Plots depicting the tumor volumes of xenografts derived from SUM149 (**f**) and HCC1806 (**g**) sgScramble and *CTSL*^KO^ cells in mice treated with vehicle or QC6352 at the indicated time points. Data are presented as mean ± s.d. with *n* = 5 (SUM149) and *n* = 10 (HCC1806) tumors. Two-sided repeated-measure two-way ANOVA was used to compare the tumor growth kinetics. **h**, Heatmap illustrating unsupervised clustering of samples based on the GSVA enrichment scores of the 50 hallmark gene signatures. QC6352 upregulated and downregulated pathways that were rescued by CTSL depletion are highlighted by magenta and cyan rectangles, respectively. **i**, Representative flow cytometry plots depicting the shift of CellROX green signal in SUM149 and HCC38 sgScramble and *CTSL*^KO^ models after 1 μm QC6352 for 5 days. **j**, Bar plot depicting QC6352-induced CellROX green FCs merging three independent experiments (mean ± s.d.). Two-sided Student’s *t* test was used. **k**, Dot plot depicting GSH levels normalized to tumor weight in SUM149 and HCC1806 xenografts collected at endpoint. Data are presented as mean ± s.d. with *n* = 5 (SUM149) or *n* = 10 (HCC1806) tumors. Two-sided Kruskal–Wallis test was used for each comparison. RLU, relative light units. **l**, Schematic illustration of major findings. HDM KDM4C blocks GRHL2-mediated CTSL activation and histone H3 tail clipping, which have a pivotal role in redox balance via maintaining GSH production and promoting basal breast tumor growth. KDM4C blockade activates CTSL either directly or indirectly and induces redox imbalance, which elevates oxidative stress and impairs basal breast tumor growth. Panel **l** created with BioRender.com. Source data

**Extended Data Fig. 10:**
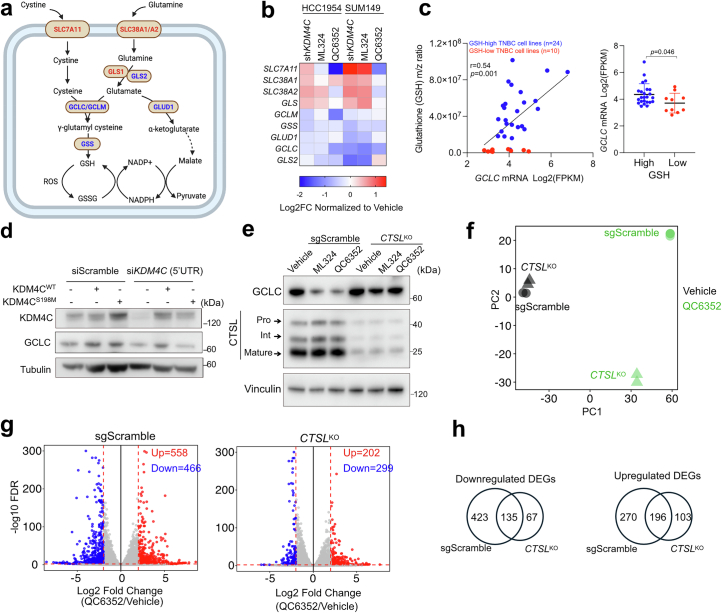
KDM4C suppression triggers redox imbalance via decreasing *GCLC.* **a**, Schematic view of glutathione synthesis pathway. Enzymes or transporters analyzed in **b** are indicated with red and blue representing increase and decrease upon KDM4C blockade, respectively. **b**, Heatmap showing fold change in expression of nine key enzymes or transporters involved in glutathione biosynthesis in HCC1954 and SUM149 Dox-inducible shKDM4C cell lines following treatment with 0.1 μg/ml doxycycline (Dox), 10 μm ML324 or 1 μm QC6352 for 5 days. Gene expression was normalized to the corresponding vehicle controls. **c**, Left: scatter plots showing the correlation of *GCLC* expression with GSH abundance from 34 TNBC cell lines. R and p values were derived from two-sided Pearson correlation. Right: dot plot depicting mean ± s.d. of *GCLC* expression (log_2_(FPKM)) in GSH-high (n = 24) and GSH-low (n = 10) TNBC cell lines (two-sided Mann–Whitney U test). **d**, Immunoblot for KDM4C and GCLC in SUM149 cells overexpressing KDM4C^WT^ and KDM4C^S198M^ with siRNA against *KDM4C* 5′UTR for 3 days with tubulin as a loading control (two independent repeats with similar results). **e**, Immunoblots showing CTSL and GCLC protein levels in SUM149 sgScramble and *CTSL*^KO^ cell lines treated with or without 10 μm ML324 or 1 μm QC6352 for 5 days, with vinculin as a loading control (three independent repeats with similar results). **f**, Principal component analysis plot of RNA-seq profiles of SUM149 sgScramble and *CTSL*^KO^ models treated with DMSO (vehicle) or 1 μm QC6352 for 3 days. **g**, Volcano plots showing QC6352-induced differentially expressed genes in SUM149 sgScramble and *CTSL*^KO^ models. DEGs were selected with adjusted p < 0.05 and log_2_(FC) > 2, and specific numbers were labeled on the plots. FDR values were calculated by the Wald test following Benjamini–Hochberg correction using DESeq2. **h**, Venn diagram illustrating the overlap of upregulated and downregulated DEGs induced by QC6352 in SUM149 sgScramble and *CTSL*^KO^ models. Source data

To further explore mechanisms underlying the KDM4C-inhibition-induced decrease in GSH, we compared the expression levels of key enzymes and transporters involved in GSH biosynthesis (Extended Data Fig. 10a) in our RNA-seq data. We found a decrease in the expression of several genes, including *GSS* (GSH synthetase), *GCLC* and *GCLM* (glutamate–cysteine ligase catalytic and modifier subunits, respectively; Extended Data Fig. 10b). Immunoblot analysis confirmed the strong and consistent downregulation of the rate-limiting enzyme GCLC in both HCC1954 and SUM149 cell lines after KDM4C downregulation or inhibition (Fig. 6a). The decrease in GCLC was also validated by immunofluorescence in SUM149 xenografts following Dox-induced *KDM4C* knockdown (Fig. 6b). GCLC is a subunit of the first rate-limiting enzyme for GSH synthesis coupling glutamate and cysteine into γ-glutamylcysteine, the precursor of GSH. Our prior transcriptomic and metabolomic profiling in 34 TNBC cell lines^44^ identified a positive correlation between *GCLC* and GSH levels, and GSH was the driver of metabolomic heterogeneity in TNBC, splitting samples into low and high groups (Extended Data Fig. 10c). Furthermore, the mRNA levels of KDM4C and GCLC show a significant positive correlation in basal breast cancer in the TCGA cohort (Fig. 6c), implying coregulation in clinical samples.

We next investigated how KDM4C might regulate *GCLC* expression. First, exogenous expression of WT but not catalytically inactive-mutant KDM4C rescued KDM4C knockdown-induced GCLC downregulation, confirming the specificity of this observation to KDM4C (Extended Data Fig. 10d). Our ChIP–seq data demonstrated a consistent triple overlap of CTSL, GRHL2 and KDM4C peaks at the *GCLC* promoter region in both SUM149 and HCC1954 cell lines, and KDM4C inhibition markedly reduced chromatin accessibility of this genomic region (Fig. 6d), implying that *GCLC* downregulation is a potential outcome of CTSL-mediated histone H3 tail clipping. This finding was supported by immunoblot analyses in *CTSL*^KO^ cells, where KDM4C inhibition failed to dampen GCLC expression (Extended Data Fig. 10e). Deletion of CTSL also eliminated KDM4C-inhibition-induced tumor growth suppression specifically in *KDM4C*-amplified cell models where CTSL-mediated histone H3 clipping is observed (Fig. 6e–g). RNA-seq revealed less pronounced QC6352-induced transcriptomic alterations in SUM149 *CTSL*^KO^ cells (Extended Data Fig. 10f–h and Supplementary Table 6). Notably, *CTSL* KO rescued a subset of gene signatures that were altered by QC6352 in sgScramble control cells, including major metabolic functions such as oxidative phosphorylation and fatty acid metabolism (Fig. 6h). In line with this observation, KDM4C suppression-associated ROS elevation was partially rescued in *KDM4C*-amplified *CTSL*^KO^ cells (Fig. 6i,j). Finally, KDM4C inhibition also failed to decrease GSH levels in xenografts derived from *CTSL*^KO^ SUM149 cells, despite a lower baseline level compared to scramble control (Fig. 6k), while no GSH level changes were detected in HCC1806 *KDM4C*-non-amplified cell line xenografts (Fig. 6k).

Taken together, these results identified the CTSL–GCLC axis as a key mediator of KDM4C-loss-associated metabolomic and epigenetic remodeling and tumor growth suppression in *KDM4C*-amplified basal breast cancer.

### Targeting the KDM4C–GSH–CTSL axis in basal breast cancer

To explore the potential clinical relevance of our findings, we generated KDM4C and GSH modulated gene signatures using DEGs associated with *KDM4C* knockdown in HCC1954 and SUM149 cell lines (*n* = 23 genes) and GSH-high versus GSH-low TNBC cell lines (*n* = 113 genes) defined in our previous study^44^, respectively (Supplementary Fig. 2a and Supplementary Table 7). Strong positive correlation between GSH and KDM4C signatures was also observed in basal primary tumors in both TCGA and METABRIC cohorts^22,23^ (Supplementary Fig. 2b), recapitulating our findings in cell line models. Furthermore, oxidative stress signature was inversely correlated with KDM4C signature (Supplementary Fig. 2c), in line with our experimental results of dampened mitochondrial respiration and increased ROS levels following KDM4C inhibition

The current standard of care for patients with TNBC is chemotherapy with or without immune checkpoint inhibitors^2^. Thus, predictors of patient survival are likely associated with chemotherapy resistance. We examined the enrichment levels of KDM4C and GSH signatures between responders and non-responders in five TNBC neoadjuvant patient cohorts treated with different chemotherapies^45–50^. Although the KDM4C and GSH signatures showed a significant positive correlation in all five cohorts, differences between responders and non-responders were only significant in a neoadjuvant cisplatin-treated cohort^46^ (Supplementary Fig. 2d). In line with this finding, *KDM4C* copy number amplification was uniquely correlated with a lower trend of cisplatin sensitivity in basal cell lineage in the DepMap^51^ breast cancer cell line data, while response to paclitaxel, 5-fluorouracil and doxorubicin was not correlated (Supplementary Fig. 2e).

To experimentally validate the prediction that cotargeting KDM4C and GSH pathways could overcome cisplatin resistance, we tested the effects of combined cisplatin, BSO and QC6352 treatment in six basal breast cancer cell lines with varying *KDM4C* amplification and *BRCA1* mutation status and GSH metabolic subtypes (Supplementary Fig. 3a). QC6352 and cisplatin combination showed synergistic growth suppression only in HCC3153 cells (Supplementary Fig. 3b,c), and synergism between QC6352 and cisplatin was strengthened with increasing concentration of BSO in a dose-dependent manner in two other *KDM4C*-amplified basal cell lines, SUM149 and HCC38 (Supplementary Fig. 3d,e). In contrast, GSH-low cells (HCC70 and MDA-MB-436) and a HER2-amplified basal breast cancer cell line (HCC1954) showed substantial antagonism (Supplementary Fig. 3f–h), likely due to their limited dependency on the GSH pathway. KO of *CTSL* in the SUM149 cell line abolished the enhancement of QC6352 and cisplatin synergy by BSO, again confirming the requirement for CTSL to mediate the metabolic effects of KDM4C inhibition (Supplementary Fig. 3i). Finally, testing single, dual and triple combinations in the SUM149 xenograft model showed that combination of either QC6352 or BSO with cisplatin was sufficient to suppress GSH production and tumor growth (Supplementary Fig. 3j–n). These results suggest that combined inhibition of KDM4C and GSH production might overcome cisplatin resistance in a subset of patients with high GSH pathway activity, encouraging further clinical examination.

## Discussion

The frequent genetic alteration of genes encoding histone-modifying enzymes in human cancers implies their role as functional drivers of tumorigenesis. Here we characterized KDM4C, an HDM frequently amplified in basal breast cancer, and discovered a unique role for KDM4C in *KDM4C*-amplified basal breast cancer in regulating CTSL-mediated histone H3 N-terminal tail clipping through modulating lysine methylation of the GRHL2 transcription factor (Supplementary Table 8).

Reversible post-translational modifications of histone H3 have been extensively characterized, whereas the biological consequences of irreversible histone tail clipping have remained elusive. The tailless histone H3 was shown to precede histone eviction^52,53^, and blocking CTSG-, ELANE- and PRTN3-mediated histone 3 tail cleavage at Ala21 dominantly induced chromatin opening in monocytes promoting monocyte-to-macrophage differentiation^54^, consistent with our finding that H3 tail clipping leads to decreased chromatin accessibility. Tailless histone H3 could also potentially activate gene expression due to reduced steric effects on the nucleosome and could facilitate recruitment of transcriptional machinery. It may also provide additional docking space for other histone modifiers, such as the PRC2 complex or the KMT5 histone H4K20 methyltransferase, which may explain higher H3K27me3 and H4K20me3 abundance after KDM4C inhibition. Another study revealed that N-terminally truncated histone H3 interferes with intratail H3K36me3 regulation^55^, which could in part explain our inability to detect substantial changes in H3K36me3 following KDM4C inhibition, although the cleavage occurred at Ala21. The effects of histone H3 tail clipping on the chromatin are likely to be context-dependent, because we observed cell line-specific differences in chromatin accessibility and transcriptional profiles following KDM4C inhibition and mechanisms of acquired ML324 resistance were also somewhat different between HCC1954 and SUM149 models. We also detected histone H4 cleavage upon KDM4C blockade that is regulated by a distinct mechanism, as it was universally observed in all cell lines, including the ER^+^ luminal T47D cells, and was blocked by aspartic acid protease inhibitors. Histone H4 clipping might shape the epigenome differently from that of H3; uncovering this requires future studies.

CTSL is a lysosomal protease, but a nuclear function for CTSL was also reported in cell cycle regulation via proteolytic cleavage of the CDP/Cux transcription factor^56^. Subsequently, CTSL was described as a nuclear protease that cleaves histone H3 during embryonic development^34,57^ and cellular differentiation^58^. Resolving the three-dimensional crystal structure of the CTSL–H3 peptide complex revealed the structural basis of this process^33^. However, the mechanism by which CTSL is recruited to the chromatin has been elusive. Here we identified the GRHL2 transcription factor as a chromatin recruiter of CTSL in *KDM4C*-amplified basal breast cancer cells. Furthermore, we discovered that GRHL2 is lysine methylated in a KDM4C activity-dependent manner, and its methylation at K453 is required for CTSL-mediated histone H3 clipping. Numerous transcription factors, including p53, E2F-1 and STAT3, have been shown to be methylated at arginine or lysine residues, which can modulate their ability to activate transcription^59,60^. In many cases, transcription factor methylation is triggered by environmental signals like DNA damage, and methylation/demethylation is carried out by histone methyltransferases and demethylases^59,60^. Our data suggest that GRHL2 might be a non-histone substrate of KDM4C, because KDM4C blockade increased its methylation. Our data show that KDM4C inhibition does not affect GRHL2–CTSL interaction. However, it is possible that a mono-methylation reader protein could also be part of the complex, or GRHL2 K453 methylation could directly or indirectly influence the catalytic site of CTSL via its inhibitors, cystatin B and cystatin C. Indeed, deletion of *Ctsb* in mice leads to increased nuclear CTSL activity and persistent cleavage of histone H3 in the brain^61^. Our results imply that GRHL2 K94 methylation may not have a direct role in regulating CTSL-mediated histone H3 clipping, but it may influence DNA binding given its proximity to the DNA-binding domain. Moreover, the observation of GRHL2 methylation only in *KDM4C*-amplified cells upon KDM4C blockade suggests distinct evolutionary paths in *KDM4C*-amplified tumors, with a high level of KDM4C keeping GRHL2 demethylated, thereby preventing histone clipping. Further studies are required to decipher the exact mechanisms by which KDM4C–GRHL2 regulates CTSL-mediated histone clipping and to delineate why this function is selected for in *KDM4C-*amplified basal breast cancers.

Our metabolomic profiling showed a substantial decrease in GSH, GSSG and their ratio after KDM4C inhibition. Previous studies have described that both ROS and GSH can extensively modulate the epigenome. For example, the accumulation of ROS reduces the availability of SAM, limiting the activities of DMNTs and HMTs, leading to global epigenetic alteration^62^. GSH may also directly affect the chromatin via S-glutathionylation of histone H3, destabilizing nucleosome structure and opening chromatin^63,64^. The GSH biosynthesis pathway has previously been identified as a therapeutic vulnerability in TNBC^65^ and inhibition of the glutamate–cystine antiporter xCT efficiently impaired the growth of glutamine auxotroph TNBC lines^66^. GSH also has a role in therapeutic resistance, and pharmacological depletion of GSH sensitizes cells to cisplatin^67,68^. Here we found that the KDM4C–CTSL axis is an alternative route to inhibit GSH production, which could yield synergism with cisplatin in a subset of basal breast tumors. Because cisplatin is more effective in DNA repair-defective (for example, *BRCA1* mutant) tumors^69^, our results suggest that combined targeting of KDM4C–GSH may be a putative therapeutic strategy in these patients.

A limitation of our study is that we did not perform quantitative ChIP–seq experiments using spike-in controls, and thus, the lack of global differences in H3K9me3 following KDM4C inhibition needs to be interpreted with caution. Our functional studies were performed in cell lines; therefore, the methylation of GRHL2 and its association with CTSL and KDM4C activity would need to be validated in primary patient samples. Finally, mechanisms that drive the evolution of *KDM4C*-amplified basal breast cancer to prevent GRHL2 methylation and histone H3 clipping by CTSL would need to be delineated in future studies.

In summary, we discovered a unique function of KDM4C specific to *KDM4C*-amplified basal breast cancer, elucidating the underlying mechanisms and clinical significance. Our results serve as a basis for the clinical testing of KDM4C inhibitors in *KDM4C*-amplified basal breast cancer, potentially in combination with chemotherapy and agents targeting the glutamine/GSH pathway.

## Methods

### Ethics statement

All human and animal studies were conducted in compliance with the relevant ethical guidelines and approved by the appropriate ethics committees as detailed below. All animal studies were conducted in accordance with the regulations formulated by the Dana-Farber Cancer Institute (DFCI) Animal Care and Use Committee protocol 11-023. Surgically resected breast tumor samples have been previously described^70,71^ and were collected at the Instituto Nacional de Enfermedades Neoplásicas (Lima, Perú) following institutionally approved protocol INEN 10-018. Written informed consent was obtained from all participants or waived for deceased patients. Samples were deidentified before transport to the laboratory.

### Breast cancer cell lines

Breast cancer cell lines were obtained from American Type Culture Collection (ATCC), Leibniz Institute DSMZ – German Collection of Microorganisms and Cell Cultures GmbH (DSMZ) or generously provided by outside academic institutions under Material Transfer Agreement (MTA) (see Supplementary Table 9 for details) and cultured following the provider’s recommendations. The identity of the cell lines was confirmed by short tandem repeat analysis, and they were regularly tested for mycoplasma. Details of the generation of cell line derivatives are described in Supplementary Note.

### Animal experiments

For xenograft assays using *KDM4C* knockdown HCC1954 and SUM149 models, as well as cisplatin/QC6352/BSO drug combination assay, female NCr nude (CrTac:NCr-Foxn1nu) mice were purchased from Taconic Biosciences at 5–6 weeks of age. For experiments using HCI-041 PDX, *KDM4C* knockdown HCC1806 and *CTSL*^KO^ SUM149 and HCC1806 models, female NSG (NOD.Cg-Prkdcscid Il2rgtm1Wjl/SzJ) mice were purchased from the Jackson Laboratory at 5–6 weeks of age. Mice were housed 5 to a cage with ad libitum access to food and water in 20 °C ambient temperature, 40–50% humidity and a 12-h light/12-h dark cycle. Experimental details are described in Supplementary Note.

### Immunoblot and immunoprecipitation assays

Details of immunoblot analyses are described in Supplementary Note. For immunoprecipitation, cells were cultured to 80% confluency in three 15 cm dishes, washed and collected in ice-cold PBS and then lysed in cytoplasmic lysis buffer (10 mM HEPES, 10 mM KCl, 1.5 mM MgCl_2_, 0.5% NP40 and 0.5 mM DTT) by rotating at 4 °C for 10 min. The lysates were centrifuged, and nuclear pellets were lysed in nuclear lysis buffer (20 mM HEPES, 300 mM NaCl, 1.5 mM MgCl_2_, 0.5% NP40, 10% glycerol and 0.2 mM EDTA) by rotating at 4 °C for 10 min. The samples were sonicated using a cup-probe sonicator for a total of 5 min with a 20 s on/10 s off cycle at 75% amplitude, followed by centrifugation. Supernatants were diluted twofold with dilution buffer (20 mM Tris–HCl (pH 8.0), 1.5 mM MgCl_2_, 0.5% NP40 and 0.2 mM EDTA). DNase I digestion was performed using Qiagen DNase I at 20 U ml^−1^ for 30 min at 37 °C. Each sample was divided into two for immunoprecipitation with 5 µg of specific antibody or isotype IgG control, both incubated overnight at 4 °C. Next, 25 µl of Pierce protein A/G magnetic beads were added to each sample and incubated for an additional 2 h at 4 °C. The samples were washed twice with low-salt washing buffer (20 mM Tris–HCl (pH 8.0), 150 mM NaCl, 1.5 mM MgCl_2_, 0.5% NP40 and 0.2 mM EDTA) and once with low Tris-EDTA buffer. The beads were resuspended in nuclear lysis buffer containing lithium dodecyl sulfate and a reducing reagent and heated at 95 °C for 5 min. The supernatants were used directly for immunoblotting, together with 10% input loading. Detailed antibody information is provided in Supplementary Table 9. Immunoblot intensity of each band was quantified using ImageJ (v1.53q) and labeled in the panels.

### Metabolomic profiling

Inducible sh*KDM4C*-infected HCC1954, SUM149, T47D and HCC70 parental cells were plated in duplicate in 3 biological replicates for each group following control (no doxycycline (Dox), DMSO), sh*KDM4C* induction (plus 0.1 μg ml^−1^ Dox) or 10 μm ML324 (ML, no Dox) treatment in the first 3 lines and with or without 10 μm ML324 treatment in HCC70 for 5 days. Polar metabolites were extracted as described^72^ (further details are given in Supplementary Note).

### Histone MS

Exponentially growing cells were collected by trypsinization, pelleted, washed and snap frozen. For inhibitor treatment, 100 μm AEBSF HCl, 100 μm pepstatin A, 10 μm SID2668150, 10 μm E64d and 5 μm cathepsin inhibitor III were applied for 24 h. Histone modification profiling was performed as described in ref. ^73^. Briefly, histones were extracted from cell nuclei by acid extraction and precipitated with trichloroacetic acid. Isolated histones (10 μg per sample) were propionylated, desalted and digested overnight with trypsin following standard protocols. A second round of propionylation was performed before desalting. Before MS analysis, a reference mixture of isotopically labeled synthetic peptides for histones H3 and H4 was added to each sample. Peptides were separated using a C18 column (Thermo Fisher Scientific, EASY-nLC 1000) and analyzed by MS using a parallel reaction monitoring method on a Q Exactive Plus Orbitrap (Thermo Fisher Scientific). Chromatographic peak areas of endogenous (light, L) and synthetic standard (heavy, H) peptides were extracted using Skyline, and L:H peak area ratios were calculated. These ratios were log_2_-transformed, normalized to an unmodified region of H3 (41–49) or H4 (68–78), row median normalized for each histone mark and further adjusted to the mean of vehicle groups. For peptide clipping identification, samples were analyzed using liquid chromatography MS (Proxeon EASY-nLC 1000 UHPLC and Q Exactive^+^ mass spectrometer) in two different ways. First, samples were injected using a targeted, parallel reaction monitoring acquisition method to strictly monitor for the presence of defined combinatorial forms of modified histone peptides. Second, samples were injected for a second time, and peptides were quantified using an unbiased, data-dependent acquisition (DDA) strategy that monitors for all peptides present in the sample. Results were analyzed independently using the Skyline software package (v4.0) and the Spectrum Mill (v7.0), respectively.

### Immunoprecipitation MS

For MS on CTSL immunoprecipitants, pulled-down proteins were digested on beads using trypsin digest buffer (2 M urea, 50 mM Tris–HCl, 2 mM DTT and 0.005 μg ml^−1^ trypsin) with shaking for 1 h at 25 °C. Supernatant was transferred to a cold tube, and the beads were washed twice with urea buffer (2 M urea and 50 mM Tris–HCl), combining the wash volumes with the original supernatant. This entire process, including the digestion, was repeated for a second time. Both digests from each sample were pooled. Each sample was then subjected to reducing conditions (5 mM dithiothreitol) to cleave disulfide bonds. Unmodified cysteine residues were then alkylated (10 mM iodoacetamide) to prevent the reformation of disulfides. Proteins were then digested into peptides using an overnight trypsin digest. Samples were isotopically labeled to multiplex the sample set, allowing for more robust cross-sample comparisons. Samples were analyzed via liquid chromatography MS (Proxeon EASY-nLC 1000 UHPLC and Q Exactive^+^ mass spectrometer), using an unbiased, DDA strategy. Results were processed using Spectrum Mill. Proteins were filtered based on the criteria that they must include two or more unique human peptides. Results were interpreted using the ProTIGY interactive visualization tool (v0.7.5). For MS on GRHL2 immunoprecipitants, cells were first treated with 1 µM QC6352 for 5 days, followed by the standard immunoprecipitation procedure. Proteins were separated using SDS–PAGE followed by Coomassie blue staining. Standard in-gel digestion protocol was performed on the GRHL2 SDS–PAGE gel band. Briefly, gel pieces were destained using 50% acetonitrile/H_2_O and 50 mM ammonium bicarbonate solution on a shaker (400 r.p.m.) for 1.5 h at room temperature. Subsequently, proteins were reduced and alkylated with 10 mM dithiothreitol at 37 °C and 50 mM iodoacetamide at room temperature, respectively, for 45 min at 400 r.p.m. Gel pieces were washed once with 50% acetonitrile/H_2_O and twice with 50 mM ammonium bicarbonate solution for 15 min each at 400 r.p.m. and then dehydrated with 100% acetonitrile for 10 min. Sample was digested overnight with 50 ng trypsin at 600 r.p.m. at 37 °C, and the supernatant was lyophilized before reverse-phase C18 StageTip desalting following standard protocol. Peptides were lyophilized and reconstituted in 5 µl 3% acetonitrile/5% formic acid, and 4 µl were subjected to nano liquid chromatography–tandem mass spectrometry. Peptides were separated on a self-packed C18 column with a 60-min gradient (Vanquish Neo UHPLC; Thermo Fisher Scientific) and analyzed using a standard DDA on an Exploris 480 (Thermo Fisher Scientific). The RAW file was searched in Spectrum Mill (Rev BI.08.02.218) against a SwissProt database, using cysteine carbamidomethylation as a fixed modification and lysine methylation as a variable modification. Spectra annotation was generated by Interactive Peptide Spectral Annotator^74^.

### ChIP–seq

ChIP–seq was performed as previously described in ref. ^75^. Further details are given in Supplementary Note.

### qPLEX-RIME

qPLEX-RIME was performed essentially as described in ref. ^76^, except we only used formaldehyde for cross-linking. Further details are given in Supplementary Note.

### RNA-seq

RNA-seq libraries were prepared using the Illumina TruSeq Stranded mRNA sample preparation kit from 500 ng of purified total RNA according to the manufacturer’s protocol (see details in Supplementary Note).

### CTSL activity and ROS assays

CTSL activity was detected using the Magic Red Cathepsin L Assay Kit (MyBiosource), and ROS levels were assessed using the CellROX Green and CellROX Orange reagents (Thermo Fisher Scientific) following the manufacturer’s protocol. Briefly, 2 × 10^5^ cells were seeded in 6-well plates and treated with DMSO, 1 μg ml^−1^ Dox, 10 μm ML324 or 1 μm QC6352 for the indicated time. For live cell imaging, adherent living cells were directly stained with 1× Magic Red CTSL substrates or 1× CellROX Green reagent for 30 min at 37 °C. Cells were washed 3× with PBS and stained with Hoechst 33342 for 5 min, and the images were acquired using a Nikon Eclipse microscope using ×20 magnification. ROS and CTSL magic red intensity were quantified per cell using ImageJ (v1.53q) for statistical comparison. For flow cytometry, cells were first digested and then stained with 200 μl PBS solution containing 1× magic red and 1× CellROX green for 30 min at 37 °C. Stained cells were dissociated into single cells and resuspended in 300 μl PBS and analyzed on the BD LSRFortessa Cell Analyzer with FITC and PE-Texas Red channels. An unstained sample was used as negative control. FCS files derived from BD FACSDiva (v9.0) were further analyzed using FlowJo (v10.10), and the geometric mean of each sample was calculated for statistical comparison. Gating strategy is included as Supplementary Fig. 1.

### Seahorse mito-stress assay

Seahorse mito-stress assay was performed using the Seahorse XF Cell Mito-Stress Test Kit (Agilent) following the manufacturer’s protocol (details are described in Supplementary Note).

### GSH-Glo and GSH/GSSG assay

GSH and GSSG quantification was performed using the GSH-Glo GSH Assay Kit or GSH/GSSG-Glo Assay Kit (Promega) following the manufacturer’s protocol (details are described in Supplementary Note).

### Cellular viability and colony growth assays

Cell numbers were determined by the FluoReporter Blue Fluorometric dsDNA Quantitation Kit (Thermo Fisher Scientific). For drug dose response assays, 4,000 cells were seeded into a 96-well plate per well on day 0, cell numbers were quantified on day 5 after treatment and cell growth rates at different doses were normalized to the mean of vehicle values. Nonlinear fit of dose response curves was conducted by PRISM, and area under the curve (AUC) values were derived. For combination treatment, cells were pretreated with corresponding doses of ML324 and QC6352 for 3 days in 10 cm dishes and then replated into a 96-well plate with 4,000 cells per well. A combination of BSO, cisplatin and ML324/QC6352 was added after 24 h with 6 biological replicates, and cell numbers were quantified after 5 days. The expected drug combination responses were calculated using the ZIP reference model in SynergyFinder^77^. Deviations between observed and expected responses indicate synergy for positive values and antagonism for negative values. For colony growth assays, cells were seeded into 6-well plates with 5,000 cells per well in triplicate and treated with compounds 24 h after plating. For siRNA knockdown experiments, reverse transfection was performed on day 0 with 31.25 nmol siRNAs per well using Lipofectamine RNAiMAX Reagent. siRNA transfection was refreshed on day 6. Cells were quantified between 12 and 18 days in different experiments. Briefly, cells were fixed in ice-cold methanol (Thermo Fisher Scientific) for 10 min and then stained with crystal violet staining solution (0.5%) at room temperature for 15 min. Images of each well were taken after washing three times with ddH_2_O. For quantification, crystal violet was dissolved in 10% SDS with a 500 μl volume for each well. Optical density at 450 nm value of 100 μl of destained crystal violet solution from each assay well was measured using a microplate reader with three technical replicates.

### Immunofluorescence analyses

Immunofluorescence staining was performed on cells growing in 48-well plates on glass coverslips. In total, 10,000 cells were seeded into each well. Inducible SUM149 sh*KDM4C*-infected cells were treated with control (no Dox, DMSO), sh*KDM4C* induction (plus 1 μg ml^−1^ Dox), 10 μm ML324 (no Dox), 1 μm QC6352 (no Dox) for 5 days or 1 μm Leu–Leu methyl ester hydrobromide for 24 h. Cells were then washed, fixed in 4% paraformaldehyde and blocked in 5% BSA/0.3% Triton X-100/PBS solution for 1 h. Primary antibodies against CTSL (Novus Biologicals, AF952; 1:100), GRHL2 (Sigma-Aldrich, HPA004820; 1:100) and KDM4C (Novus Biologicals, NBP1-49600; 1:100) were applied for 1 h at room temperature. Cells were washed, incubated with secondary antibodies for 30 min at room temperature and mounted using VECTASHIELD Antifade Mounting Medium with DAPI (Vector Laboratories). Cells were imaged using a Zeiss 980 Confocal Imaging System at ×63 magnification. In total, 10–13 Z-stack images were taken and merged for final visualization. Immunofluorescence staining on formalin-fixed paraffin-embedded tissue sections was performed essentially as described^78^. Briefly, FFPE sections were deparaffinized and rehydrated, followed by antigen retrieval in citrate buffer (pH 6, DAKO) for 30 min in a steamer. Endogenous peroxidase was quenched by a 10-min incubation in 3% H_2_O_2_. Blocking solution (0.3% Triton X and 5% goat serum in PBS) was applied for 1 h. Primary antibody was applied at a 1:100 dilution in blocking buffer overnight at 4 °C in a moist chamber. Secondary antibody was applied for 30 min at room temperature. Slides were mounted with Vibrance Antifade Mounting Medium with DAPI (VECTASHIELD). Images were acquired using a Nikon Eclipse microscope.

### Hi-ChIP

In situ long-range DNA-protein contact libraries were essentially generated as published^79^ with minor modifications described in Supplementary Note.

### ATAC–seq

Inducible *shKDM4C*-infected SUM149, HCC1954 and HCC1806 models were plated in 15 cm dishes and treated for 5 days under the following conditions: control (0.1% DMSO, no Dox), 1 μg ml^−1^ Dox or 10 μM ML324 (no Dox). One sample from each shKDM4C 17 and 20 model was used for SUM149 and HCC1954 cells, and biological duplicates from shKDM4C 5 were used for HCC1806. Fifty thousand cells were resuspended in 1 ml of cold ATAC–seq resuspension buffer (RSB; 10 mM Tris–HCl (pH 7.4), 10 mM NaCl and 3 mM MgCl_2_). Cells were centrifuged at maximum speed for 10 min in a prechilled (4 °C) fixed-angle centrifuge. Supernatant was carefully aspirated, and cell pellets were resuspended in 50 μl of ATAC–seq RSB containing 0.1% NP40, 0.1% Tween 20 and 0.01% digitonin by pipetting up and down 3 times and incubated on ice for 3 min. After lysis, 1 ml of ATAC–seq RSB containing 0.1% Tween 20 was added, and the tubes were inverted to mix. Nuclei were centrifuged for 5 min at maximum speed in a prechilled fixed-angle centrifuge. Supernatant was removed, and nuclei were resuspended in 50 μl of transposition mix (25 μl (2×) TD buffer, 2.5 μl transposase (100 nM final), 16.5 μl PBS, 0.5 μl 1% digitonin, 0.5 μl (10%) Tween 20 and 5 μl water) by pipetting up and down 6 times. Transposition reactions were incubated at 37 °C for 30 min in a thermomixer with shaking at 1,000 r.p.m. Reactions were cleaned up with Qiagen MinElute columns. Libraries were amplified as previously described^80^. The 35-bp paired-end reads were sequenced on a NextSeq500 instrument (Illumina).

### PRISM screen

PRISM screen was performed as previously described^26^. Further details are given in Supplementary Note.

### RNA-seq data analysis

RNA-seq data were processed using the VIPER pipeline^81^. Further details are given in Supplementary Note.

### ChIP–seq and ATAC–seq data analyses

ChIP–seq and ATAC–seq data processing were based on the ChIPs pipeline^82^. Further details are given in Supplementary Note.

### Hi-ChIP analysis

CTSL Hi-ChIP data were processed using the HiC-Pro pipeline (v3.1.0)^83^. Briefly, reads were aligned to the hg19 reference genome using Bowtie2 (ref. ^84^). After restriction site detection, a second round of alignment was performed. Validated pairs were filtered and distributed to the whole genome, binned in 500 kb resolution. Interchromosomal and intrachromosomal interactions were visualized using the HiTC package (v1.38.0)^85^ after ICE normalization. Differential interchromosomal and intrachromosomal interaction sites were called using the HiCCompare package (v1.16.0)^86^ using the cutoff of interaction frequency >50 and adjusted *P* < 0.05.

### Statistics and reproducibility

All quantitative data are presented as the mean values ± s.d. with indicated replicates in the corresponding legends including Figs. 2c,g,i, 4l, 5d,e,g,h,j and 6b,e–g,j,k, Extended Data Figs. 2e,f, 3a–e,h,k,l,n, 4l, 5g,m,o, 8e,f,j and 9b,e,g,i,k, and Supplementary Fig. 3j–n. All box plots span the upper quartile (upper limit), median (center) and lower quartile (lower limit). Whiskers extend a maximum of 1.5× interquartile range (IQR). No statistical methods were used to predetermine the sample size for the experiments. Sequencing data that did not pass quality control were excluded from analysis. In all in vivo experiments, mice were randomized to treatment groups; otherwise, experiments were not randomized. Sequencing data processing was performed by bioinformaticians blinded to the identity of the samples. The investigators were not blinded to allocation during other experiments and outcome assessment. Figure 6l was created with BioRender.com. All figures were assembled with Affinity Designer 2.0. Bioinformatic data were analyzed and visualized using R (v.4.3.1) software. Experimental data were analyzed and visualized using GraphPad Prism (v.10.3.1) software. Statistic test in R uses a precision floating-point format, which has a lower limit of approximately 2.2 × 10^−16^, and the smallest allowable reported *P* value in GraphPad PRISM is 1 × 10^−15^. When *P* values fall below these thresholds, the tools report a range (that is, *P* < 2.2 × 10^−16^ in R and *P* < 1 × 10^−15^ in GraphPad) rather than attempt to report a less precise or unreliable value.

### Reporting summary

Further information on research design is available in the Nature Portfolio Reporting Summary linked to this article.

## Online content

Any methods, additional references, Nature Portfolio reporting summaries, source data, extended data, supplementary information, acknowledgements, peer review information; details of author contributions and competing interests; and statements of data and code availability are available at 10.1038/s41588-025-02197-z.

## Supplementary information

Supplementary Information Supplementary Note and Figs. 1–3.

Reporting Summary

Supplementary Tables 1–9 Supplementary Tables 1–9.

Supplementary Data 1 Supporting data for Supplementary Fig. 2.

Supplementary Data 2 Supporting data for Supplementary Fig. 3.

## Source data

Source Data Fig. 1 Statistical source data.

Source Data Fig. 2 Statistical source data.

Source Data Fig. 4 Statistical source data.

Source Data Fig. 5 Statistical source data.

Source Data Fig. 6 Statistical source data.

Source Data Fig. 2 Unprocessed western blots.

Source Data Fig. 3 Unprocessed western blots.

Source Data Fig. 4 Unprocessed western blots.

Source Data Fig. 5 Unprocessed western blots.

Source Data Fig. 6 Unprocessed western blots.

Source Data Extended Data Fig. 1 Statistical source data.

Source Data Extended Data Fig. 2 Statistical source data.

Source Data Extended Data Fig. 3 Statistical source data.

Source Data Extended Data Fig. 4 Statistical source data.

Source Data Extended Data Fig. 5 Statistical source data.

Source Data Extended Data Fig. 7 Statistical source data.

Source Data Extended Data Fig. 8 Statistical source data.

Source Data Extended Data Fig. 9 Statistical source data.

Source Data Extended Data Fig. 10 Statistical source data.

Source Data Extended Data Fig. 1 Unprocessed western blots.

Source Data Extended Data Fig. 2 Unprocessed western blots.

Source Data Extended Data Fig. 3 Unprocessed western blots.

Source Data Extended Data Fig. 4 Unprocessed western blots.

Source Data Extended Data Fig. 5 Unprocessed western blots.

Source Data Extended Data Fig. 6 Unprocessed western blots.

Source Data Extended Data Fig. 7 Unprocessed western blots.

Source Data Extended Data Fig. 9 Unprocessed western blots.

Source Data Extended Data Fig. 10 Unprocessed western blots.

## Data Availability

All data needed to evaluate the conclusions in the paper are present in the paper and/or Supplemental Information. All raw and processed genomic data were deposited in the Gene Expression Omnibus (GEO) under accession GSE199913. All the genomic data were aligned to the human reference genome GRCh37/hg19 (https://www.ncbi.nlm.nih.gov/datasets/genome/GCF_000001405.13/). RIME data are available on ProteomeXchange with identifier PXD031768 (https://www.ebi.ac.uk/pride/archive/projects/PXD031768). The original mass spectra, spectral library and the protein sequence database used for searches have been deposited in the public proteomics repository MassIVE (identifier MSV000096930; http://massive.ucsd.edu) and are accessible at ftp://massive.ucsd.edu/v09/MSV000096930/. The mRNA expression data and the clinical data of TCGA and METABRIC were downloaded from the TCGA data portal (https://portal.gdc.cancer.gov) and Synapse (Syn1688369), respectively. For TCGA, RNA-seq reads were reprocessed using Salmon (v0.14.1)^87^ and log_2_(transcripts per million + 1) values were used. For genes with multiple probes in METABRIC, probes with the highest IQR were selected to represent the gene. Copy number information of *KDM4C* from TCGA and METABRIC was downloaded from cBioPortal and predicted by the GISTIC algorithm^88^. Gene copy number, subtype and RNA-seq (fragments per kilobase of transcript per million mapped read) for 57 breast cancer cell lines were downloaded from Cancer Cell Line Encyclopedia^89^; copy number gain or deletion was defined as log_2_ copy number above or below 1.2. AUCs for different chemotherapy drugs were downloaded from DepMap (https://depmap.org/portal/)^51^. Microarray data from five neoadjuvant therapy TNBC cohorts were downloaded from GEO with the following accessions: GSE32646, GSE32603, GSE20194, GSE25066 and GSE18864. log_2_-Normalized probe intensities were used for signature enrichment analysis. The mRNA and normalized metabolomic profile data from the Fudan University Shanghai Cancer Center (FUSCC) cohort were downloaded from ref. ^43^ and GEO with the accession code GSE118527. For KDM4C ChIP–seq peak overlap analysis, publicly available H3K27ac ChIP–seq data were downloaded from GEO with the accession codes GSE72956 (HCC1954), GSE57436 (MCF7) and GSE65201 (T47D). H3K4me3 ChIP–seq data were downloaded from GSE54693 (MCF7) and GSE80592 (T47D). Source data are provided with this paper.

## Code availability

This study does not report original code. All data were analyzed and processed using published software packages, the details of which are provided and cited in Methods.
