# Supplementary material for: KDM4C inhibition blocks tumor growth in basal breast cancer by promoting cathepsin L-mediated histone H3 cleavage
Source: Nat Genet. 2025 Jun 2;57(6):1463–77. doi: 10.1038/s41588-025-02197-z (PMC12165855; doi:10.1038/s41588-025-02197-z)
Supplement: Supplementary file 1 — Supplementary Note and Figs. 1–3. [file 41588_2025_2197_MOESM1_ESM.pdf]

nature genetics

Supplementary information https://doi.org/10.1038/s41588-025-02197-z

KDM4C inhibition blocks tumor growth in basal breast cancer by promoting cathepsin L-mediated histone H3 cleavage

In the format provided by the authors and unedited

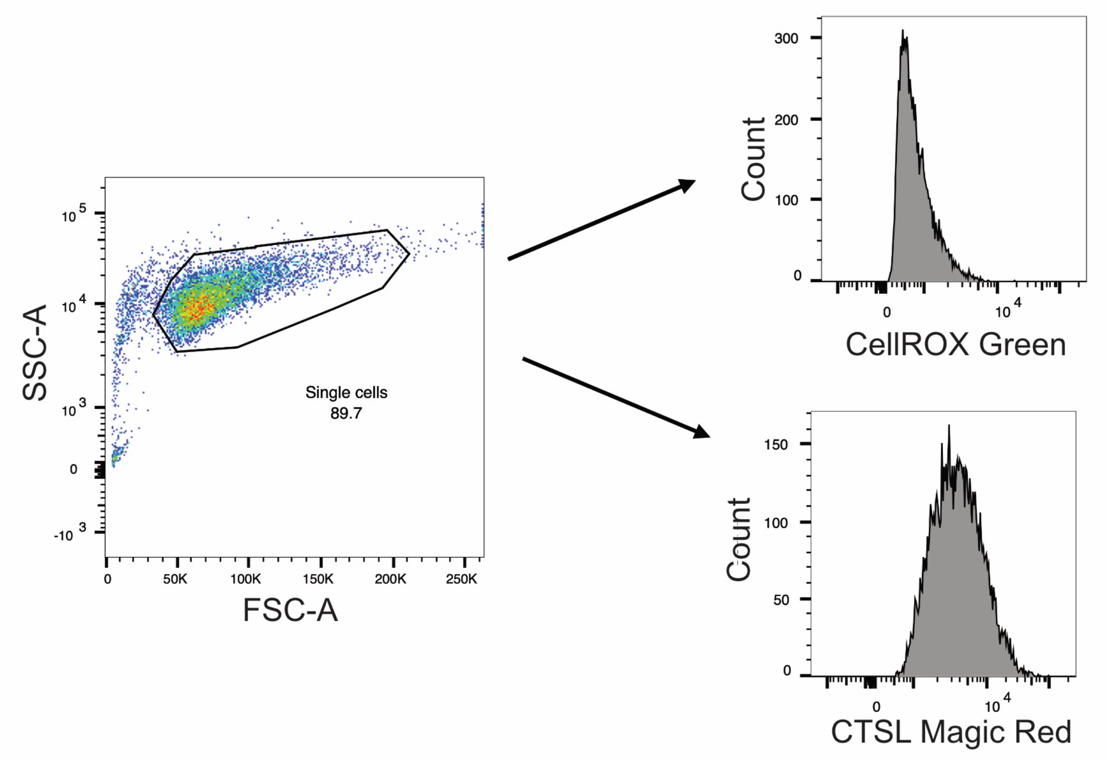

Supplementary Figure 1. Flow cytometry gating strategy for ROS and CTSL activity. Gating strategy for single cell selection for flow cytometry measurement in Fig. 2h, 4k, 5f, 6i, and Extended Data Fig. 5j, 5l, 9a, 9m.

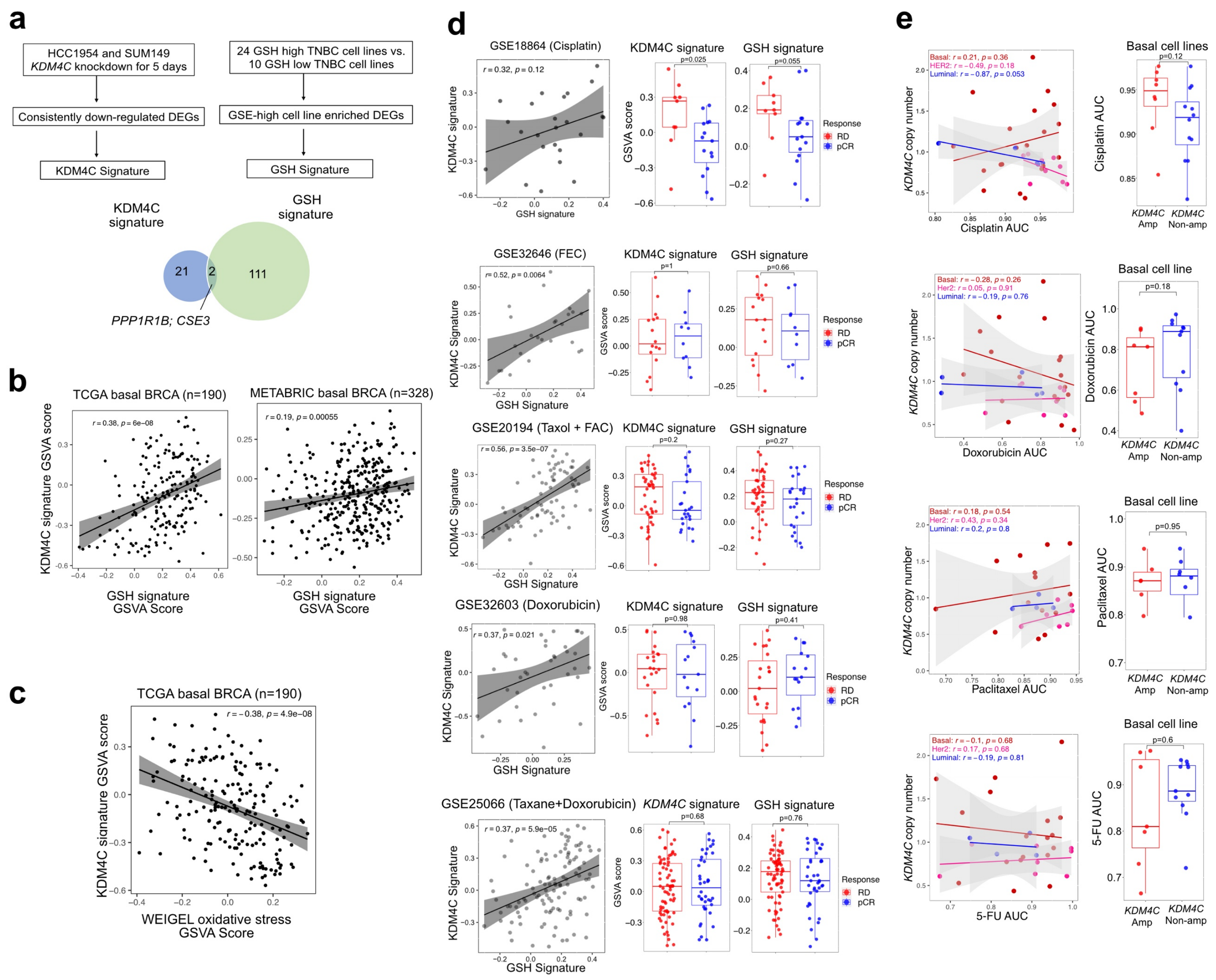

Supplementary Figure 2. Clinical relevance of the KDM4C-CTSL-GSH axis. a, Flowchart of KDM4C and GSH gene signatures generation in TNBC. Venn diagram below represent the intersection of genes in the two signatures. **b,** Scatter plot depicting correlation between KDM4C and GSH signatures in basal primary tumors from the TCGA and METABRIC cohorts. Number of cases used is indicated for each cohort. **c,** Scatter plot depicting the correlation between GSH and oxidative stress signature enrichments in basal tumors the TCGA cohort. **d**, Scatter plots illustrate correlations between KDM4C and GSH signatures in all TNBC primary tumors from five neoadjuvant trials. R and p values were dervied from Pearson correlation. Box plots show enrichment levels of KDM4C and GSH signature in pre-treatment tumors with pathological responses (pCR) or residual disease (RD). Box plots span the upper quartile (upper limit), median (center) and lower quartile (lower limit). Whiskers extend a maximum of 1.5X IQR. Mann Whitney U test (two-sided) was used for each signature. FEC stands for combination of fluorouracil, epirubicin hydrochloride, and cyclophosphamide; FAC stands for combination of fluorouracil, adriamycin and cytoxan. **e**, Scatter plots show correlation of *KDM4C* copy numbers and cisplatin, doxorubicin, paclitaxel, and 5-FU AUCs obtained from DepMap portal in breast cancer cell lines. Correlations were conducted separately for cell lines of different subtypes (n=20 basal, n=9 HER2+ and n=5 luminal). Box plots depict AUCs of the indicated drugs in *KDM4C* amplified (n=8) and non-amplified (n=12) basal breast cancer cell lines. Box plots span the upper quartile (upper limit), median (center) and lower quartile (lower limit). Whiskers extend a maximum of 1.5X IQR. Mann Whitney U test (two-sided) was used. The linear regression lines with 95% confidence interval are shown for the correlation plots in panel b,c,d,e.

a

Cell line	*KDM4C* Amp	*BRCA1* Mut	Lineage	GSH subtype
SUM149	Yes	Yes	Basal	High
HCC38	Yes	No	Basal	High
HCC3153	No	Yes	Basal	High
HCC1954	Yes	No	Basal/HER2+	High
HCC70	Yes	No	Basal	Low
MDA-MB-436	No	Yes	Mesenchymal	Low

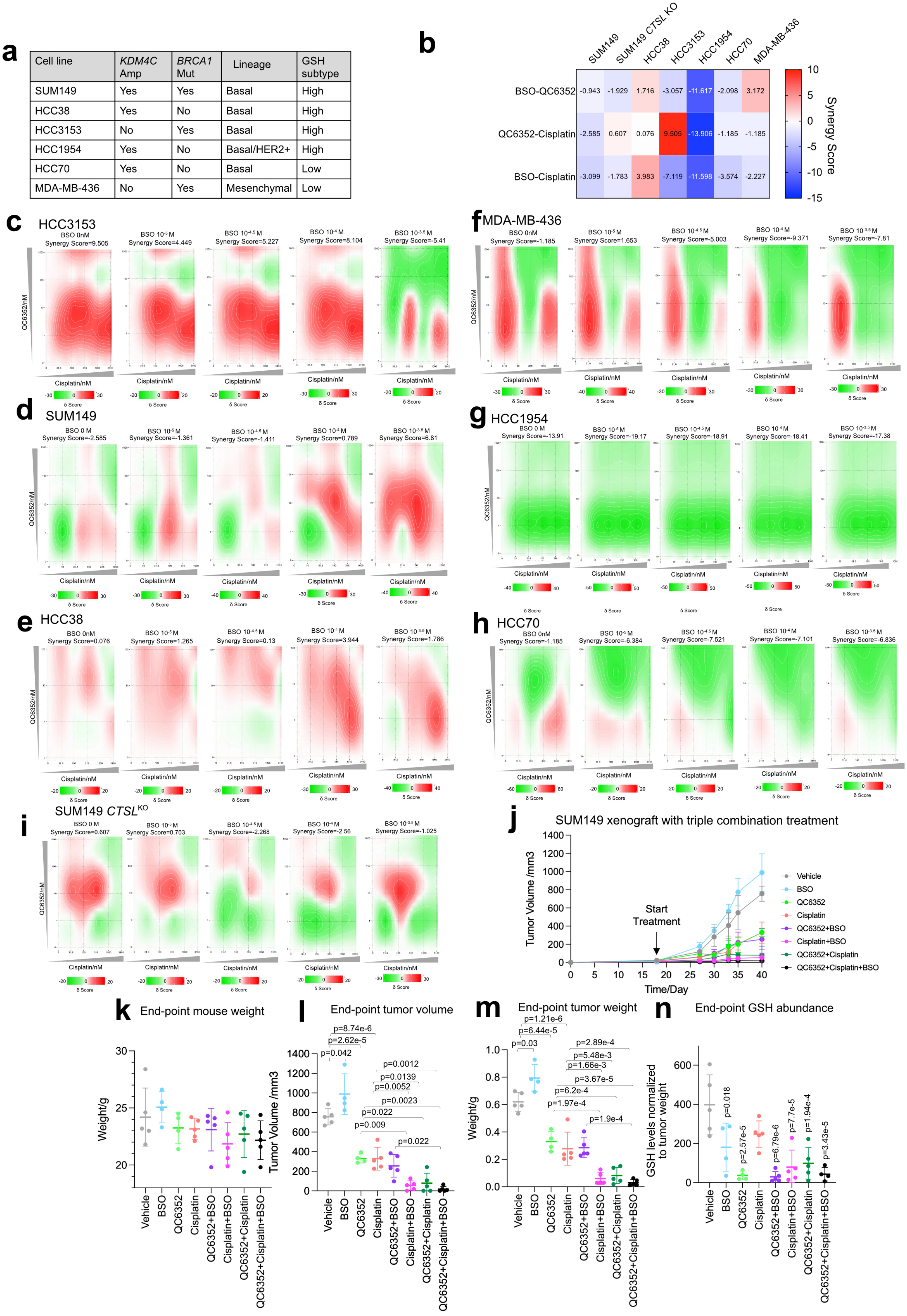

Supplementary Figure 3. KDM4C-GSH nexus reveals a therapeutic vulnerability to overcome cisplatin resistance. a, Table summarizing the genetic characteristics of the six cell lines used for compound testing. **b**, Heatmap depicting Bliss synergy scores of each two agent across all the cell models tested. **c-i**, Synergy plots for combination of cisplatin and QC6352 with increasing doses of BSO in the indicated cell lines. Synergy score was calculated using Bliss model where a score of 0 indicates an additive response and areas of red and green indicate synergistic and antagonistic dose regions, respectively. **j**, Plot depicting growth of SUM149 xenografts in mice treated with the indicated compounds. Data are presented as mean ± SD with n=4 for BSO and QC6352 and n=5 for the remaining groups. **k-n**, Dot plots depicting the mean ± SD of endpoint mouse weight (**k**), tumor volumes (**l**), tumor weight (**m**) and intratumor GSH levels (**n**) of SUM149 xenografts from mice treated with the indicated agents for 22 days (n=4 tumors for BSO, QC6352 and triple combination groups or n=5 tumors for the rest groups as biological replicates). Ordinary one-way ANOVA (two-sided) was used for each comparison. P-values in panel n indicate comparisons between vehicle and each group.

SUPPLEMENTARY NOTE

Cell line models

Dox-inducible sh*KDM4C* expressing derivates of cell lines were generated by infection with lentivirus. Briefly, 12 µg of the corresponding lentiviral vector, 8 µg of pCMV-dR8.91 and 4 µg of pCMV-VSV-G were mixed with 60 µl of Lipofectamine 2000 in 1ml Optimem. Collagen I precoated T75 flasks were seeded with $1x10^7$ 293FT cells and kept upright while adding the transfection mix. Flask contents were pipetted to homogenize, and cells were allowed to attach overnight at standard culture conditions. Media was then changed with 12 ml of pre-warmed fresh media. After a 48h incubation, viral supernatant was collected, and fresh media added for two more collections after 12h and 24h. Viral supernatants were kept at 4ºC, combined, and spun at 500xg for 10min at 4ºC. Supernatants were then filtered through a 0.45 µm PVDF syringe filter, mix with one third volume of Lenti-X-Concentrator (Clontech) and precipitated overnight at 4ºC. After a centrifugation at 1500xg for 45min at 4ºC, supernatants were discarded, and viral pellets were resuspended 350 µl media. Exponentially growing target cells were infected twice on consecutive days with 120 µl of concentrated virus in 6-well plates. Cells were grown in fresh media and passaged to T24 flasks or $15cm^2$ dish and allowed to attach overnight. Selection was then started with 5µg/ml puromycin*.* sh*KDM4A* and sh*KDM4B* derivatives*,* $KDM4C^{WT}$, $KDM4C^{S198M}$, $KDM4C^{\Delta TTD}$ ectopic overexpressing cell models, $CTSL^{KO}$, $GRHL2^{KO}$, V5 or GFP tagged-H3 expressing cell lines were generated essentially the same way and selected in 2 µg/ml puromycin (sh*KDM4A/B* SUM149 derivatives), 500 µg/ml geneticin ($KDM4C^{WT}$ and $KDM4C^{S198M}$ ectopic overexpressing SUM149 cells), 5 µg/ml blasticidin ($GRHL2^{KO}$, V5-H3.1, GFP-H3.1 and $KDM4C^{\Delta TTD}$ ectopic overexpressing model) or 100 µg/ml hygromycin ($CTSL^{KO}$). All the constructs were established via commercial vendor. For $GRHL2^{K94R}$,$GRHL2^{K453R}$,$GRHL2^{K94R/K453R}$ constructs, site-specific mutagenesis were carried out based on the $GRHL2^{WT}$ construct and introduction of mutants was confirmed using Sanger sequencing. Experiments with GRHL2 lentiviral infection were conducted in a transient manner (i.e. no antibiotic selection) since GRHL2 was not able to be stably expressed in SUM149 cells. Detailed construct and sequence information are in the **Supplementary Table 9**.

Animal experiments

For xenograft assays with doxycyclin-induced shRNA knockdown, 1 X 10^6 SUM149, HCC1954 or HCC1806 cells expressing TET-inducible *KDM4C*-targeting shRNAs were resuspended in 50% Matrigel/50% serum-free medium (BD Biosciences) and injected orthotopically into the mammary fat pads of 6-week-old female NCr nude (HCC1954 and SUM149) or NSG (HCC1806) mice. When the tumors became palpable, shRNAs were induced by administering doxycycline diet with 625 ppm or started ML324 treatment (100 mg/kg/day using an Alzet osmotic pump).

For xenograft assays using QC6352 or cisplatin/QC6352/BSO drug combination, SUM149 or HCC1806 *CTSL*^{KO} , and HCI-041 PDX, 1 X 10^6 cells (SUM149 parental, sgScramble and *CTSL*^{KO} derivatives or dissociated PDX tumor single cell suspension) in 50 µl of 1:1 mix of Matrigel and serum-free DMEM/F12 medium were injected orthotopically into the inguinal mammary fat pad of 6-week-old female NCr nude (cisplatin/QC6352/BSO drug combination experiment) or NSG (HCI-041 PDX and SUM149/HCC1806 *CTSL*^{KO} experiment) mice. Mice were randomized after all mice developed palpable tumors to ensure no pre-treatment tumor volume differences. Compounds were administered as below: QC6532 (5mg/kg for triple combination experiment and 10mg/kg for the rest) and ML324 (50mg/kg) were given by daily oral gavage. Both compounds were dissolved in 90% corn oil + 10% DMSO. Cisplatin (3mg/kg) and BSO (125mg/kg) were given by intraperitoneal injection twice a week and daily respectively. Animals were euthanized, and tumors were harvested when tumors in the control group reached ~ 1.5 cm^3 size. Animals were euthanized if experimental or humane (e.g., 2 cm maximum tumor size in any dimension) endpoints were reached as defined in Dana-Farber Cancer Institute Animal Care and Use Committee approved protocol 11-023. Tumor volumes were calculated using the formula (length X $width^2$)/2. For histological analyses, 5µm sections of formalin-fixed paraffin embedded (FFPE) tissue slides were stained with hematoxylin and eosin using standard protocols. *KDM4C*-amplified TNBC PDX models (i.e., HCI-041, HCI-045 and HCI-050) generously provided by Dr. Alana L. Welm were previously

reported[1]. PDX in tumor chunk format was first implanted in NSG mice for one round of expansion and the second passage of PDX tumor were dissociated into single cell suspension and mixed for the experiment. The amplification of KDM4C and 9p24 locus of HCI041 model has been previously confirmed by a SNP array[1] and whole exome sequencing[2].

Immunoblot

Cells were lysed in RIPA buffer containing Halt protease and phosphatase inhibitor Cocktail (Life Technologies) and subjected to cup horn sonication in 70% amp for 5 minutes with a cycle of 20s on and 10s off. Lysates mixed with sample buffer were resolved in 4%-12% Bis-Tris gels, 10% Tricine or 3-8% Tris-Acetate gels (Life Technologies) and transferred to PVDF membrane with 80 V voltage in four degree for 2 hours. For cell fractionation assays, cell nuclei, cytoplasm, and membrane fractions were purified using Cell Fractionation Kit (Cell Signaling Technology #9038). Membranes were blocked with 5% milk in in PBS-T (0.1% Tween20 in PBS) for 1 hour at room temperature and then incubated with primary antibodies at 1:500-1:5,000 dilution in 5% milk PBS-T overnight at 4^oC. After 3×5-minute washes, membranes were incubated with secondary antibodies at 1:2,000 dilution, then washed 2× 5 minutes followed by a 15-minute wash. The membranes were then developed using clarity or clarity max ECL western blotting substrate (Bio-Rad) and bands visualized using Bio-Rad Chemidoc Imaging System with developing time varying between 1 second to 10 minutes. The specificity of antibodies against KDM4C, CTSL, and GRHL2 were validated using genetic knockout or knockdown models. Antibodies against C and N terminal histone H3 (Abcam) were all polyclonal where synthetic peptide corresponding to human histone H3 AA100 to C-terminus and AA1-100 were used as original immunogen. Antibody detecting GCLC protein was first validated in a TNBC panel of 22 cell lines with known GCLC RNA levels by confirming significant positive correlation between quantified protein expression and mRNA levels. Detailed antibody and their usage information are in the **Supplementary Table 9**.

Chromatin immunoprecipitation sequencing (ChIP-seq)

ChIP-seq was performed as previously described[3]. For histone modification ChIP-seq, cells grown in 150 mm tissue culture plates were fixed with 1% formaldehyde (Electron Microscopy Sciences, 15714) in fixing buffer (50 mM HEPES-KOH, pH 7.5, 100 mM NaCl, 1 mM EDTA, pH 8, and 0.5 mM EGTA, pH 8) for 10 minutes at 37°C. For CTSL and GRHL2 ChIP-seq, cells were pre-fixed with 15 mM ethylene glycol bis(succinimidyl succinate) (EGS) diluted 1:10 in PBS for 30 minutes at room temperature (RT), followed by 1% formaldehyde for the last 10 minutes. Crosslinking was quenched by adding glycine to a final concentration of 0.125 M and rocking the plates for 5 minutes. Cell monolayers were washed twice with ice-cold PBS, scraped into 10 ml PBS, centrifuged at 200g for 5 minutes, and flash-frozen after removing the supernatant. Frozen pellets were resuspended in 1 ml of Cell Lysis Buffer (50 mM HEPES, pH 8, 140 mM NaCl, 1 mM EDTA, pH 8, 10% glycerol, 0.5% NP-40, 0.25% Triton X-100, and protease/phosphatase inhibitors [PPI]) and incubated for 10 minutes at 4°C with gentle rotation. Nuclei were pelleted by centrifugation at 1,700g for 5 minutes at 4°C, washed twice with 1 ml Wash Buffer (10 mM Tris-HCl, pH 8, 200 mM NaCl, 1 mM EDTA, pH 8, 0.5 mM EGTA, pH 8, and PPI), and resuspended in 1 ml Shearing Buffer (10 mM Tris-HCl, pH 7.4, 1 mM EDTA, pH 8, 0.1% SDS, 1% Triton X-100, 0.1% sodium deoxycholate, 0.25% N-lauroylsarcosine, 1 mM DTT, and PPI). Chromatin was fragmented using a Covaris E220 focused ultrasonicator (peak intensity: 140, 5% duty cycle, 200 cycles per burst, 10–12 minutes) in 1 ml AFA fiber tubes, with 5×10^6 cells sonicated per tube for each ChIP. Lysates were centrifuged at 10,000g for 15 minutes at 4°C, and the cleared supernatants were transferred to new tubes. NaCl was added to a final concentration of 150 mM, and samples were pre-cleared with 40 µl/ml Dynabeads Protein G (Life Technologies, 10003D) for 1 hour at 4°C with constant rotation. After magnetic separation, the appropriate primary antibody was added, and immunoprecipitation was performed overnight at 4°C with gentle rotation. Antibody-bound chromatin was further precipitated with pre-washed Dynabeads Protein G for 2 hours at 4°C with constant rotation. Beads were washed sequentially with low-salt wash buffer (20 mM Tris-HCl, pH 8, 150 mM NaCl, 10 mM EDTA, 1% SDS),

high-salt wash buffer (50 mM Tris-HCl, pH 8, 10 mM EDTA, 1% SDS), LiCl wash buffer (50 mM Tris-HCl, pH 8, 10 mM EDTA, 1% SDS), and twice with 1× TE buffer. Each wash was performed for 5 minutes at 4°C with gentle rotation. DNA was eluted with 300 µl of 100 mM sodium bicarbonate and 1% SDS for 30 minutes at RT with constant shaking. Crosslinks were reversed overnight at 65°C, followed by sequential RNA and protein digestion using 0.2 mg/ml RNase A for 30 minutes at 37°C and 0.2 mg/ml Proteinase K for 1 hour at 55°C. DNA was extracted using 300 µl phenol/chloroform/isoamyl alcohol (Calbiochem, #516726), centrifuged at 20,000g for 5 minutes at RT, and precipitated with isopropanol, 1/3 volume of 2 M sodium perchlorate, and 5 µl glycogen. After centrifugation at 20,000g for 10 minutes, the DNA pellet was washed twice with 70% ethanol and resuspended in 20 µl low TE buffer. ChIP-seq libraries were prepared using the ThruPLEX DNA-seq Kit (Rubicon Genomics, R400427) following the manufacturer's protocol. Library fragments were size selected by PAGE between 150 and 700 kb. Seventy-five base pair single-end reads were sequenced on a NextSeq500 instrument (Illumina). Antibody details are provided in **Supplementary Table 9**.

qPLEX-RIME

qPLEX-RIME was performed essentially as described[4] except we only used formaldehyde for crosslinking. Briefly, the media of exponentially growing cells (~1x10^8 cells, several 150 mm plates were needed) were removed and replaced by 1% formaldehyde-containing complete fresh media. Cross-linking was carried on at room temperature for 8 minutes with gentle shaking. Cross-linking reactions were quenched by adding glycine to a final concentration of 125 mM and incubated a room temperature for 5 minutes with gentle rocking. Cells were then washed with 10 ml ice-cold PBS/plate and scrapped off the plate into 500 µl ice-cold PBS with protease and phosphatase inhibitors (PPI). Cell pellets were washed with PBS and nuclear fractions were extracted by first resuspending the pellet in 10 ml lysis buffer 1 (50 mM HEPES-KOH pH7.5, 140 mM NaCl, 1 mM EDTA, 10% Glycerol, 0.5% NP-40, 0.25% Triton X-100 and 1x PPI) for 10 minutes at 4°C with gentle rotation. Nuclei were then pelleted,

resuspended in 10 ml of lysis buffer 2 (10 mM Tris-HCL pH8.0, 200 mM NaCl, 1 mM EDTA, 0.5 mM EGTA and 1x PPI) and incubated at 4°C for 5 minutes with gentle rotation. Nuclei were again pelleted and resuspended in 1.5 ml of lysis buffer 3 (10 mM Tris–HCl pH8, 100 mM NaCl, 1 mM EDTA, 0.5 mM EGTA, 0.1% sodium deoxycholate, 0.5% N-lauroylsarcosine and 1x PPI) and 300 µl aliquots were sonicated in a cup horn sonicator with the same settings used for western blot samples. Then 30 µl of 10% Triton-X100 was added to each 300 µl aliquot of sonicated lysate and the lysate clarified by centrifugation at 20,000g for 10 min at 4°C. Supernatants were then incubated with 100 µl of magnetic beads pre-bound to KDM4C antibody or matching IgG control antibody and immunoprecipitation carried on overnight at 4°C with gentle rotation. Beads were washed 10 times with RIPA buffer (50 mM HEPES pH7.6, 1 mM EDTA, 0.7% sodium deoxycholate, 1% NP-40, 0.5 M LiCl and 1x PPI) and twice in 100 mM ammonium hydrogen carbonate (AMBIC) solution. For the second AMBIC wash, the beads were transferred to new tubes. Bead pellets were snap frozen in liquid nitrogen. Trypsin solution at 15 ng/µl (Pierce) was added to the suspension of the magnetic beads followed by overnight incubation at 37°C. The next day a second digestion trypsin step was performed for 4h at 37°C. The samples were placed on a magnet and the supernatant solution was collected and acidified. The peptides were cleaned with the Ultra-Micro C18 Spin Columns (Harvard Apparatus) according to manufacturer's instructions and dried with speedvac. Dry samples were reconstituted in 100 µl of 0.1M TEAB (triethylammonium bicarbonate) and labelled using the TMT-10plex reagents (Thermo Fisher). The peptide mixture was fractionated with Reversed-Phase cartridges at high pH (Pierce, #84868) and peptide fractions were analyzed on a Dionex Ultimate 3000 UHPLC system coupled with the LTQ Orbitrap Velos mass spectrometer (Thermo Scientific). Mobile phase (A) was composed of 2% acetonitrile, 0.1% formic acid, 5% dimethyl sulfoxide (DMSO) and mobile phase (B) was composed of 80% acetonitrile, 0.1% formic acid, 5% DMSO. The precursor scans were performed in the Orbitrap in the range of 380-1500 m/z at 60K resolution. The MS2 scans were performed in the ion trap with CID collision energy 30% and in the orbitrap with HCD collision energy 40% back-to-back for each precursor. The processing of the raw data was performed in Proteome Discoverer 2.1 using the SequestHT search engine. The node for

SequestHT included the following parameters: Precursor Mass Tolerance: 20ppm, Fragment Mass Tolerance: 0.5Da for the CID spectra and 0.02Da for the HCD spectra, dynamic modifications: oxidation of M (+15.995Da) and deamidation of N, Q (+0.984Da) and static modifications: TMT6plex at any N-Terminus and K (+229.163Da). The consensus workflow included S/N calculation for TMT intensities and the level of confidence for peptide identifications was estimated using the Percolator node with decoy database search. Strict FDR was set at qvalue.

Seahorse mito-stress assay

Seahorse mito-stress assay was performed using Seahorse XF Cell Mito Stress Test Kit (Agilent) following manufacturer's protocol. Briefly, 1 X 10^6 cells were seeded into six well plate and treated with for 5 days. Cells were then trypsinized and 1.5 X 10^5 cells were replated into XFe24 seahorse 24 well plates at 100% confluence. Four wells of the plate were left unseeded for calibration purposes. A hydrate cartridge was incubated in calibrating solution at 37°C overnight. Before measurement, cells were washed twice gently with Seahorse DMEM medium supplemented with 10mM pyruvate, 20mM glutamine, and 100mM glucose, and incubated at 37°C for 1 hour. Oxygen consumption rates were measured using Seahorse XFe24 Analyzer following consecutively addition of 1.5 μM oligomycin, 1 μM FCCP, and 0.5 μM Rotenon/antimycin A. Three data points were recorded for each condition. Specific mitochondrial functional annotation was carried out following the manufacturer's instructions.

PRISM Screen

Cell Lines**:** The current PRISM cell set consists of 931 cell lines representing more than 45 lineages including both adherent and suspension/hematopoietic cell lines. These cell lines largely overlap with and reflect the diversity of the Cancer Cell Line Encyclopedia (CCLE) cell lines (see https://portals.broadinstitute.org/ccle). Cell lines were grown in RPMI without phenol red + 10% FBS for adherent lines and RPMI without phenol red + 20% FBS for suspension lines. Parental cell lines were stably infected with a unique 24-nucleotide DNA barcode via lentiviral transduction and blasticidin

selection. After selection, barcoded cell lines were expanded and subjected to quality control (mycoplasma contamination test, a SNP test for confirming cell line identity, and barcode ID confirmation). Passing barcoded lines were then pooled (20-25 cell lines per pool) based on doubling time similarity and frozen in assay-ready vials.

PRISM Screening: Test compound QC6352 was added to 384-well plates at 8-point dose with 3-fold dilutions in triplicate. These assay-ready plates were then seeded with the thawed cell line pools. Adherent cell pools were plated at 1250 cells per well, while suspension and mixed adherent/suspension pools were plated at 2000 cells per well. Treated cells were incubated for 5 days then lysed. Lysate plates were collapsed together prior to barcode amplification and detection.

Barcode Amplification and Detection**:** Each cell line's unique barcode is located in the 3'UTR of the blasticidin resistance gene and therefore is expressed as mRNA. Total mRNA was captured using magnetic particles that recognize polyA sequences. Captured mRNA was reverse-transcribed into cDNA and then the sequence containing the unique PRISM barcode was amplified using PCR. Finally, Luminex beads that recognize the specific barcode sequences in the cell set were hybridized to the PCR products and detected using a Luminex scanner which reports signal as a median fluorescent intensity (MFI).

Data Processing:

I. Each detection well contained 10 control barcodes in increasing abundances as spike-in controls. For each plate, we first create a reference profile by calculating the median of the $\log_2$(MFI) values across negative control wells for each of these spiked-in barcodes.
II. For each well, a monotonic smooth p-spline was fit to map the spike in control levels to the reference profile. Next, we transform the $\log_2$(MFI) for each cell barcode using the fitted spline to allow well-to-well comparisons by correcting for amplification and detection artifacts.

III. Next, the separability between negative and positive control treatments was assessed. In particular, we calculated the error rate of the optimum simple threshold classifier between the control samples for each cell line and plate combination. Error rate is a measure of overlap of the two control sets and was defined as

$$\text{Error}=\frac{FP+FN}{n}$$

where FP is false positives, FN is false negatives, and n is the total number of controls. A threshold was set between the distributions of positive and negative control $\log_2$(MFI) values (with everything below the threshold said to be positive and above said to be negative) such that this value is minimized. Additionally, we also calculated the dynamic range of each cell line. Dynamic range was defined as

$$DR=\mu_- - \mu_+$$

where μ+/− stood for the median of the normalized logMFI values in positive/negative control samples.

IV. We filtered out cell lines with error rate above 0.05 or a dynamic range less than 1.74 from the downstream analysis. Additionally, any cell line that had less than 2 passing replicates was also omitted for the sake of reproducibility. Finally, we computed viability by normalizing with respect to the median negative control for each plate. Log-fold-change viabilities were computed as

$$\text{log-viability} = \log_2(x) - \log_2(\mu_-)$$

where $\log_2(x)$ is the corrected $\log_2$(MFI) value in the treatment and $\log_2(\mu-)$ is the median corrected $\log_2$(MFI) in the negative control wells in the same plate.

V. Log-viability scores were corrected for batch effects coming from pools and culture conditions using the ComBat algorithm as described[5].

VI. We fit a robust four-parameter logistic curve to the response of each cell line to the compound:

$$f(x) = b + \frac{a-b}{1+e^{s\log\frac{x}{EC50}}}$$

With the following restrictions:

1. We require that the upper asymptote of the curve be between 0.99 and 1.01
2. We require that the lower asymptote of the curve be between 0 and 1.01
3. We do not enforce decreasing curves
4. We initialize the curve fitting algorithm to guess an upper asymptote of 1 and a lower asymptote of 0.5
5. When the standard curve fit fails, we report the robust fits provided by the dr4pl R-package

and computed AUC values for each dose-response curve and IC_{50} values for curves that dropped below 50% viability.

VII. Finally, the replicates were collapsed to a treatment-level profile by computing the median log-viability score for each cell line.

After data processing, we calculated univariate associations between the PRISM sensitivity profiles (each dose, $\log_2$(AUC), and $\log_2$(IC_{50})) and used $\log_2$(AUC) for the downstream analysis.

RNA-seq data analysis

RNA-seq data were processed using VIPER pipeline[6]. Briefly, fastq files were aligned to the human reference GRCh37/hg19 genome using the STAR RNA-Seq aligner (version STAR_2.5.1b)[7]. Genes with 0 counts across all samples were filtered out. The gene-level counts from all studies were then

normalized using TMM with edgeR[8]. Log2 transformed TMM-normalized counts per million [log2(TMM-CPM + 1)] were used for analysis. Principle component analyses were conducted using prcomp function. Differentially expressed genes were identified using DESeq2[9] with cutoff of |FC|>2, padj<0.05. For QC6352-induced DEGs in sgScramble and CTSL KO models, a more strigent cutoff of |FC|>4, padj<0.05 was used. Heatmap of DEG union was generated by Complexheatmap package. Hallmark Signature enrichment scores were calculated using GSVA package[10]. Delta enrichment scores were computed by subtracting the mean values of the control groups from the treated groups. For integration of RNA-seq and ChIP-seq, BETA was performed as previously described[11]. Briefly, differentially expressed (DE) genes were first computed using DESeq2 and used as one of the inputs. BETA basic modules were used to compute the statistical associations between DE genes and DB peaks using 100kb as the ranges to link gene TSS to each peak. P values were derived using one-tailed Kolmogorov-Smirnov test for up-regulated and down-regulated genes respectively.

ChIP-seq and ATAC-seq data analyses

ChIP-seq and ATAC-seq data processing were based on chips pipeline[12]. Reads were aligned to hg19 genome using BWA-MEM aligner[13]. Peak calling was performed using MACS2 v2.1.2 with FDR<0.01 as the cutoff[14]. Seqplots[15] was used to make the ChIP-Seq heatmaps and intensity plots using Bigwig files. DiffBind package was used for principle component analysis and peak set interaction based on Bed file[16]. Genomic track visualization were conducted using WashU Epigenome Browser[17]. For motif enrichment analysis, fasta sequence files were extracted based on Bed files using BEDtools[18] and then input into MEME Suite AME module[19] using HOCOMOCO full v11 database[20] for calculation. E value below 10^{-5} is considered as a significant enrichment. Uniquely enriched motifs in gained ATAC peaks were derived by intersecting all the significantly enriched motifs in gained sites and non-changed sites and overlapped motifs were removed. For global signal intensity comparison for H3K9me3 and H3K36me3, signal intensities at each 5kb bin of genome were calculated using the Cobra software[21],

where multiBamSummary function was used from deepTools 3.5.0[22] and differential marked regions were derived by DESeq2 package (v1.41.1) with adjusted p value <0.05. For ATAC-seq differentially peak analysis, all ATAC peaks were first merged, signal intensities were quantified on the concated peak sets and differential peaks were calculated using DESeq2 using cutoff of adjust p value<0.05 in HCC1806 cells where one shRNA models (sh5) with biological duplicate was used. In HCC1954 and SUM149 cells, two different shRNA models were applied. Gained or lost peaks from each treatment were first obtained by intersecting with vehicle groups. Differentially gained or lost peaks were called by consensus gain/lost peaks in both sh17 and sh20 models of each cell line with |DEseq2-derivied fold change|>1.5.

GSH-Glo and GSH/GSSG assay

GSH and GSSG quantification was performed using GSH-Glo™ Glutathione assay kit or GSH/GSSG-Glo™ Assay kit (Promega) following the manufacturer's protocol. For in vitro assays, 4,000 cells/well were seeded in 96 well plates and treated with DMSO, 1 µg/ml doxycycline, 10 µM ML324, 1 µM QC6352 or 1 µM BSO. GSH or GSSG levels were quantified at day 2 by adding 1X GSH-Glo reagent (30 minutes of incubation) following 1X luciferin detection reagent (15 minutes of incubation) after medium removal. Luminescence was measured by plate reader. Readout from GSH-Glo assays was further normalized to cell numbers quantified using FluoReporter™ Blue Fluorometric dsDNA Quantitation Kit (Fisher Scientific) in a parallel 96 well plate. For tumor GSH level measurement, snap frozen tumors were first thawed on ice and a chunk of each sample (10-40 mg) was used for homogenization in PBS containing 2mM EDTA. To avoid the spatial bias (i.e., tumor core vs surface) of GSH abundance, all the selected tumor chunks contained both regions. 50 µl of each tumor lysate was used with technical duplicates for the GSH-Glo assay by adding 2X GSH-Glo reagent (30 minutes of incubation) followed by 1X luciferin detection reagent (15 minutes of incubation). Luminescence was

measured by plate reader. Readout from GSH-Glo assays was further normalized to input the tumor tissue weight.

Hi-ChIP

In situ long-range DNA-protein contact libraries were essentially generated as published[23] with minor modifications. Briefly, $10x10^6$ EGS/formaldehyde fixed SUM149 cells (as described for ChIPseq) were resuspended in 500ul HiChIP lysis buffer (10 mM Tris-HCl pH 7.5, 10 mM NaCl, 0.2% NP-40) at 4ºC for 30min, spun for 5min at 2500xg, washed with 500ul of lysis buffer and pelleted again. Pellets were resuspended in 0.5% SDS and incubated at 62ºC for 10min without shaking. Then 285ul dH2O and 50ul 10% Triton X-100 were added and samples incubated at 37ºC for 15min with rotation. Digestion was carried out by adding 50ul 10x NEB buffer 2 and 375U of MboI restriction enzyme, followed by incubation at 37ºC during 2h with rotation. Enzyme was inactivated at 62ºC for 20min without shaking. At this point incorpotation master mix (0.4mM biotin-dATP, 10mM of each dCTP, dGTP and dTTP and 5U/ul of the Klenow fragment of Pol I) was added and incubated at 37ºC for 1h with rotation. Ligation was done in 948ul of ligation master mix (150 µL 10X NEB T4 DNA ligase buffer with 10 mM ATP, 125 µL of 10% Triton X-100, 15ul 10mg/ml BSA, 10ul of 400U/ul T4 DNA ligase and 660ul dH2O) with a 4h incubation at room temperature with rotation. Nuclei were pelleted at 2500xg for 5min at 4ºC and brought to 1ml with RIPA buffer plus sarkosyl as described for ChIP. Samples were sonicated for 9min under the same Covaris settings. Antibody incubation, Protein G bead conjugation, sequential washing of beads, DNA elution, de-crosslinking and DNA purification were carried out as for ChIP-seq. Streptavidin C-1 beads, 5ul per sample, were pre-washed in Tween wash buffer (TWB: 5 mM Tris-HCl pH 7.5, 0.5 mM EDTA, 1M NaCl, 0.05% Tween-20), re-suspended in 10ul of 2x Biotin binding buffer (10 mM Tris-HCl pH 7.5, 1 mM EDTA, 2M NaCl) per 10ul sample, added to purified DNA and incubated at room temperature for 15min with rotation. Beads were then washed twice with 500ul TWB during 2min at 55ºC in an orbital shaker followed by another wash in 100ul TD buffer (10 mM Tris-HCl pH 7.5, 5mM magnesium chloride, 10% dimethylformamide). A volume of a 1/20 TD buffer dilution of transposase Tn5 equivalent to 1ul/ng of ChIP DNA was brought to 50ul with TD buffer and added to

beads. Samples were incubated at 55ºC for 10min with intermitent shaking. Beads were then retained in a magnet and 100ul of 50mM EDTA was added followed by incubation at 50ºC for 30min with intermitent shaking. Beads were then sequentially washed twice with 100ul 50mM EDTA at 50ºC for 3min, twice with 100ul TWB at 55ºC for 2min and lastly with 100ul 10mM Tris pH 7.5. Beads were resuspended in 50ul of Phusion HF master mix [25 µL of Phusion HF 2X (MO5315, New England Biolabs), 2.5 µL of each 5uM Nextera Ad1.1 and Nextera Ad2.x and 20ul of water] and PCR carried out as follows: 5min at 72ºC, 1min at 98ºC, and 13 cycles of 15s at 98ºC, 30s at 63ºC and 1min at 72ºC. Libraries were made and purified by PAGE as for ChIP-seq and submitted for sequencing.

RNA-seq

Inducible sh*KDM4C* infected HCC1954, SUM149, HCC1806 and HDQP1 cells were plated in corresponding replicates following control (no doxycycline, DMSO), sh*KDM4C* induction (plus doxycycline), 10 µM ML324 (no doxycycline), 1 µM QC6352 (no doxycycline) treatment for 5 days treatment. Total RNA was extracted using the RNeasy Mini Kit (Qiagen). RNA-seq libraries were prepared using Illumina TruSeq Stranded mRNA sample preparation kits from 500 ng of purified total RNA according to the manufacturer's protocol. The finished dsDNA libraries were quantified by Qubit fluorometer, Agilent TapeStation 2200, and RT-qPCR using the Kapa Biosystems library quantification kit according to the manufacturer's protocols. Uniquely indexed libraries were pooled in equimolar ratios and sequenced on an Illumina NextSeq500 with single-end 75 bp reads in the Dana-Farber Cancer Institute Molecular Biology Core Facilities (HCC1954 and SUM149 samples) or Illumina NovaSeq6000 with paired-end 150bp reads with Azenta/Genewiz.

Metabolomic profiling

Inducible sh*KDM4C* infected HCC1954, SUM149, T47D and HCC70 parental cells were plated in duplicate in 3 biological replicates for each group following control (no doxycycline, DMSO), sh*KDM4C* induction (plus 1µg/ml doxycycline) or 10 µM ML324 (ML, no doxycycline) treatment in the first three

lines, and with or without 10 μM ML324 treatment in HCC70 for five days. Polar metabolites were extracted as described[24]. Briefly, media was removed by aspiration, and 80% methanol (pre-chilled to -80°C) was added to cell monolayers while working on dry ice. Cells were scraped, transferred to conical tubes, and centrifuged at full speed for 5 minutes at 4°C. The supernatant was collected on dry ice, and solvent was evaporated using a SpeedVac. Biological triplicates were submitted for polar metabolite profiling at the Beth Israel Deaconess Medical Center Mass Spectrometry Core Facility[24]. Pathway and Joint Pathway analysis were performed using MetaboAnalyst[25] and pathways with FDR < 0.05 were considered significant.

SUPPLEMENTARY REFERENCES

1. Guillen, K.P. *et al.* A human breast cancer-derived xenograft and organoid platform for drug discovery and precision oncology. *Nat Cancer* **3**, 232-250 (2022).
2. Sun, H. *et al.* Comprehensive characterization of 536 patient-derived xenograft models prioritizes candidatesfor targeted treatment. *Nat Commun* **12**, 5086 (2021).
3. Peluffo, G. *et al.* EN1 Is a Transcriptional Dependency in Triple-Negative Breast Cancer Associated with Brain Metastasis. *Cancer Res* **79**, 4173-4183 (2019).
4. Papachristou, E.K. *et al.* A quantitative mass spectrometry-based approach to monitor the dynamics of endogenous chromatin-associated protein complexes. *Nat Commun* **9**, 2311 (2018).
5. Johnson, W.E., Li, C. & Rabinovic, A. Adjusting batch effects in microarray expression data using empirical Bayes methods. *Biostatistics* **8**, 118-27 (2007).
6. Cornwell, M. *et al.* VIPER: Visualization Pipeline for RNA-seq, a Snakemake workflow for efficient and complete RNA-seq analysis. *BMC bioinformatics* **19**, 1-14 (2018).
7. Dobin, A. *et al.* STAR: ultrafast universal RNA-seq aligner. *Bioinformatics* **29**, 15-21 (2013).
8. Robinson, M.D., McCarthy, D.J. & Smyth, G.K. edgeR: a Bioconductor package for differential expression analysis of digital gene expression data. *Bioinformatics* **26**, 139-40 (2010).
9. Love, M.I., Huber, W. & Anders, S. Moderated estimation of fold change and dispersion for RNA-seq data with DESeq2. *Genome Biol* **15**, 550 (2014).
10. Hänzelmann, S., Castelo, R. & Guinney, J. GSVA: gene set variation analysis for microarray and RNA-seq data. *BMC bioinformatics* **14**, 1-15 (2013).
11. Wang, S. *et al.* Target analysis by integration of transcriptome and ChIP-seq data with BETA. *Nat Protoc* **8**, 2502-15 (2013).
12. Taing, L. *et al.* CHIPS: A Snakemake pipeline for quality control and reproducible processing of chromatin profiling data. *bioRxiv* (2021).
13. Li, H. Aligning sequence reads, clone sequences and assembly contigs with BWA-MEM. *arXiv preprint arXiv:1303.3997* (2013).
14. Feng, J., Liu, T., Qin, B., Zhang, Y. & Liu, X.S. Identifying ChIP-seq enrichment using MACS. *Nature protocols* **7**, 1728-1740 (2012).
15. Stempor, P. & Ahringer, J. SeqPlots-Interactive software for exploratory data analyses, pattern discovery and visualization in genomics. *Wellcome open research* **1**(2016).
16. Stark, R. & Brown, G. DiffBind: differential binding analysis of ChIP-Seq peak data. *R package version* **100**(2011).
17. Zhou, X. & Wang, T. Using the Wash U Epigenome Browser to examine genome-wide sequencing data. *Current protocols in bioinformatics* **40**, 10.10. 1-10.10. 14 (2012).
18. Quinlan, A.R. & Hall, I.M. BEDTools: a flexible suite of utilities for comparing genomic features. *Bioinformatics* **26**, 841-842 (2010).
19. Bailey, T.L., Johnson, J., Grant, C.E. & Noble, W.S. The MEME suite. *Nucleic acids research* **43**, W39-W49 (2015).
20. Kulakovskiy, I.V. *et al.* HOCOMOCO: towards a complete collection of transcription factor binding models for human and mouse via large-scale ChIP-Seq analysis. *Nucleic acids research* **46**, D252-D259 (2018).
21. Qiu, X. *et al.* CoBRA: Containerized Bioinformatics Workflow for Reproducible ChIP/ATAC-seq Analysis. *Genomics Proteomics Bioinformatics* **19**, 652-661 (2021).
22. Ramírez, F., Dündar, F., Diehl, S., Grüning, B.A. & Manke, T. deepTools: a flexible platform for exploring deep-sequencing data. *Nucleic acids research* **42**, W187-W191 (2014).
23. Mumbach, M.R. *et al.* Enhancer connectome in primary human cells identifies target genes of disease-associated DNA elements. *Nat Genet* **49**, 1602-1612 (2017).
24. Geck, R.C. *et al.* Inhibition of the polyamine synthesis enzyme ornithine decarboxylase sensitizes triple-negative breast cancer cells to cytotoxic chemotherapy. *J Biol Chem* **295**, 6263-6277 (2020).

25. Chong, J. *et al.* MetaboAnalyst 4.0: towards more transparent and integrative metabolomics analysis. *Nucleic Acids Res* **46**, W486-W494 (2018).
