# Supplementary material for: KDM4C inhibition blocks tumor growth in basal breast cancer by promoting cathepsin L-mediated histone H3 cleavage
Source: Nat Genet. 2025 Jun 2;57(6):1463–77. doi: 10.1038/s41588-025-02197-z (PMC12165855; doi:10.1038/s41588-025-02197-z)
Supplement: Supplementary file 2 — Reporting Summary [file 41588_2025_2197_MOESM2_ESM.pdf]

nature portfolio

Corresponding author(s): Kornelia Polyak

Last updated by author(s): Mar 25, 2025

Reporting Summary

Nature Portfolio wishes to improve the reproducibility of the work that we publish. This form provides structure for consistency and transparency in reporting. For further information on Nature Portfolio policies, see our Editorial Policies and the Editorial Policy Checklist.

Statistics

For all statistical analyses, confirm that the following items are present in the figure legend, table legend, main text, or Methods section.

n/a	Confirmed	
☐	☒	The exact sample size (*n*) for each experimental group/condition, given as a discrete number and unit of measurement
☐	☒	A statement on whether measurements were taken from distinct samples or whether the same sample was measured repeatedly
☐	☒	The statistical test(s) used AND whether they are one- or two-sided<br>*Only common tests should be described solely by name; describe more complex techniques in the Methods section.*
☐	☒	A description of all covariates tested
☐	☒	A description of any assumptions or corrections, such as tests of normality and adjustment for multiple comparisons
☐	☒	A full description of the statistical parameters including central tendency (e.g. means) or other basic estimates (e.g. regression coefficient) AND variation (e.g. standard deviation) or associated estimates of uncertainty (e.g. confidence intervals)
☐	☒	For null hypothesis testing, the test statistic (e.g. *F*, *t*, *r*) with confidence intervals, effect sizes, degrees of freedom and *P* value noted<br>*Give P values as exact values whenever suitable.*
☒	☐	For Bayesian analysis, information on the choice of priors and Markov chain Monte Carlo settings
☒	☐	For hierarchical and complex designs, identification of the appropriate level for tests and full reporting of outcomes
☐	☒	Estimates of effect sizes (e.g. Cohen's *d*, Pearson's *r*), indicating how they were calculated

Our web collection on statistics for biologists contains articles on many of the points above.

Software and code

Policy information about availability of computer code

Data collection	Images were acquired by Nikon Eclipse microscope and Zeiss 980 Confocal Imaging System. Bulk RNA-seq/ChIP-seq/Hi-ChIP/ATAC-seq were performed using an Illumina NextSeq500 or NovaSeq 6000 instrument. Immunoblots were imaged using ChemiDoc MP imaging system (BioRad). Histone mass spectrometry was analyzed with Q Exactive Plus Orbitrap (Thermo Fisher Scientific). Metabolomic profiling was analyzed using a 5500 QTRAP hybrid triple quadrupole mass spectrometer (AB/SCIEX) coupled to a Prominence UFLC HPLC system (Shimadzu) with HILIC chromatography (Waters Amide XBridge). Fluorescence and luminescence data were read by BioTek Synergy 2 Alpha Microplate Reader. qPLRX-RIME data were collected by Dionex Ultimate 3000 UHPLC system coupled with the LTQ Orbitrap Velos mass spectromete. Flow cytometry data was collected using LSRFortessa High-Parameter Flow Cytometer.
Data analysis	Statistical analyses were performed using GraphPad Prism (v10.3.1) or R(v4.3.1). Immunofluorescence and immune blot data was analyzed using ImageJ(v1.53q). Synergy was assessed based on BLISS model using SynergyFinder (v2.0). ChIPseq peak calling was performed using CHIPs pipeline and analyzed by Seqplots (v.1.12.0), BEDtools (v. 2.30.0), deepTools (v.3.5.0). RNAseq experiments were analyzed using STAR (v2.5.1b), DESeq2 (v1.41.1), edgeR (v 3.14), Salmon (v0.14.1) and GSVA (v. 1.34.0). Hi-ChIP data were analyzed by HiC-Pro (v.3.1.0) pipeline. Differential interaction sites were analyzed by HiTC (v. 1.38.0) and HiCCompare (v. 1.16.0). Metabolomic data was analyzed using MetaboAnalyst (v4.0). Histone Mass Spec data were analyzed using Sky (v4.0) and Spectrum Mill (v7.0) software package. IP-mass spec data were analyzed using ProTIGY interactive, visualization tool (v0.7.5). Flow cytometry data were analyzed using BD FACSDiva (v9.0) and FlowJo (v10.10).

For manuscripts utilizing custom algorithms or software that are central to the research but not yet described in published literature, software must be made available to editors and reviewers. We strongly encourage code deposition in a community repository (e.g. GitHub). See the Nature Portfolio guidelines for submitting code & software for further information.

Data

Policy information about availability of data

All manuscripts must include a data availability statement. This statement should provide the following information, where applicable:

- Accession codes, unique identifiers, or web links for publicly available datasets
- A description of any restrictions on data availability
- For clinical datasets or third party data, please ensure that the statement adheres to our policy

All data needed to evaluate the conclusions in the paper are present in the paper and/or the Supplemental Information. All raw and processed genomic data was deposited to GEO under accession number: GSE199913. All the genomic data were aligned to human reference genome GRCh37/hg19 (https://www.ncbi.nlm.nih.gov/datasets/genome/GCF_000001405.13/). RIME data are available via ProteomeXchange with identifier PXD031768. The original mass spectra, spectral library, and the protein sequence database used for searches have been deposited in the public proteomics repository MassIVE (identifier MSV000096930, http://massive.ucsd.edu) and are accessible at ftp://massive.ucsd.edu/v09/MSV000096930/. The mRNA expression data and the clinical data of TCGA and METABRIC were downloaded from TCGA data portal (https://portal.gdc.cancer.gov) and Synapse (Syn1688369) respectively. For TCGA, RNA-seq reads were reprocessed using Salmon v0.14.1103 and Log2 (TPM+1) values were used. For genes with multiple probes in METABRIC, probes with the highest inter-quartile range (IQR) were selected to represent the gene. Copy number information of KDM4C from TCGA and METABRIC were downloaded from cBioPortal predicted by GISTIC algorithm104. Fifty-seven breast cancer cell line copy number, lineage information and RNA-seq (FPKM) were downloaded from Cancer Cell Lin Encyclopedia105. AUC towards different chemotherapy drugs were downloaded from DepMap (https://depmap.org/portal/)52. Microarray data from five neoadjuvant therapy TNBC cohort were downloaded from GSE32646, GSE32603, GSE20194, GSE25066 and GSE18864 respectively. Log2 normalized probe intensities was used for signature enrichment analysis. The mRNA and normalized metabolomic profile data from FUSCC cohort were downloaded from Gong et. al. and GSE118527. For the KDM4C peak overlap analysis, public available data for H3K27ac ChIP-seq were downloaded from GSE72956 (HCC1954), GSE57436 (MCF7), GSE65201 (T47D). H3K4me3 ChIP-seq were downloaded from GSE54693 (MCF7) and GSE80592 (T47D).

Field-specific reporting

Please select the one below that is the best fit for your research. If you are not sure, read the appropriate sections before making your selection.

☒ Life sciences ☐ Behavioural & social sciences ☐ Ecological, evolutionary & environmental sciences

For a reference copy of the document with all sections, see nature.com/documents/nr-reporting-summary-flat.pdf

Life sciences study design

All studies must disclose on these points even when the disclosure is negative.

Sample size	Sample size for each experiment is indicated in the legend. No statistical methods were used to predetermine sample sizes. The sample size was chosen empirically to provide a sufficient level of statistical power for detecting indicated biological effects based on previous published literatures (e.g. PMID 26409824, 31239270), with a minimal n=4, maximum n=12, mostly n=5-10 for in vivo experiments.
Data exclusions	Sequencing data that did not meet the QC was excluded
Replication	High throughput sequencing, large cell line panel examination, screen, mass spectrometry, animal experiments and clinical sample-related experiments were performed once due to time, cost and resource limitations, while sufficient number of biological replicates were included when applicable. Data present in Extended Data Fig. 2g, 3j-k, 5g, 5h, 5k, 6b, 6d, 6i, 7d, 7e, 7k-n, 8e, 9b, 9i, and Supplementary Figure 3 were performed once with technical replicates. All the other experiments were performed at least 2-3 times with successful replication showing similar observations. Key experiments have been repeated by multiple different personnel at different times. Number of replicates and sample size are indicated in the figure legends.
Randomization	Mice were randomized to treatment groups after they developed palpable tumors.For in vitro studies, randomization was not applied because the samples (e.g., cultured cells and other biological materials) exhibit high homogeneity, and they are relatively consistent across different replicates. This ensures that the experimental and control groups have similar baseline levle withoutr the need for randomization.
Blinding	Sequencing data processing was performed by bioinformaticians blinded to the identity of samples. For most in vitro assay blinding was not possible as samples had to be labeled. The investigators were not blinded to allocation during other experiments and outcome assessment.

Reporting for specific materials, systems and methods

We require information from authors about some types of materials, experimental systems and methods used in many studies. Here, indicate whether each material, system or method listed is relevant to your study. If you are not sure if a list item applies to your research, read the appropriate section before selecting a response.

Materials & experimental systems

n/a	Involved in the study
☐	☒ Antibodies
☐	☒ Eukaryotic cell lines
☒	☐ Palaeontology and archaeology
☐	☒ Animals and other organisms
☒	☐ Human research participants
☒	☐ Clinical data
☒	☐ Dual use research of concern

Methods

n/a	Involved in the study
☐	☒ ChIP-seq
☐	☒ Flow cytometry
☒	☐ MRI-based neuroimaging

Antibodies

Antibodies used

Rabbit polyclonal KDM4C anitbody Novus Biologicals Cat# NBP1-49600, RRID:AB_10011699 (WB/ChIP-seq/IF/IP)
Goat polyclonal Anti-cathepsin L antibody Novus Biologicals Cat# AF952, RRID:AB_355737 (ChIP-seq/Hi-ChIP/IF/IP)
Mouse monoclonal Anti-cathepsin L antibody (Clone 33/2) Novus Cat# NB100-1775, RRID:AB_10124480 (WB)
Mouse monoclonal Anti- α tubulin antibody (Clone B-5-1-2) Sigma-Aldrich Cat# T5168, RRID:AB_477579 (WB)
Rabbit monoclonal Anti-vinculin antibody (Clone E1E9V) Cell Signaling Technology Cat# 13901, RRID:AB_2728768 (WB)
Rabbit polyclonal Anti-GRHL2 antibody Sigma-Aldrich Cat# HPA004820, RRID:AB_1857928 (ChIP-seq/WB/IF/IP)
Rabbit polyclonal Anit-GCLC antibody Abcam Cat# ab53179, RRID:AB_880163 (WB/IF)
Rabbit polyclonal Anti-Histone H3 antibody (C-terminus) Abcam Cat# ab1791, RRID:AB_302613 (WB/ChIP-seq)
Rabbit polyclonal Anti-Histone H3 antibody (N-terminus) Abcam Cat# ab18521, RRID:AB_732917 (WB)
Rabbit polyclonal Anti-GFP antibody Novus Biologicals Cat# NB600-308, RRID:AB_10003058 (ChIP-seq/WB)
Rabbit polyclonal Anti-V5 Tag antibody Novus Biologicals Cat# NB600-381, RRID:AB_10001084 (ChIP-seq/WB)
Rabbit polyclonal Anti-Histone H3 (tri methyl K9) antibody Abcam Cat# ab8898, RRID:AB_306848 (ChIP-seq)
Rabbit polyclonal Anti-Histone H3 (tri methyl K36) antibody Abcam Cat# ab9050, RRID:AB_306966 (ChIP-seq)
Rabbit polyclonal Anti-Histone H3 (tri methyl K4) antibody Abcam Cat# ab8580, RRID:AB_306649 (ChIP-seq)
Rabbit polyclonal Anti-Histone H3 (acetylation K27) antibody Diagenode C15410196, RRID:AB_2637079 (ChIP-seq)
Rabbit polyclonal Anti-KDM4A antibody Bethyl Laboratories Cat# A300-861A, RRID:AB_609461 (ChIP-seq)
Rabbit polyclonal Anti-KDM4B antibody Active Motif Cat# 61222, RRID:AB_2615033 (ChIP-seq)
Rabbit monoclonal Anti-AIF antibody (Clone D39D2) Cell Signaling Technology Cat# 5318, AB_10634755 (WB)
Mouse polyclonal Anti-Histone H3 antibody Active Motif Cat# 39763, RRID:AB_2650522 (WB)
Rabbit monoclonal Anti-KDM4B antibody (Clone D7E6) Cell Signaling Technology Cat# 8639, RRID:AB_11140642(WB)
Rabbit monoclonal Anti-KDM4A antibody (CloneC37E5) Cell Signaling Technology Cat# 5328, RRID:AB_10828595 (WB)
Rabbit monoclonal Anti-HA-Tag antibody (Clone C29F4) Cell Signaling Technology Cat# 3724,RRID:AB_1549585 (WB)
Rabbit polyclonal Anti-pan methyl Lysine antibody Abcam Cat# ab7315, RRID:AB_305840 (IP)
Mouse monoclonal Anti-CTCF antibody (clone 48) BD Biosciences Cat# 612149, RRID:AB_399520 (WB)
Rabbit IgG Isotype Control, Thermo Fisher Scientific, Cat# 31887, RRID:AB_2532177 (IP)
Goat IgG Isotype Control, Novus, Cat# NB410-28088, RRID:AB_1853319 (IP)
Goat anti-Rabbit IgG (H+L) Secondary Antibody Thermo Fischer Scientific Cat#65-6120,RRID:AB_2533967 (WB)
Goat anti-Mouse IgG (H+L) Secondary Antibody Thermo Fischer Scientific Cat#62-6520,RRID:AB_2533947(WB)
Rabbit anti-Goat IgG (H+L) Secondary Antibody Thermo Fischer Scientific Cat#81-1620,RRID:AB_2534006(WB)

Validation

Rabbit polyclonal KDM4C anitbody Vacliated for reactivity towards human Application: Immunoblot: 1:1000; Immuofluoresence: 1:100; ChIP-seq:5 µg; Immuno precipitation:5 µg Product info: https://www.novusbio.com/products/lysine-k-specific-demethylase-4c-kdm4c-jmjd2c-antibody_nbp1-49600?srsltid=AfmBOoqmd0j5ZL00l-t3ANOqrpb2-kSudBRwzYeSQGRI4YuHAoXmE4EK
Goat polyclonal Anti-cathepsin L antibody Vacliated for reactivity towards human Application: Immunoblot: 1:1000; Immuofluoresence: 1:100; ChIP-seq:5 µg; Immuno precipitation:5 µg Product info:https://www.rndsystems.com/products/human-cathepsin-l-antibody_af952
Mouse monoclonal Anti-cathepsin L antibody (Clone 33/2) Vacliated for reactivity towards human Application: Immunoblot: 1:1000 Product info:https://www.novusbio.com/products/cathepsin-l-antibody-33-2_nb100-1775?srsltid=AfmBOopCCoWHK-W1OSqvMXCxpFOaVV-HvUhkI2duacXylJ5MM3nFmJNI
Mouse monoclonal Anti- α tubulin antibody (Clone B-5-1-2) Vacliated for reactivity towards human Application: Immunoblot: 1:5000 Product info:https://www.sigmaaldrich.com/US/en/product/sigma/t5168?srsltid=AfmBOoqlVRPraazSYrRKtTs0VHhogR02DdUMsSFI5HPhswHjyEVZua1X
Rabbit monoclonal Anti-vinculin antibody (Clone E1E9V) Vacliated for reactivity towards human Application: Immunoblot: 1:1000 Product info:https://www.cellsignal.com/products/primary-antibodies/vinculin-e1e9v-xp-rabbit-mab/13901?srsltid=AfmBOopHeR-cRxmZBHWKy1R4Jd5ESDDClsh_9-3lG1nWGMRQ1BQxi1fS
Rabbit polyclonal Anti-GRHL2 antibody Vacliated for reactivity towards human Application: Immunoblot: 1:1000; Immuofluoresence: 1:100; ChIP-seq:5 µg; Immuno precipitation:5 µg Product info:https://www.sigmaaldrich.com/US/en/product/sigma/hpa004820?srsltid=AfmBOoo8PYd7bsydb7k1QADl4yGAmWX--T3t6ZdBrFkhtf2VG1r0sw8e
Rabbit polyclonal Anit-GCLC antibody Vacliated for reactivity towards human Application: Immunoblot: 1:1000; Immuofluoresence: 1:100 Product info:https://www.abcam.com/en-us/products/primary-antibodies/gclc-antibody-ab53179
Rabbit polyclonal Anti-Histone H3 antibody (C-terminus) Vacliated for reactivity towards human Application: Immunoblot: 1:1000; ChIP-seq:5 µg Product info:https://www.abcam.com/en-us/products/primary-antibodies/histone-h3-antibody-nuclear-marker-and-chip-grade-ab1791
Rabbit polyclonal Anti-Histone H3 antibody (N-terminus) Vacliated for reactivity towards human Application: Immunoblot: 1:1000 Product info:https://www.abcam.com/en-us/products/primary-antibodies/histone-h3-antibody-ab18521
Rabbit polyclonal Anti-GFP antibody Vacliated for reactivity towards human Application: Immunoblot: 1:1000; ChIP-seq:5 µg Product info:https://www.novusbio.com/products/gfp-antibody_nb600-308?srsltid=AfmBOoqGduykvVqYNHY9P-KQtFiu6SzieSeVFbYvmHMNHO0X64AoUmuA

Rabbit polyclonal Anti-V5 Tag antibody Vacliated for reactivity towards human Application: Immunoblot: 1:1000; ChIP-seq:5 µg Product info:https://www.novusbio.com/products/v5-epitope-tag-antibody_nb600-381?srsltid=AfmBOoo2xPUimSUVvU_PgZyN5TJwhfsnlqD50vthOUsaPPNjJxZoZKZw
Rabbit polyclonal Anti-Histone H3 (tri methyl K9) antibody Vacliated for reactivity towards human Application: ChIP-seq:5 µg Product info:https://www.abcam.com/en-us/products/primary-antibodies/histone-h3-tri-methyl-k9-antibody-chip-grade-ab8898?srsltid=AfmBOooFMQga1E8Julh6ZSuQ26HRnugH7c5XAlwnPa--v1nODRysTZcW
Rabbit polyclonal Anti-Histone H3 (tri methyl K36) antibody Vacliated for reactivity towards human Application: ChIP-seq:5 µg Product info:https://www.abcam.com/en-us/products/primary-antibodies/histone-h3-tri-methyl-k36-antibody-chip-grade-ab9050?srsltid=AfmBOopqshYCtnAj05BKZCj_zci-3VhTGwj0lalyCaSqwVIcVYfpTv2H
Rabbit polyclonal Anti-Histone H3 (tri methyl K4) antibody Vacliated for reactivity towards human Application: Immunoblot: 1:1000; ChIP-seq:5 µg Product info:https://www.abcam.com/en-us/products/primary-antibodies/histone-h3-tri-methyl-k4-antibody-chip-grade-ab8580?srsltid=AfmBOopIzhSLZ07orJvzoYoLNrZoLCJtg8Ui5Asw_6elGbTt-FQl26SV
Rabbit polyclonal Anti-Histone H3 (acetylation K27) antibody Vacliated for reactivity towards human Application: ChIP-seq:5 µg Product info:https://www.diagenode.com/en/p/h3k27ac-polyclonal-antibody-premium-50-mg-18-ml
Rabbit polyclonal Anti-KDM4A antibody Vacliated for reactivity towards human Application: ChIP-seq:5 µg Product info:https://www.fortislife.com/products/primary-antibodies/rabbit-anti-jmjd2a-antibody/BETHYL-A300-861
Rabbit polyclonal Anti-KDM4B antibody Vacliated for reactivity towards human Application: ChIP-seq:5 µg Product info:https://www.activemotif.com/catalog/details/61221/jmjd2b-kdm4b-antibody-pab
Rabbit monoclonal Anti-AIF antibody (Clone D39D2) Vacliated for reactivity towards human Application: Immunoblot: 1:1000 Product info:https://www.cellsignal.com/products/primary-antibodies/aif-d39d2-xp-rabbit-mab/5318?srsltid=AfmBOoquAXQCfZ0DsNN2sab46XFhYfHNxvqSZgS6zVga1QnvQotbeyZO
Mouse polyclonal Anti-Histone H3 antibody Vacliated for reactivity towards human Application: Immunoblot: 1:1000 Product info:https://www.activemotif.com/catalog/details/39763
Rabbit monoclonal Anti-HA-Tag antibody (Clone C29F4) Vacliated for reactivity towards human Application: Immunoblot: 1:1000 Product info:https://www.cellsignal.com/products/primary-antibodies/ha-tag-c29f4-rabbit-mab/3724?srsltid=AfmBOoouv8Hv9PPIhbO4aMuHs_rJzgnwOV5b65L1Xx3gDrf4as5TFKBv
Rabbit monoclonal Anti-KDM4B antibody (Clone D7E6) Vacliated for reactivity towards human Application: Immunoblot: 1:1000 Product info:https://www.cellsignal.com/products/primary-antibodies/jmjd2b-d7e6-rabbit-mab/8639?srsltid=AfmBOorlcAbi4QUEOLckZnxb-zFoVWi5n2v8eqwdlGzTDPTpW3mQUtDA
Rabbit monoclonal Anti-KDM4A antibody (CloneC37E5) Vacliated for reactivity towards human Application: Immunoblot: 1:500 Product info:https://www.cellsignal.com/products/primary-antibodies/jmjd2a-c37e5-rabbit-mab/5328?srsltid=AfmBOooD9i9hovy-cABC6f-HNEzmRWapVYFce96dMJQUq5VMTsE67dxS
Rabbit polyclonal Anti-pan methyl Lysine antibody Vacliated for reactivity towards human Application: Immuno precipitation:5 µg Product info:https://www.abcam.com/en-us/products/primary-antibodies/pan-methyl-lysine-antibody-chip-grade-ab7315?srsltid=AfmBOopam9TnArn1iVtvMS3B_j1_GWty_QDw3rN-_-oe92d2jXksS1sM
Mouse monoclonal Anyi-CTCF antibody (Clone 48) Vacliated for reactivity towards human Application: Immunoblot: 1:1000 Product info:https://www.bdbiosciences.com/en-us/products/reagents/microscopy-imaging-reagents/immunofluorescence-reagents/purified-mouse-anti-ctcf.612149?tab=product_details

Eukaryotic cell lines

Policy information about cell lines

Cell line source(s)

BT549 cell line ATCC HTB-122
CAL120 cell line ATCC ACC 459
CAL51 cell line DSMZ ACC 302
CAL851 cell line DSMZ ACC 440
DU4475 cell line ATCC HTB-123
HCC1143 cell line ATCC CRL-2321
HCC1187 cell line ATCC CRL-2322
HCC1395 cell line ATCC CRL-2324
HCC1569 cell line ATCC CRL-2330
HCC1806 cell line ATCC CRL-2335
HCC1937 cell line ATCC CRL-2336
HCC1954 cell line ATCC CRL-2338
HCC2157 cell line ATCC CRL-2340
HCC38 cell line ATCC CRL-2314
HCC70 cell line ATCC CRL-2315
HDQ-P1 cell line DSMZ ACC 494
HS578T cell line ATCC HTB-126
MCF7 cell line ATCC HTB-22
MDA-MB-231 cell line ATCC HTB-26
MDA-MB-436 cell line ATCC HTB-130
MDA-MB-468 cell line ATCC HTB-132
SUM1315 cell line Stephen Ethier, University of Michigan
SUM149 cell line Stephen Ethier, University of Michigan
SUM159 cell line Stephen Ethier, University of Michigan
SUM3153 cell line Stephen Ethier, University of Michigan
T47D cell line ATCC HTB-133
293FT cell line Thermo Fisher Scientific R70007

Authentication

The identity of the cell lines was confirmed based on STR and exome-seq analyses.

Mycoplasma contamination

Cell lines were routinely tested for mycoplasma and rodent pathogen contamination. No contamination was found at any

Mycoplasma contamination	time point.
Commonly misidentified lines (See ICLAC register)	No commonly misidentified lines were used in this study.

Animals and other organisms

Policy information about studies involving animals; ARRIVE guidelines recommended for reporting animal research

Laboratory animals	For xenograft assays using KDM4C knockdown HCC1954 and SUM149 models, and cisplatin/QC6352/BSO drug combination assay, female NCr nude (CrTac:NCr-Foxn1nu) mice were purchased from Taconic Biosciences at 5-6 weeks of age. For experiments using HCI-041 PDX, KDM4C knockdown HCC1806, and CTSL knockout SUM149 and HCC1806 models, female NSG (NOD.Cg-Prkdcscid Il2rgtm1Wjl/SzJ) mice were purchased from The Jackson Laboratory at 5-6 weeks of age. Mice were housed 5 to a cage with ad libitum access to food and water in 20C ambient temperature, 40-50% humidity, and 12-hour light/12-hour dark cycle.
Wild animals	No wild animals were used in this study.
Field-collected samples	No field-collected samples were used in this study.
Ethics oversight	Animal studies were performed according to protocol 11-023 approved by the Dana-Farber Cancer Institute Animal Care and Use Committee.

Note that full information on the approval of the study protocol must also be provided in the manuscript.

ChIP-seq

Data deposition

☒ Confirm that both raw and final processed data have been deposited in a public database such as GEO.

☒ Confirm that you have deposited or provided access to graph files (e.g. BED files) for the called peaks.

Data access links *May remain private before publication.*	The ChIP-seq data has been deposited as a SubSeries in GEO under the SuperSeries ID code (GSE199913).

Files in database submission

Sample Name processed data file (Bigwig) raw fastq files (first 87 samples are single-end, last 10 samples are paried-end)
HCC1954_shKDM4C_17_Control_Input NA 20140623-HCC1954Input-GP1109_S3_R1.fastq.gz
HCC1954_shKDM4C-17_Control_K9me3_1 HCC1954_shKDM4C-17_Control_K9me3_2.rep1_treat_pileup.bw 20151226-HCC1954-CONTROL-K9me3-GP2601_S5_R1_001.fastq.gz
HCC1954_shKDM4C-17_Control_K9me3_2 HCC1954_shKDM4C-17_Control_KDM4C_1.rep1_treat_pileup.bw 20161016_1954C_K9_C3_GP3575_S5_R1_001.fastq.gz
HCC1954_shKDM4C-17_Doxy_K9me3_1 HCC1954_shKDM4C-17_Doxy_K9me3_1.rep1_treat_pileup.bw 20151226-HCC1954-DOXY-K9me3-GP2601_S6_R1_001.fastq.gz
HCC1954_shKDM4C-17_Doxy_K9me3_2 HCC1954_shKDM4C-17_Doxy_K9me3_2.rep1_treat_pileup.bw 20161016_1954D_K9_C4_GP3575_S6_R1_001.fastq.gz
HCC1954_shKDM4C-17_ML324_K9me3_1 HCC1954_shKDM4C-17_ML326_K9me3_1.rep1_treat_pileup.bw 20151226-HCC1954-ML-K9me3-GP2601_S7_R1_001.fastq.gz
HCC1954_shKDM4C-17_ML324_K9me3_2 HCC1954_shKDM4C-17_ML327_K9me3_2.rep1_treat_pileup.bw 20161016_1954M_K9_C5_GP3575_S7_R1_001.fastq.gz
HCC1954_shKDM4C-17_Control_K36me3_1 HCC1954_shKDM4C-17_Control_K36me3_1.rep1_treat_pileup.bw 20151222-HCC1954-Control-K36me3-GP2600_S3_R1_001.fastq.gz
HCC1954_shKDM4C-17_Control_K36me3_2 HCC1954_shKDM4C-17_Control_K36me3_2.rep1_treat_pileup.bw 20161129_1954_C_K36_GP3718_S1_R1_001.fastq.gz
HCC1954_shKDM4C-17_Doxy_K36me3_1 HCC1954_shKDM4C-17_Doxy_K36me3_1.rep1_treat_pileup.bw 20151222-HCC1954-Doxy-K36me3-GP2600_S4_R1_001.fastq.gz
HCC1954_shKDM4C-17_Doxy_K36me3_2 HCC1954_shKDM4C-17_Doxy_K36me3_2.rep1_treat_pileup.bw 20161129_1954_D_K36_GP3718_S2_R1_001.fastq.gz
HCC1954_shKDM4C-17_ML324_K36me3_1 HCC1954_shKDM4C-17_ML328_K36me3_1.rep1_treat_pileup.bw 20151222-HCC1954-ML324-K36me3-GP2600_S5_R1_001.fastq.gz
HCC1954_shKDM4C-17_ML324_K36me3_2 HCC1954_shKDM4C-17_ML329_K36me3_2.rep1_treat_pileup.bw 20161129_1954_M_K36_GP3718_S3_R1_001.fastq.gz
HCC1954_shKDM4C-17_Control_KDM4C_1 HCC1954_shKDM4C-17_Control_KDM4C_1.rep1_treat_pileup.bw 20151218-HCC1954-Control-JMJ-GP2598_S1_R1_001.fastq.gz
HCC1954_shKDM4C-17_Control_KDM4C_2 HCC1954_shKDM4C-17_Control_KDM4C_2.rep1_treat_pileup.bw 20161016_1954C_JMJ_A11_GP3573_S9_R1_001.fastq.gz
HCC1954_shKDM4C-17_Control_H3K4me3 HCC1954_shKDM4C-17_Control_H3K4me3.rep1_treat_pileup.bw 20161016_1954C_K4_B1_GP3573_S3_R1_001.fastq.gz
HCC1954_shKDM4C-17_Doxy_H3K4me3 HCC1954_shKDM4C-17_Doxy_H3K4me3.rep1_treat_pileup.bw 20161016_1954D_K4_B2_GP3573_S4_R1_001.fastq.gz
HCC1954_shKDM4C-17_ML324_H3K4me3 HCC1954_shKDM4C-17_ML330_H3K4me3.rep1_treat_pileup.bw 20161016_1954M_K4_B3_GP3573_S5_R1_001.fastq.gz
SUM149_shKDM4C_17_Control_Input_3 NA 180406_S149_17C_INPUT_GP5435_S7_R1_001.fastq.gz
SUM149_shKDM4C_17_Control_K9me3_1 SUM149_shKDM4C-17_Control_K9me3_1.rep1_treat_pileup.bw 20150727-N149-K9-GP2134_S1_R1_001.fastq.gz
SUM149_shKDM4C_17_Control_K9me3_2 SUM149_shKDM4C-17_Control_K9me3_2.rep1_treat_pileup.bw

20150917-149-17-K9me3-utube-GP2281_S11_R1_001.fastq.gz
SUM149_shKDM4C_17_Control_K9me3_3 SUM149_shKDM4C-17_Control_K9me3_3.rep1_treat_pileup.bw 20150917-149-K9me3-Abcam150uM-GP2281_S6_R1_001.fastq.gz
SUM149_shKDM4C_17_Control_K9me3_4 SUM149_shKDM4C-17_Control_K9me3_4.rep1_treat_pileup.bw 20151226-SUM149-Control-K9me3-GP2601_S8_R1_001.fastq.gz
SUM149_shKDM4C_17_Control_K9me3_5 SUM149_shKDM4C-17_Control_K9me3_5.rep1_treat_pileup.bw 20161016_149C_K9_C6_GP3575_S8_R1_001.fastq.gz
SUM149_shKDM4C_17_Doxy_K9me3_1 SUM149_shKDM4C-17_Doxy_K9me3_1.rep1_treat_pileup.bw 20150727-D149-K9-GP2134_S2_R1_001.fastq.gz
SUM149_shKDM4C_17_Doxy_K9me3_2 SUM149_shKDM4C-17_Doxy_K9me3_2.rep1_treat_pileup.bw 20151226-SUM149-Doxy-K9me3-GP2601_S9_R1_001.fastq.gz
SUM149_shKDM4C_17_Doxy_K9me3_3 SUM149_shKDM4C-17_Doxy_K9me3_3.rep1_treat_pileup.bw 20161016_149D_K9_C7_GP3575_S9_R1_001.fastq.gz
SUM149_shKDM4C_17_ML324_K9me3_1 SUM149_shKDM4C-17_ML324_K9me3_1.rep1_treat_pileup.bw 20150727-M149-K9-GP2134_S3_R1_001.fastq.gz
SUM149_shKDM4C_17_ML324_K9me3_2 SUM149_shKDM4C-17_ML324_K9me3_2.rep1_treat_pileup.bw 20151226-SUM149-ML324-K9me3-GP2601_S10_R1_001.fastq.gz
SUM149_shKDM4C_17_ML324_K9me3_3 SUM149_shKDM4C-17_ML324_K9me3_3.rep1_treat_pileup.bw 20161016_149M_K9_C8_GP3575_S10_R1_001.fastq.gz
SUM149_shKDM4C-17_Control_K36me3_1 SUM149_shKDM4C-17_Control_K36me3_1.rep1_treat_pileup.bw 20150727-N149-K36-GP2134_S6_R1_001.fastq.gz
SUM149_shKDM4C-17_Control_K36me3_2 SUM149_shKDM4C-17_Control_K36me3_2.rep1_treat_pileup.bw 20150917-149-17-K36me3-utube-GP2281_S12_R1_001.fastq.gz
SUM149_shKDM4C-17_Control_K36me3_3 SUM149_shKDM4C-17_Control_K36me3_3.rep1_treat_pileup.bw 20151222-SUM149-Control-K36me3-GP2600_S6_R1_001.fastq.gz
SUM149_shKDM4C-17_Control_K36me3_4 SUM149_shKDM4C-17_Control_K36me3_4.rep1_treat_pileup.bw 20161129_149_C_K36_GP3718_S4_R1_001.fastq.gz
SUM149_shKDM4C-17_Control_K36me3_5 SUM149_shKDM4C-17_Control_K36me3_5.rep1_treat_pileup.bw 20141126-SUM149-No-K36me3-GP1520_S4_R1.fastq.gz
SUM149_shKDM4C_17_Doxy_K36me3_1 SUM149_shKDM4C-17_Doxy_K36me3_1.rep1_treat_pileup.bw 20150727-D149-K36-GP2134_S7_R1_001.fastq.gz
SUM149_shKDM4C_17_Doxy_K36me3_2 SUM149_shKDM4C-17_Doxy_K36me3_2.rep1_treat_pileup.bw 20151222-SUM149-Doxy-K36me3-GP2600_S7_R1_001.fastq.gz
SUM149_shKDM4C_17_Doxy_K36me3_3 SUM149_shKDM4C-17_Doxy_K36me3_3.rep1_treat_pileup.bw 20141126-SUM149-Doxy-K36me3-GP1520_S5_R1.fastq.gz
SUM149_shKDM4C_17_ML324_K36me3_1 SUM149_shKDM4C-17_ML324_K36me3_1.rep1_treat_pileup.bw 20150727-M149-K36-GP2134_S8_R1_001.fastq.gz
SUM149_shKDM4C_17_ML324_K36me3_2 SUM149_shKDM4C-17_ML324_K36me3_2.rep1_treat_pileup.bw 20151222-SUM149-ML324-K36me3-GP2600_S8_R1_001.fastq.gz
SUM149_shKDM4C_17_ML324_K36me3_3 SUM149_shKDM4C-17_ML324_K36me3_3.rep1_treat_pileup.bw 20161129_149_M_K36_GP3718_S5_R1_001.fastq.gz
SUM149_shKDM4C_17_ML324_K36me3_4 SUM149_shKDM4C-17_ML324_K36me3_4.rep1_treat_pileup.bw 20141126-SUM149-ML-K36me3-GP1520_S6_R1.fastq.gz
SUM149_shKDM4C-17_Control_H3K4me3 SUM149_shKDM4C-17_Control_H3K4me3.rep1_treat_pileup.bw 20161016_149C_K4_B4_GP3573_S6_R1_001.fastq.gz
SUM149_shKDM4C-17_Doxy_H3K4me3 SUM149_shKDM4C-17_Doxy_H3K4me3.rep1_treat_pileup.bw 20161016_149D_K4_B5_GP3573_S7_R1_001.fastq.gz
SUM149_shKDM4C-17_ML324_H3K4me3 SUM149_shKDM4C-17_ML324_H3K4me3.rep1_treat_pileup.bw 20161016_149M_K4_B6_GP3573_S8_R1_001.fastq.gz
SUM149_shKDM4C-17_Control_KDM4C_1 SUM149_shKDM4C-17_Control_KDM4C_1.rep1_treat_pileup.bw 20150728-N149J-GP2135_S1_R1_001.fastq.gz
SUM149_shKDM4C-17_Control_KDM4C_2 SUM149_shKDM4C-17_Control_KDM4C_2.rep1_treat_pileup.bw 20150917-149-17-JMJD2C-utube-GP2281_S10_R1_001.fastq.gz
SUM149_shKDM4C-17_Control_KDM4C_3 SUM149_shKDM4C-17_Control_KDM4C_3.rep1_treat_pileup.bw 20151218-SUM149-Control-JMJ-GP2598_S4_R1_001.fastq.gz
SUM149_shKDM4C-17_Control_KDM4C_4 SUM149_shKDM4C-17_Control_KDM4C_4.rep1_treat_pileup.bw 20161016_149C_JMJ_B1_GP3575_S1_R1_001.fastq.gz
SUM149_Parental_Control_K27me3 SUM149_Parental_Control_K27me3.rep1_treat_pileup.bw 20180516_S149_C_K27m3_GP5611_S13_R1_001.fastq.gz
HCC70_Parental_Untreated_INPUT NA 20150728-H70I-GP2135_S6_R1_001.fastq.gz
HCC70_Parental_Untreated_KDM4C HCC70_Parental_Untreated_KDM4C.rep1_treat_pileup.bw 20150728-H70J-GP2135_S4_R1_001.fastq.gz
MCF7_Parental_Untreated_KDM4C MCF7_Parental_Untreated_KDM4C.rep1_treat_pileup.bw 20150728-M7J-GP2135_S5_R1_001.fastq.gz
MCF7_Parental_Untreated_K9me3 MCF7_Parental_Untreated_K9me3.rep1_treat_pileup.bw 20150727-M7-K9-GP2134_S5_R1_001.fastq.gz
MCF7_Parental_Untreated_K36me3 MCF7_Parental_Untreated_K36me3.rep1_treat_pileup.bw 20150727-M7-K36-GP2134_S10_R1_001.fastq.gz
HCC2157_Parental_Untreated_KDM4C HCC2157_Parental_Untreated_KDM4C.rep1_treat_pileup.bw 20161216_HCC2157_JMJ_GP3794_S9_R1_001.fastq.gz
T47D_shKDM4C_17_Control_INPUT NA 20150506-T47D-DMSO-INPUT-GP1906_S7_R1.fastq.gz
T47D_shKDM4C-17_Control_KDM4C T47D_shKDM4C-17_Control_KDM4C.rep1_treat_pileup.bw 20161016_T47DC_JMJ_B3_GP3575_S3_R1_001.fastq.gz
T47D_shKDM4C_17_Control_K9me3_1 T47D_shKDM4C-17_Control_K9me3_1.rep1_treat_pileup.bw 20170311_T47D_C_K9_GP4112_S6_R1_001.fastq.gz
T47D_shKDM4C_17_Control_K36me3_1 T47D_shKDM4C-17_Control_k36me3_1.rep1_treat_pileup.bw

20161129_T47D_C_K36_GP3718_S6_R1_001.fastq.gz
SUM149_Parental_Control_INPUT NA 20180516_S149_C_INPUT_GP5612_S1_R1_001.fastq.gz
SUM149_Parental_ML324_INPUT NA 20180516_S149_M_INPUT_GP5612_S2_R1_001.fastq.gz
SUM149_Parental_Control_CTSL SUM149_Parental_Control_CTSL.rep1_treat_pileup.bw
20161215_149_C_CTL_GP3793_S7_R1_001.fastq.gz
SUM149_Parental_ML324_CTSL SUM149_Parental_ML324_CTSL.rep1_treat_pileup.bw
20161215_149_M_CTL_GP3793_S8_R1_001.fastq.gz
SUM149_Resistant_Control_CTSL SUM149_ML324-R_Control_CTSL.rep1_treat_pileup.bw
20161215_149_RC_CTL_GP3793_S9_R1_001.fastq.gz
SUM149_Resistant_ML324_CTSL SUM149_ML324-R_ML324_CTSL.rep1_treat_pileup.bw
20161215_149_RM_CTL_GP3793_S10_R1_001.fastq.gz
SUM149_Parental_Control_GRHL2 SUM149_Parental_Control_GRHL2.rep1_treat_pileup.bw
20180507_S149_C_GRHL2_GP5566_S5_R1_001.fastq.gz
SUM149_Parental_ML324_GRHL2 SUM149_Parental_ML324_GRHL2.rep1_treat_pileup.bw
20180507_S149_M_GRHL2_GP5566_S6_R1_001.fastq.gz
HCC1954_Parental_Control_INPUT NA 20180430_1954_C_INPUT_GP5539_S7_R1_001.fastq.gz
HCC1954_Parental_Control_CTSL HCC1954_Parental_Control_CTSL.rep1_treat_pileup.bw
20180430_1954_C_CTSL_GP5539_S1_R1_001.fastq.gz
HCC1954_Parental_Control_GRHL2 HCC1954_Parental_Control_GRHL2.rep1_treat_pileup.bw
20180430_1954_C_GRHL2_GP5539_S3_R1_001.fastq.gz
SUM149_C_KDM4A SUM149_C_KDM4A.rep1_treat_pileup.bw 20190310_SUM149_C_KDM4A_GP6745_S1_R1_001.fastq.gz
SUM149_C_KDM4B SUM149_C_KDM4B.rep1_treat_pileup.bw 20190310_SUM149_C_KDM4B_GP6745_S5_R1_001.fastq.gz
SUM149_C_KDM4C SUM149_C_KDM4C.rep1_treat_pileup.bw 20190310_SUM149_C_KDM4C_GP6745_S9_R1_001.fastq.gz
SUM149_M_KDM4A SUM149_M_KDM4A.rep1_treat_pileup.bw
20190310_SUM149_M_KDM4A_GP6745_S2_R1_001.fastq.gz
SUM149_M_KDM4B SUM149_M_KDM4B.rep1_treat_pileup.bw
20190310_SUM149_M_KDM4B_GP6745_S6_R1_001.fastq.gz
SUM149_M_KDM4C SUM149_M_KDM4C.rep1_treat_pileup.bw
20190310_SUM149_M_KDM4C_GP6745_S10_R1_001.fastq.gz
SUM149_sgGRHL2_C_CTSL SUM149_GRHL2_C_CTSL.rep1_treat_pileup.bw
20190310_SUM149_GRHL2_C_CTSL_GP6744_S11_R1_001.fastq.gz
SUM149_sgScramble_C_CTSL SUM149_ScrB_C_CTSL.rep1_treat_pileup.bw
20190310_SUM149_ScrB_C_CTSL_GP6744_S9_R1_001.fastq.gz
SUM149_V5_H3_C_Ct SUM149_V5_H3_C_Ct.rep1_treat_pileup.bw
20190310_SUM149_V5_H3_C_Ct_GP6744_S1_R1_001.fastq.gz
SUM149_V5_H3_C_V5 SUM149_V5_H3_C_V5.rep1_treat_pileup.bw
20190310_SUM149_V5_H3_C_V5_GP6744_S5_R1_001.fastq.gz
SUM149_V5_H3_M_Ct SUM149_V5_H3_M_Ct.rep1_treat_pileup.bw
20190310_SUM149_V5_H3_M_Ct_GP6744_S2_R1_001.fastq.gz
SUM149_V5_H3_M_V5 SUM149_V5_H3_M_V5.rep1_treat_pileup.bw
20190310_SUM149_V5_H3_M_V5_GP6744_S6_R1_001.fastq.gz
SUM149_GFP_H3_C_Ct SUM149_GFP_H3_C_Ct.rep1_treat_pileup.bw
20190310_SUM149_GFP_H3_C_Ct_GP6743_S1_R1_001.fastq.gz
SUM149_GFP_H3_C_GFP SUM149_GFP_H3_C_GFP.rep1_treat_pileup.bw
20190310_SUM149_GFP_H3_C_GFP_GP6743_S3_R1_001.fastq.gz
SUM149_GFP_H3_M_Ct SUM149_GFP_H3_M_Ct.rep1_treat_pileup.bw
20190310_SUM149_GFP_H3_M_Ct_GP6743_S2_R1_001.fastq.gz
SUM149_GFP_H3_M_GFP SUM149_GFP_H3_M_GFP.rep1_treat_pileup.bw
20190310_SUM149_GFP_H3_M_GFP_GP6743_S4_R1_001.fastq.gz
HCC1806_sh5_Vehicle_Input NA 20240725_HCC1806_Veh_Input_ZL12286_S168_L008_R1_001.fastq.gz
20240725_HCC1806_Veh_Input_ZL12286_S168_L008_R2_001.fastq.gz
HCC1806_sh5_Vehicle_H3K9me3_rep1 HCC1806_H3K9me3_Veh_rep1.bw
20240725_HCC1806_Veh_K9_1_ZL12286_S152_L008_R1_001.fastq.gz
20240725_HCC1806_Veh_K9_1_ZL12286_S152_L008_R2_001.fastq.gz
HCC1806_sh5_Vehicle_H3K9me3_rep2 HCC1806_H3K9me3_Veh_rep2.bw
20240725_HCC1806_Veh_K9_2_ZL12286_S153_L008_R1_001.fastq.gz
20240725_HCC1806_Veh_K9_2_ZL12286_S153_L008_R2_001.fastq.gz
HCC1806_sh5_Vehicle_H3K36me3_rep1 HCC1806_H3K36m3_Veh_rep1.bw
20240725_HCC1806_Veh_K36_1_ZL12286_S160_L008_R1_001.fastq.gz
20240725_HCC1806_Veh_K36_1_ZL12286_S160_L008_R2_001.fastq.gz
HCC1806_sh5_Vehicle_H3K36me3_rep2 HCC1806_H3K36me3_Veh_rep2.bw
20240725_HCC1806_Veh_K36_2_ZL12286_S161_L008_R1_001.fastq.gz
20240725_HCC1806_Veh_K36_2_ZL12286_S161_L008_R2_001.fastq.gz
HCC1806_sh5_shKDM4C_Input NA 20240725_HCC1806_Dox_Input_ZL12286_S169_L008_R1_001.fastq.gz
20240725_HCC1806_Dox_Input_ZL12286_S169_L008_R2_001.fastq.gz
HCC1806_sh5_shKDM4C_H3K9me3_rep1 HCC1806_H3K9me3_shKDM4C_rep1.bw
20240725_HCC1806_Dox_K9_1_ZL12286_S154_L008_R1_001.fastq.gz
20240725_HCC1806_Dox_K9_1_ZL12286_S154_L008_R2_001.fastq.gz
HCC1806_sh5_shKDM4C_H3K9me3_rep2 HCC1806_H3K9me3_shKDM4C_rep2.bw
20240725_HCC1806_Dox_K9_2_ZL12286_S155_L008_R1_001.fastq.gz
20240725_HCC1806_Dox_K9_2_ZL12286_S155_L008_R2_001.fastq.gz
HCC1806_sh5_shKDM4C_H3K36me3_rep1 HCC1806_H3K36m3_shKDM4C_rep1.bw
20240725_HCC1806_Dox_K36_1_ZL12286_S162_L008_R1_001.fastq.gz
20240725_HCC1806_Dox_K36_1_ZL12286_S162_L008_R2_001.fastq.gz
HCC1806_sh5_shKDM4C_H3K36me3_rep2 HCC1806_H3K36me3_shKDM4C_rep2.bw

20240725_HCC1806_Dox_K36_2_ZL12286_S163_L008_R1_001.fastq.gz
20240725_HCC1806_Dox_K36_2_ZL12286_S163_L008_R2_001.fastq.gz

Genome browser session
(e.g. UCSC)

N/A

Methodology

Replicates

Samples were sequenced without replicates

Sequencing depth

Total reads and mapped reads for each sample are list below:
Sample Total reads Mapped reads
HCC1954_shKDM4C-17_Control_Input 41337134 40508691
HCC1954_shKDM4C-17_Control_K9me3_1 35650438 35254271
HCC1954_shKDM4C-17_Control_K9me3_2 69686476 68650919
HCC1954_shKDM4C-17_Doxy_K9me3_1 52364752 51651492
HCC1954_shKDM4C-17_Doxy_K9me3_2 72186523 71155032
HCC1954_shKDM4C-17_ML324_K9me3_1 59889055 59332877
HCC1954_shKDM4C-17_ML324_K9me3_2 75425157 74324524
HCC1954_shKDM4C-17_Control_K36me3_1 64032303 63508870
HCC1954_shKDM4C-17_Control_K36me3_2 63477308 62935996
HCC1954_shKDM4C-17_Doxy_K36me3_1 63942137 63470769
HCC1954_shKDM4C-17_Doxy_K36me3_2 66498082 65932361
HCC1954_shKDM4C-17_ML324_K36me3_1 67186223 66616821
HCC1954_shKDM4C-17_ML324_K36me3_2 61683058 61113788
HCC1954_shKDM4C-17_Control_KDM4C_1 64503732 63744575
HCC1954_shKDM4C-17_Control_KDM4C_2 47495897 46542830
HCC1954_shKDM4C-17_Control_H3K4me3 49332574 48392176
HCC1954_shKDM4C-17_Doxy_H3K4me3 54622248 53607668
HCC1954_shKDM4C-17_ML324_H3K4me3 55656966 54596743
SUM149_shKDM4C-17_Control_Input_3 49322784 48900869
SUM149_shKDM4C-17_Control_K9me3_1 52768119 51723420
SUM149_shKDM4C-17_Control_K9me3_2 34518124 33530615
SUM149_shKDM4C-17_Control_K9me3_3 45920035 44266460
SUM149_shKDM4C-17_Control_K9me3_4 77964643 76869055
SUM149_shKDM4C-17_Control_K9me3_5 33748977 33145200
SUM149_shKDM4C-17_Doxy_K9me3_1 53887540 52982582
SUM149_shKDM4C-17_Doxy_K9me3_2 5817497 5744053
SUM149_shKDM4C-17_Doxy_K9me3_3 57675016 56702389
SUM149_shKDM4C-17_ML324_K9me3_1 43647241 42626233
SUM149_shKDM4C-17_ML324_K9me3_2 58205744 57621055
SUM149_shKDM4C-17_ML324_K9me3_3 48038238 47401625
SUM149_shKDM4C-17_Control_K36me3_1 46560838 46174173
SUM149_shKDM4C-17_Control_K36me3_2 32526373 31953022
SUM149_shKDM4C-17_Control_K36me3_3 63458026 63046561
SUM149_shKDM4C-17_Control_K36me3_4 62590933 61985700
SUM149_shKDM4C-17_Control_K36me3_5 38767418 37693203
SUM149_shKDM4C-17_Doxy_K36me3_1 59718811 59296737
SUM149_shKDM4C-17_Doxy_K36me3_2 52225986 51867463
SUM149_shKDM4C-17_Doxy_K36me3_3 57093783 55559936
SUM149_shKDM4C-17_ML324_K36me3_1 44656702 44268443
SUM149_shKDM4C-17_ML324_K36me3_2 49668179 49313573
SUM149_shKDM4C-17_ML324_K36me3_3 66780988 66230959
SUM149_shKDM4C-17_ML324_K36me3_4 61884460 59909565
SUM149_shKDM4C-17_Control_H3K4me3 55539815 54380325
SUM149_shKDM4C-17_Doxy_H3K4me3 52797037 51742385
SUM149_shKDM4C-17_ML324_H3K4me3 42542595 41706550
SUM149_shKDM4C-17_Control_KDM4C_1 51952956 51242251
SUM149_shKDM4C-17_Control_KDM4C_2 39709747 38646767
SUM149_shKDM4C-17_Control_KDM4C_3 53758843 52748982
SUM149_shKDM4C-17_Control_KDM4C_4 57085372 55975068
SUM149_Parental_Control_K27me3 33706284 33076818
HCC70_Parental_Untreated_INPUT 71399350 70798465
HCC70_Parental_Untreated_KDM4C 49448188 48781400
MCF7_Parental_Untreated_KDM4C 51791943 51152407
MCF7_Parental_Untreated_K9me3 55206835 54159768
MCF7_Parental_Untreated_K36me3 56327253 55796610
HCC2157_Parental_Untreated_KDM4C 54765134 53482859
T47D_shKDM4C-17_Control_INPUT 66982316 66471712
T47D_shKDM4C-17_Control_KDM4C 56749252 55527416
T47D_shKDM4C-17_Control_K9me3_1 45236822 44208791
T47D_shKDM4C-17_Control_K36me3_1 54557802 54130497
SUM149_Parental_Control_INPUT 39210940 38718439
SUM149_Parental_ML324_INPUT 34839122 34358586
SUM149_Parental_Control_CTSL 59313832 58638940
SUM149_Parental_ML324_CTSL 65554888 64930215

SUM149_ML324-R_Control_CTSL 57213657 56649638
SUM149_ML324-R_ML324_CTSL 61397786 60799339
SUM149_Parental_Control_GRHL2 42969183 42448097
SUM149_Parental_ML324_GRHL2 34808283 34394105
SUM149_Parental_Control_INPUT 39210940 38718439
HCC1954_Parental_Control_CTSL 35486472 35129813
HCC1954_Parental_Control_GRHL2 37744481 37362500
SUM149_C_KDM4A 29381502 29162002
SUM149_C_KDM4B 31531231 31238012
SUM149_C_KDM4C 27988857 27759865
SUM149_M_KDM4A 27378552 27148471
SUM149_M_KDM4B 35639193 35319510
SUM149_M_KDM4C 37197182 36900802
SUM149_sgGRHL2_C_CTSL 45943203 45544150
SUM149_sgScramble_C_CTSL 46167863 45737817
SUM149_V5_H3_C_Ct 37016508 36623119
SUM149_V5_H3_C_V5 36402115 36022268
SUM149_V5_H3_M_Ct 35471500 35100958
SUM149_V5_H3_M_V5 37951278 37567682
SUM149_GFP_H3_C_Ct 40817278 40266728
SUM149_GFP_H3_C_GFP 34433880 33954700
SUM149_GFP_H3_M_Ct 33657105 33185044
SUM149_GFP_H3_M_GFP 27536713 27151567
HCC1806_sh5_Vehicle_Input 94096888 94075590
HCC1806_sh5_Vehicle_H3K9me3_rep1 84424411 84476918
HCC1806_sh5_Vehicle_H3K9me3_rep2 88802017 88854067
HCC1806_sh5_Vehicle_H3K36me3_rep1 72683147 72701115
HCC1806_sh5_Vehicle_H3K36me3_rep2 66996566 67012625
HCC1806_sh5_shKDM4C_Input 82387126 82407395
HCC1806_sh5_shKDM4C_H3K9me3_rep1 100552214 100617843
HCC1806_sh5_shKDM4C_H3K9me3_rep2 99835844 99905731
HCC1806_sh5_shKDM4C_H3K36me3_rep1 95469296 95497613
HCC1806_sh5_shKDM4C_H3K36me3_rep2 95089343 95114661

Antibodies

Rabbit polyclonal KDM4C anitbody Novus Biologicals Cat# NBP1-49600, RRID:AB_10011699
Goat polyclonal Anti-cathepsin L antibody Novus Biologicals Cat# AF952, RRID:AB_355737
Rabbit polyclonal Anti-GRHL2 antibody Sigma-Aldrich Cat# HPA004820, RRID:AB_1857928
Rabbit polyclonal Anti-Histone H3 antibody (C-terminus) Abcam Cat# ab1791, RRID:AB_302613
Rabbit polyclonal Anti-GFP antibody Novus Biologicals Cat# NB600-308, RRID:AB_10003058
Rabbit polyclonal Anti-V5 Tag antibody Novus Biologicals Cat# NB600-381, RRID:AB_10001084
Rabbit polyclonal Anti-Histone H3 (tri methyl K9) antibody Abcam Cat# ab8898, RRID:AB_306848
Rabbit polyclonal Anti-Histone H3 (tri methyl K36) antibody Abcam Cat# ab9050, RRID:AB_306966
Rabbit polyclonal Anti-Histone H3 (tri methyl K4) antibody Abcam Cat# ab8580, RRID:AB_306649
Rabbit polyclonal Anti-Histone H3 (acetylation K27) antibody Diagenode C15410196, RRID:AB_2637079 (ChIP-seq)
Rabbit polyclonal Anti-JMJD2A antibody Bethyl Laboratories Cat# A300-861A, RRID:AB_609461
Rabbit polyclonal Anti-JMJD2B antibody Active Motif Cat# 61222, RRID:AB_2615033

Peak calling parameters

Peak calling is used the Model-Based Analysis of ChIP-seq 2 (MACS v2.1.2), with a q-value (FDR) threshold of 0.01.

Data quality

We evaluated multiple quality control criteria based on alignment information and peak quality: (i) sequence quality score; (ii) uniquely mappable reads (reads that can only map to one location in the genome); (iii) uniquely mappable locations (locations that can only be mapped by at least one read); (iv) peak overlap with Velcro regions, a comprehensive set of locations – also called consensus signal artifact regions – in the genome that have anomalous, unstructured high signal or read counts in next-generation sequencing experiments independent of cell line and of type of experiment; (v) number of total peaks (the minimum required was 1,000); (vi) high-confidence peaks (the number of peaks that are tenfold enriched over background); (vii) percentage overlap with known DHS sites derived from the ENCODE Project (the minimum required to meet the threshold was 80%); and (viii) peak conservation (a measure of sequence similarity across species based on the hypothesis that conserved sequences are more likely to be functional).

Software

CHIPS(https://github.com/liulab-dfci/CHIPS) for QC; BWA(http://bio-bwa.sourceforge.net/) for mapping; MACS2(http://liulab.dfci.harvard.edu/MACS/) for peak calling. ChIP-seq peak calling was performed using CHIPs pipeline and analyzed by Seqplots (v.1.12.0), BEDtools (v.2.30.0), deepTools (v.3.5.0).

Flow Cytometry

Plots

Confirm that:

☒ The axis labels state the marker and fluorochrome used (e.g. CD4-FITC).

☒ The axis scales are clearly visible. Include numbers along axes only for bottom left plot of group (a 'group' is an analysis of identical markers).

☒ All plots are contour plots with outliers or pseudocolor plots.

☒ A numerical value for number of cells or percentage (with statistics) is provided.

Methodology

Sample preparation	Reactive oxygen species levels were assessed using CellROX Green (Fisher Scientific) and CTSL Magic Red Assay Kit (MyBiosource) following manufacturer’s protocol. Briefly, 2x10e5 cells seeded in 6 well plates and treated with DMSO, 1µg/ml doxycycline or 1 µM QC6352 for 5 days. For flow cytometry, cells were first digested and then stained with 200µl PBS solution containing 1x magic red and 1x CellROX green for 30 minutes in 37°C. Stained cells were dissociated to single cells and resuspended in 300 µl PBS and analyzed on BD LSRFortessa™ Cell Analyzer with FITC and PE-Texas Red channel. An unstained sample was used as negative control.
Instrument	BD LSRFortessa™ Cell Analyzer was used for flow cytometric analysis.
Software	BD FACSDiva™ Software was used to data collection and FlowJo v10.10 was used for data analysis.
Cell population abundance	Gates were conservative and consistent across samples to ensure purity within each independent experiment. We collected at lease 10,000 cells per condition.
Gating strategy	FSC/SSC-area was used to select live cells and discriminate doublets. No further gating strategy was applied for the fluorescence analysis as statistic comparison was performed based on geometric mean of intensity merging from three independent experiments.

☒ Tick this box to confirm that a figure exemplifying the gating strategy is provided in the Supplementary Information.
