# Supplementary figures and images for: KDM4C inhibition blocks tumor growth in basal breast cancer by promoting cathepsin L-mediated histone H3 cleavage
Source: Nat Genet. 2025 Jun 2;57(6):1463–77. doi: 10.1038/s41588-025-02197-z (PMC12165855; doi:10.1038/s41588-025-02197-z)

Fig. 2 uncropped blots

Fig. 2d

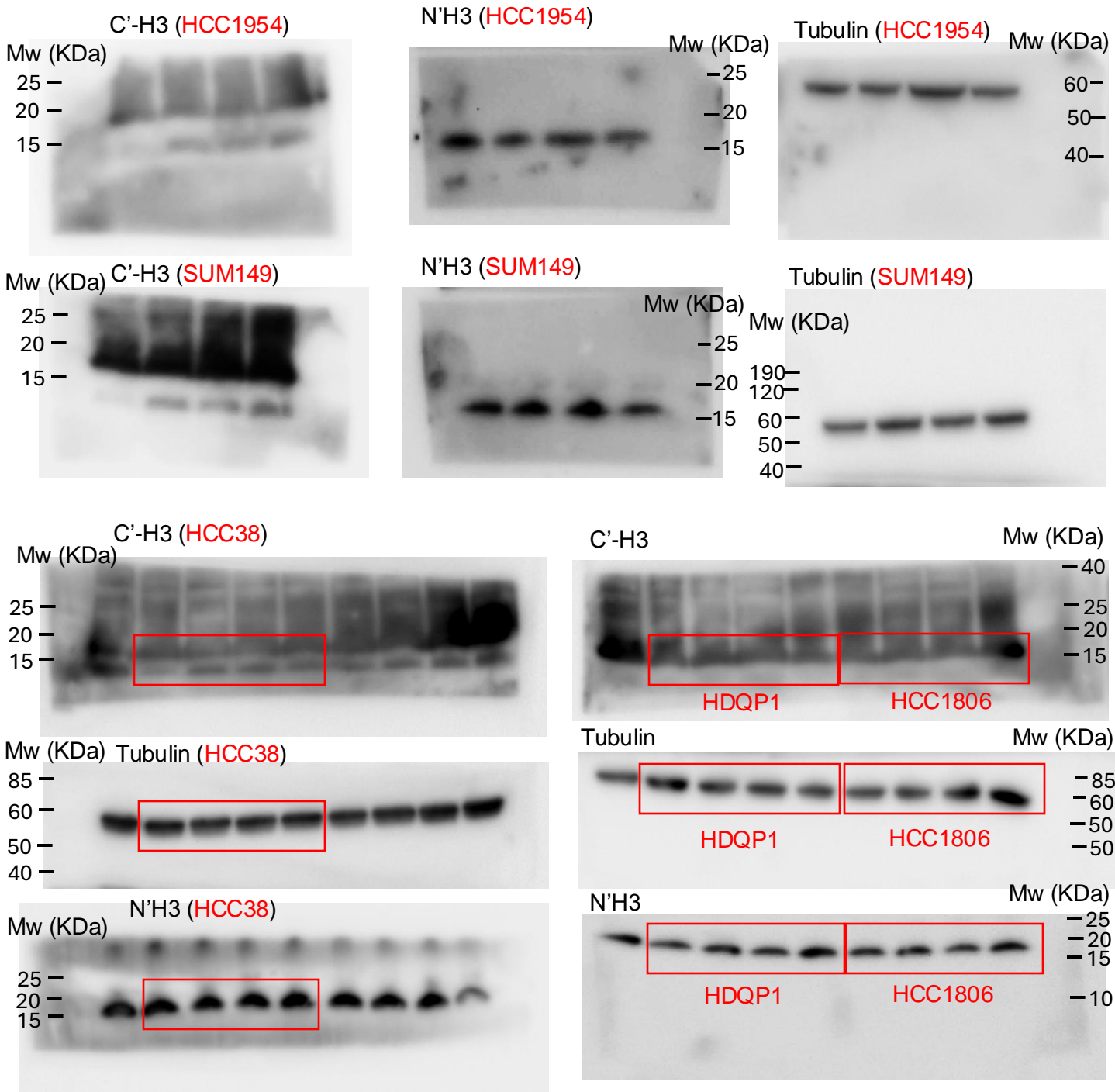

Supplement: Supplementary file 11 — Unprocessed western blots. [file 41588_2025_2197_MOESM11_ESM.pdf]

Fig. 3 uncropped blots

Fig. 3a

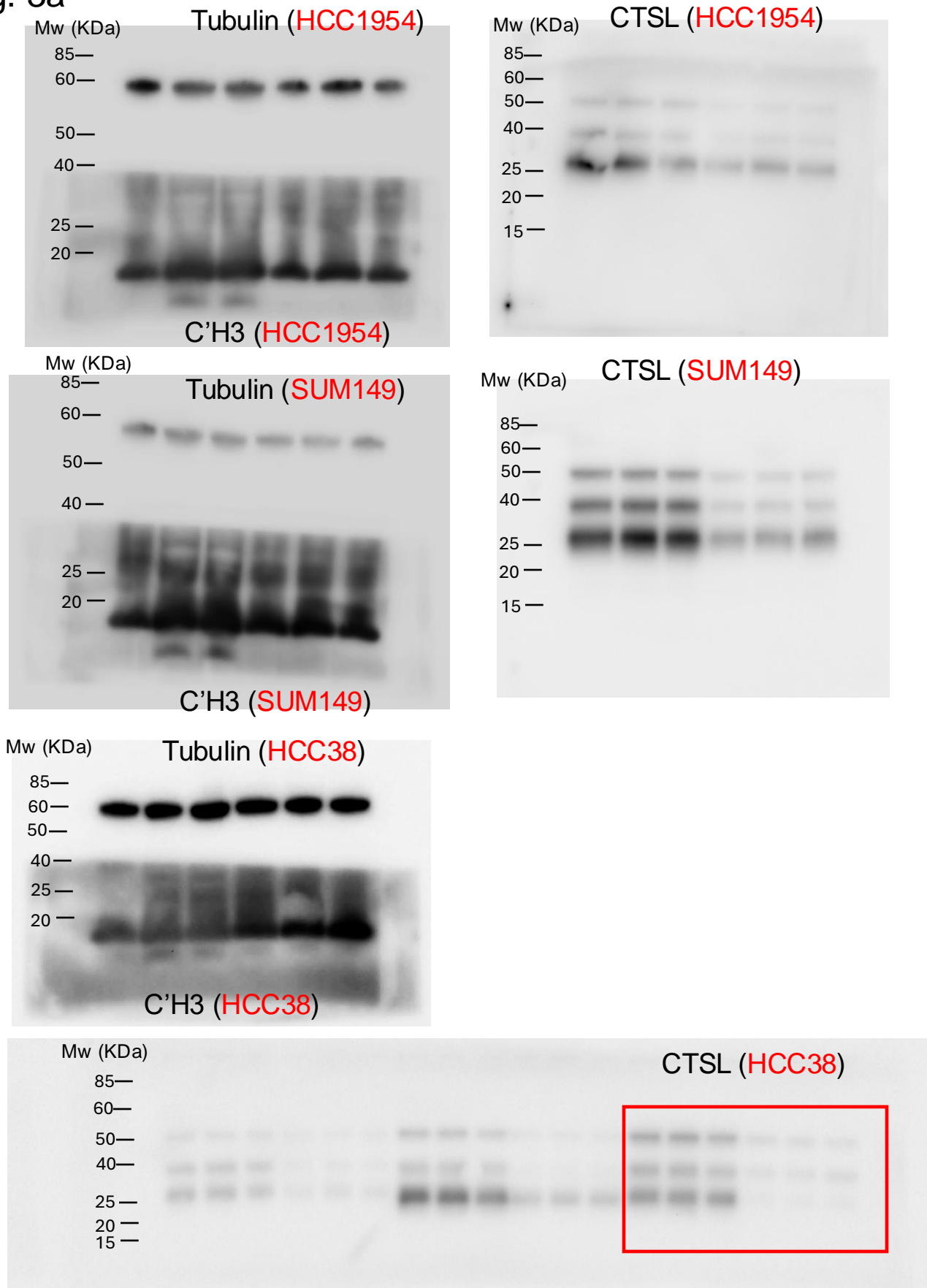

Supplement: Supplementary file 12 — Unprocessed western blots. [file 41588_2025_2197_MOESM12_ESM.pdf]

Fig. 5 Uncropped blots

Fig. 5i

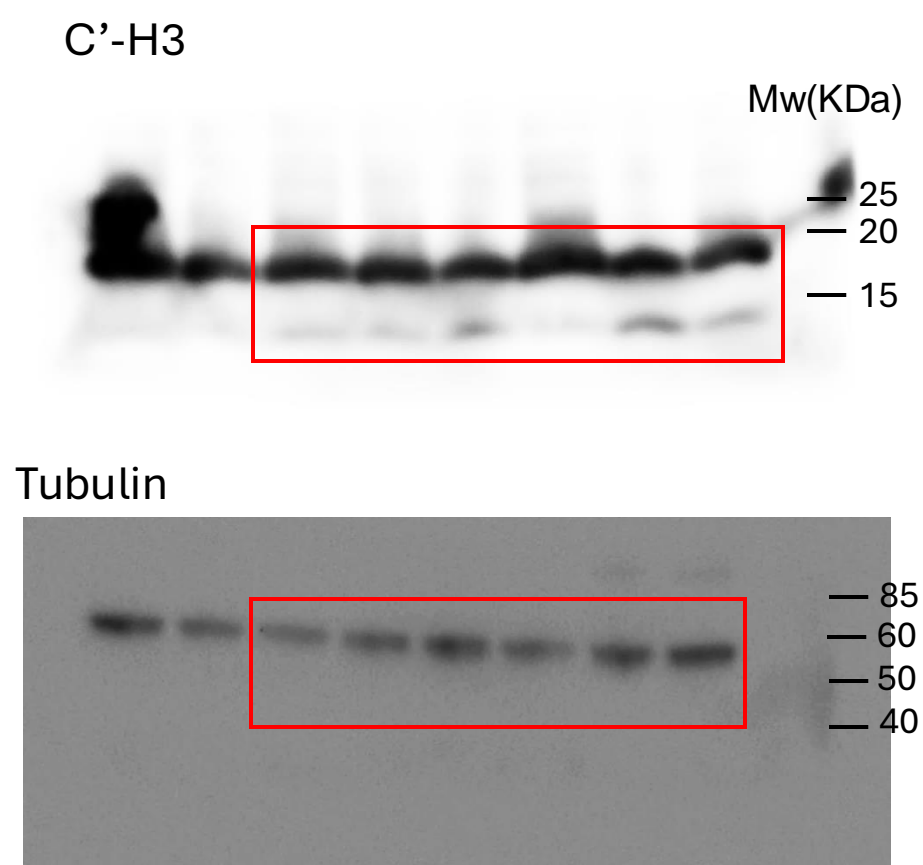

Supplement: Supplementary file 14 — Unprocessed western blots. [file 41588_2025_2197_MOESM14_ESM.pdf]

Fig. 6 Uncropped blots

Fig. 6a

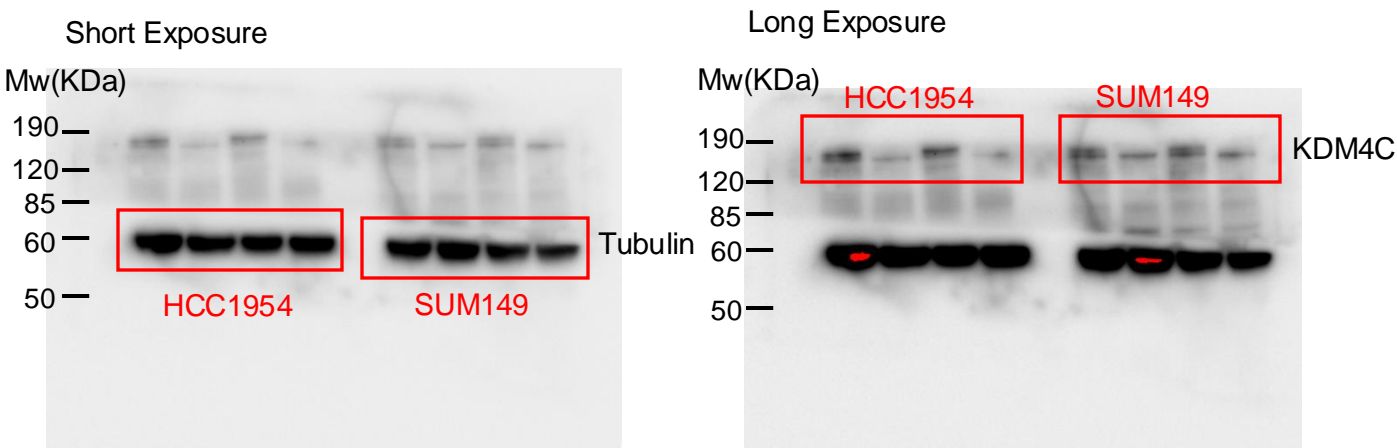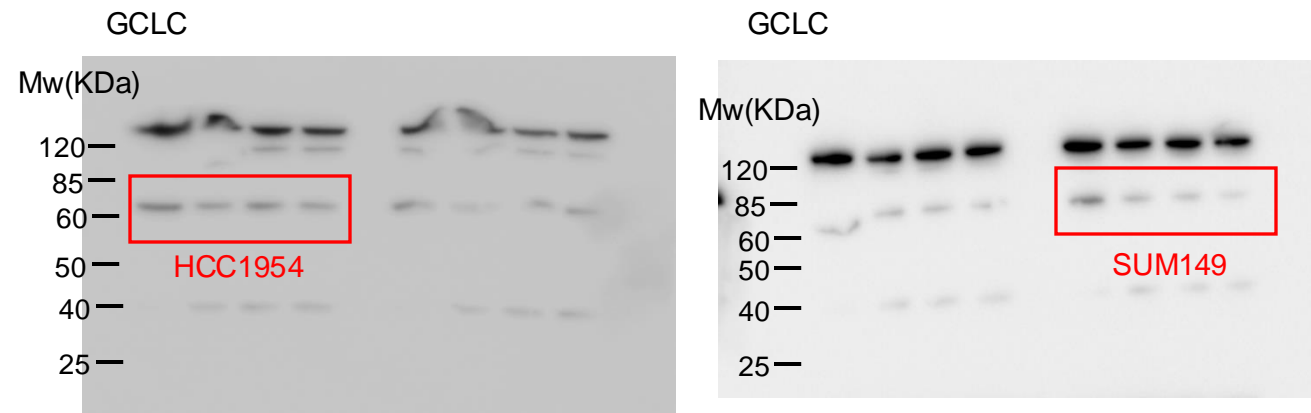

GCLC was run on different gels using the same lysates

Supplement: Supplementary file 15 — Unprocessed western blots. [file 41588_2025_2197_MOESM15_ESM.pdf]

Extended Data Fig. 4 Uncropped blots

Extended Data Fig. 4j

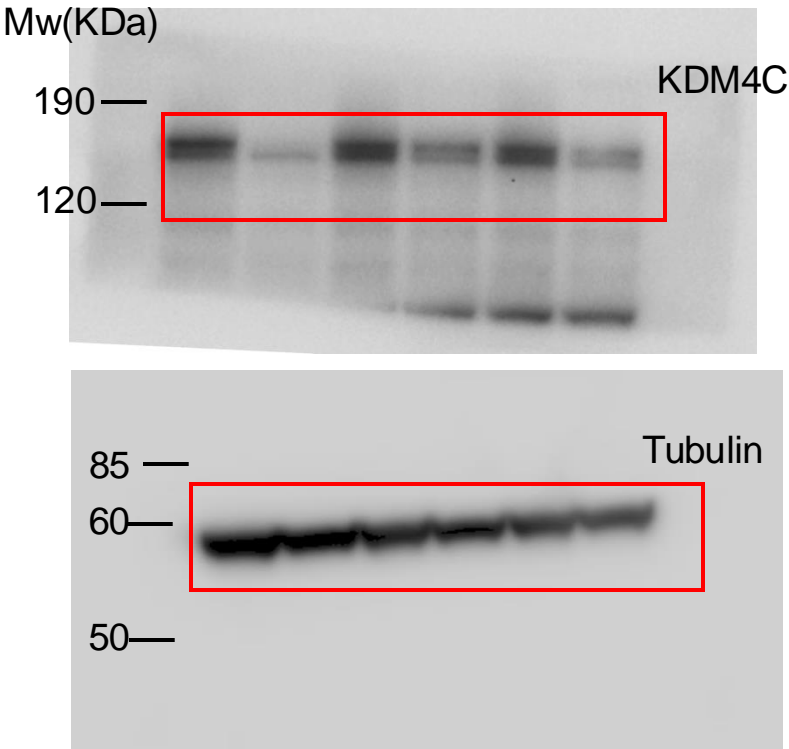

Supplement: Supplementary file 28 — Unprocessed western blots. [file 41588_2025_2197_MOESM28_ESM.pdf]
