# Supplementary material for: KDM4C inhibition blocks tumor growth in basal breast cancer by promoting cathepsin L-mediated histone H3 cleavage
Source: Nat Genet. 2025 Jun 2;57(6):1463–77. doi: 10.1038/s41588-025-02197-z (PMC12165855; doi:10.1038/s41588-025-02197-z)

Fig. 4 Uncropped blots

Fig. 4c (Left panel)

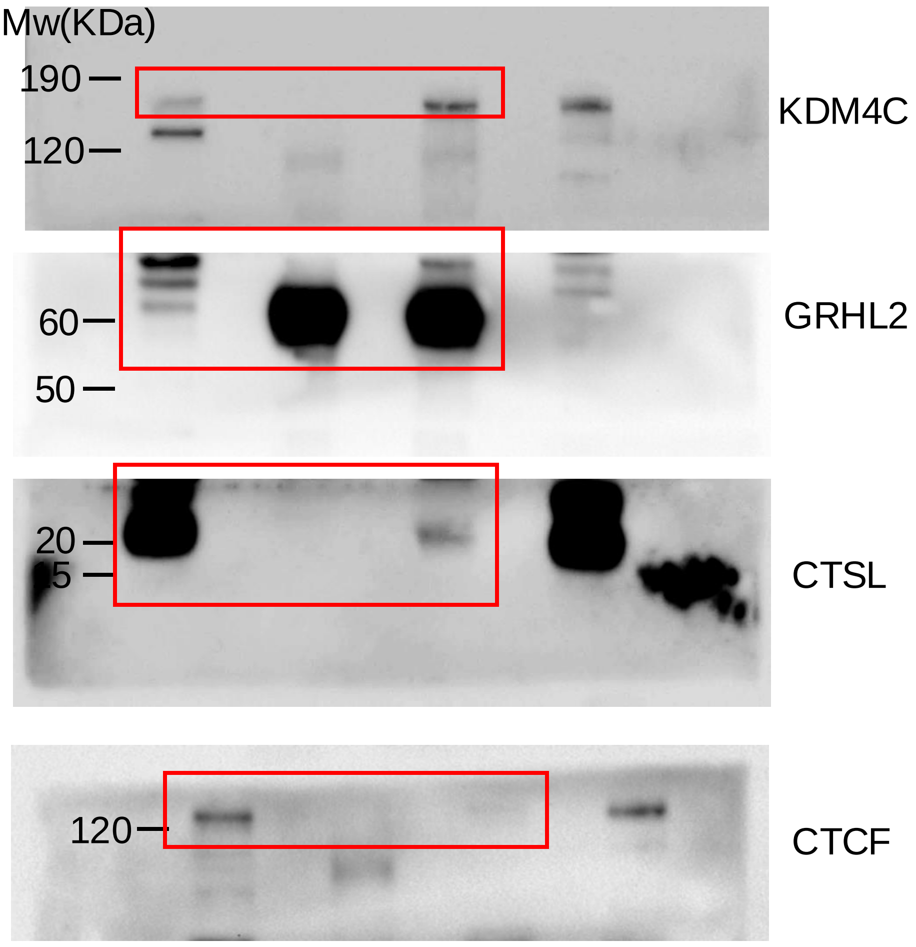

Fig. 4 Uncropped blots

Fig. 4c (Middle panel)

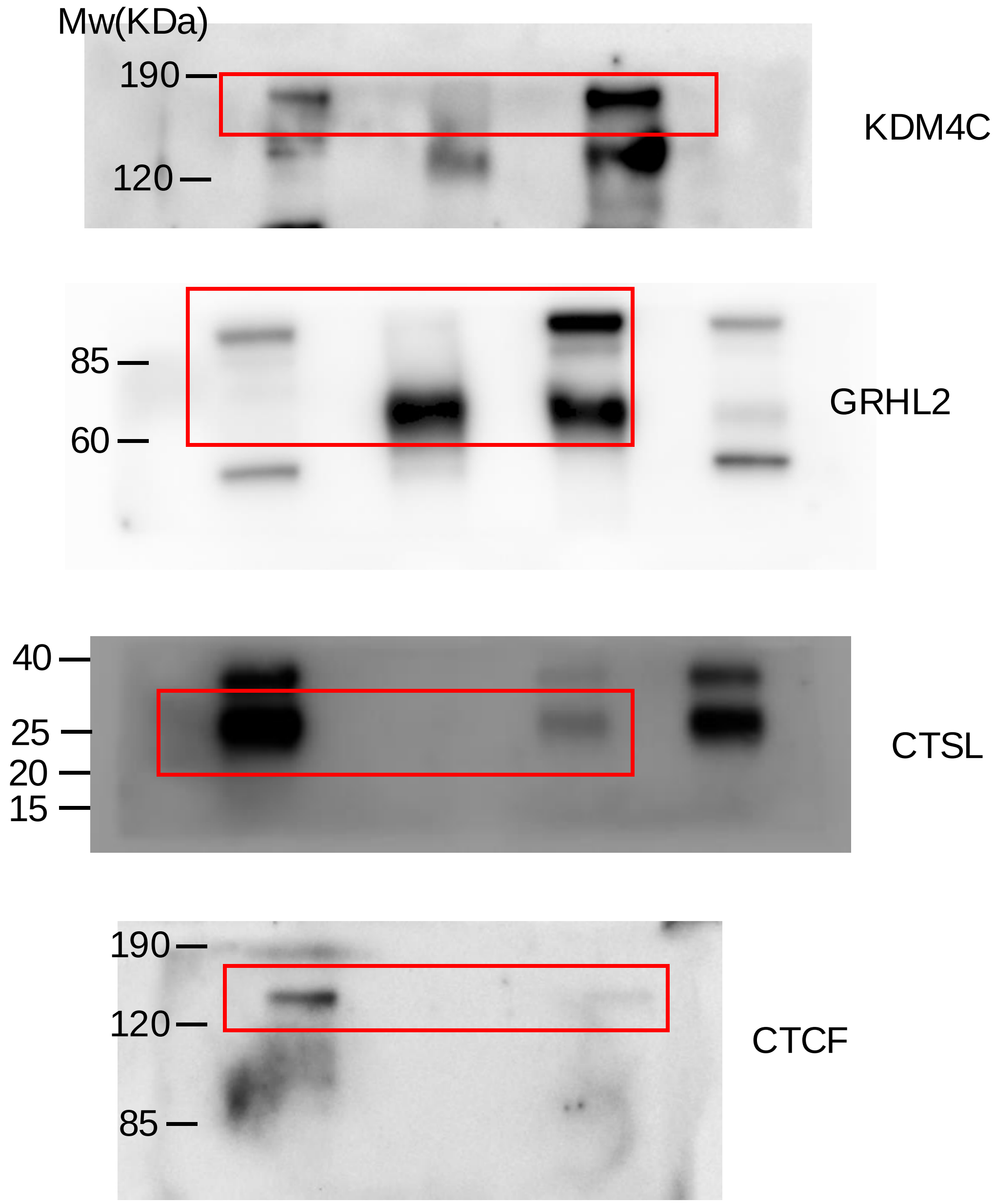

Fig. 4 Uncropped blots

Fig. 4c (Right panel)

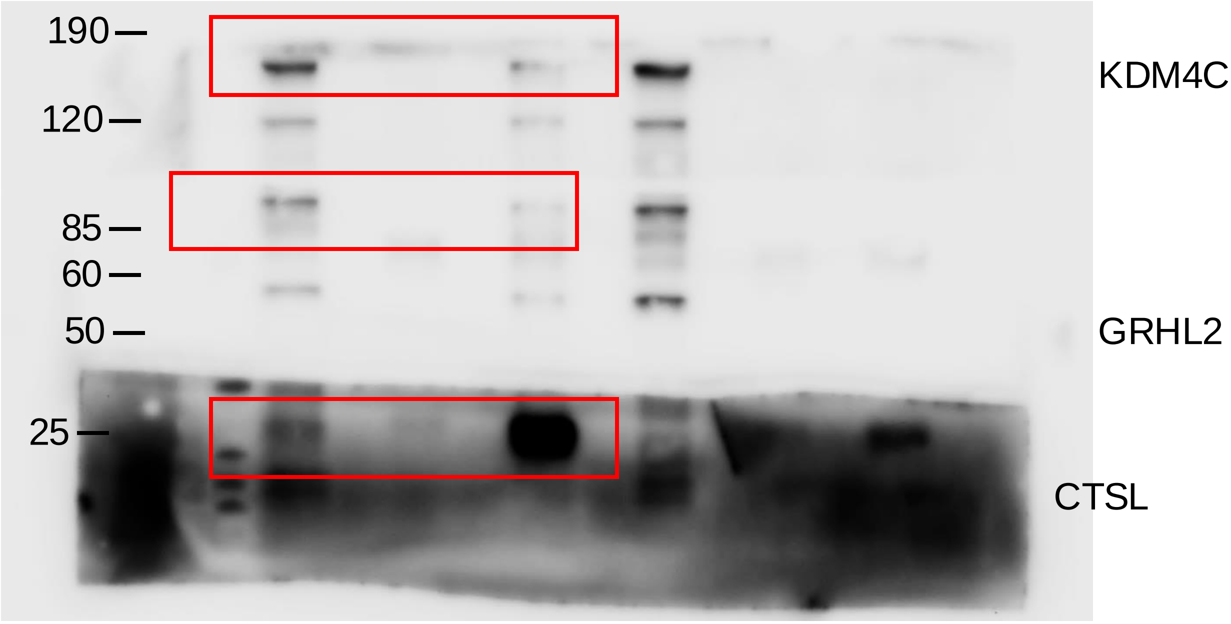

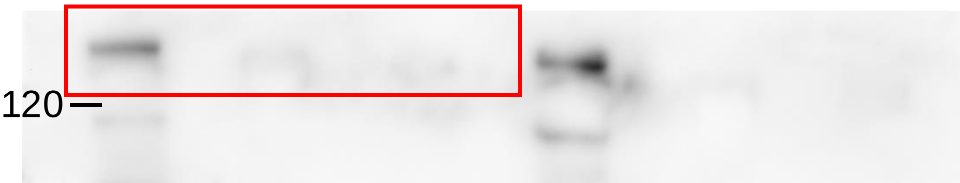

Fig. 4 Uncropped blots

Fig. 4h

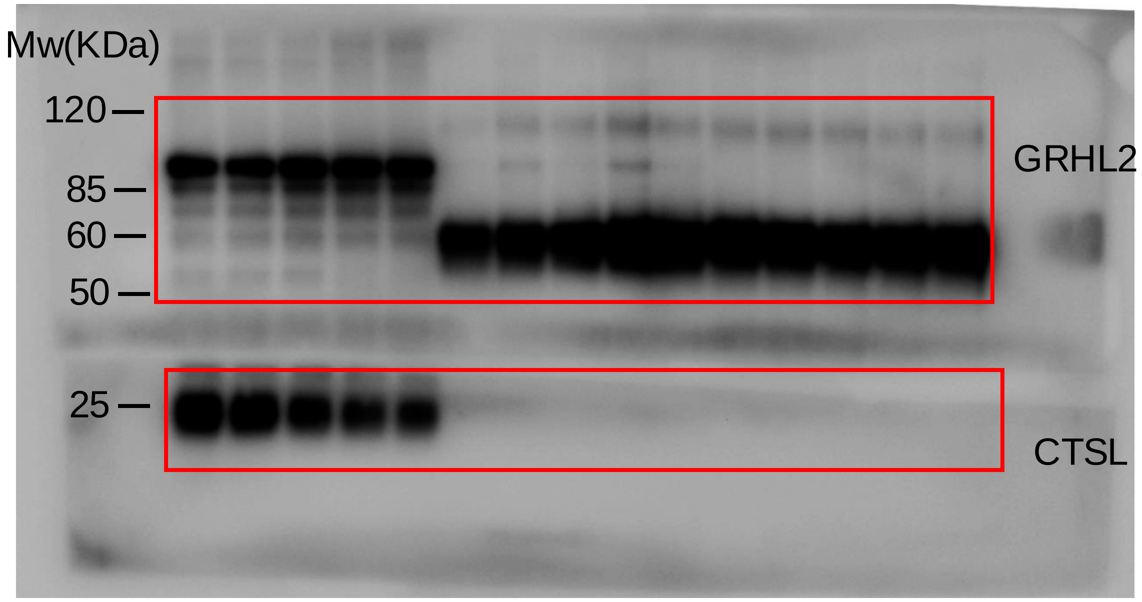

Fig. 4j

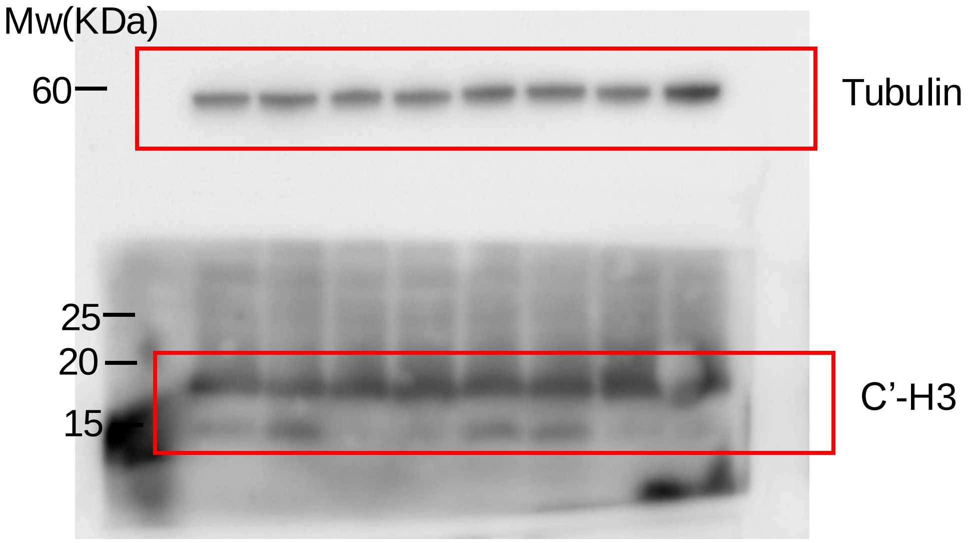

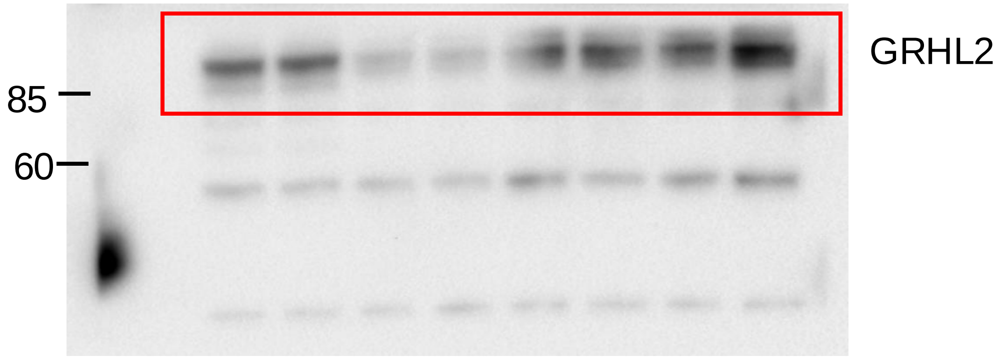

Supplement: Supplementary file 13 — Unprocessed western blots. [file 41588_2025_2197_MOESM13_ESM.pdf]
