# Supplementary material for: KDM4C inhibition blocks tumor growth in basal breast cancer by promoting cathepsin L-mediated histone H3 cleavage
Source: Nat Genet. 2025 Jun 2;57(6):1463–77. doi: 10.1038/s41588-025-02197-z (PMC12165855; doi:10.1038/s41588-025-02197-z)

Extended Data Fig. 2 Uncropped blots

Extended Data Fig. 2b

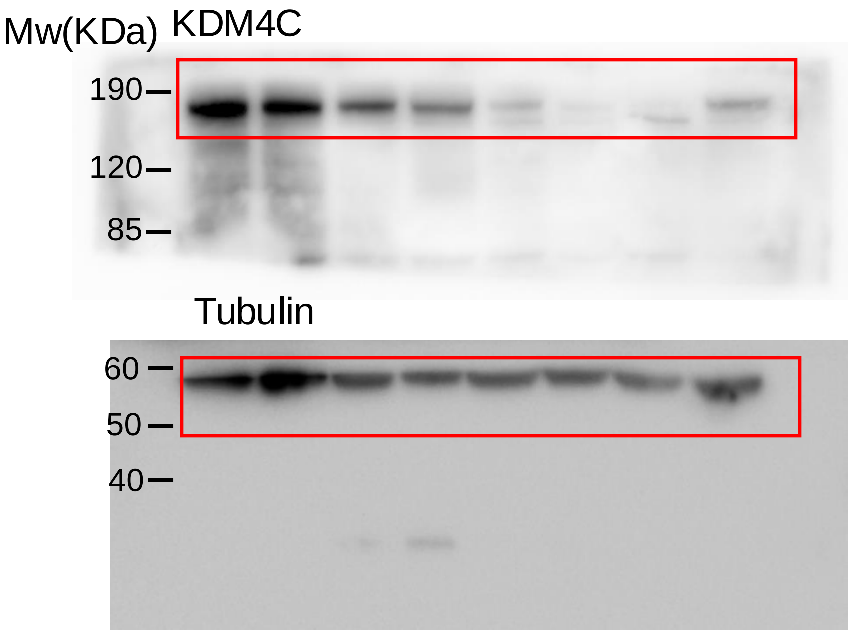

Extended Data Fig. 2 Uncropped blots

Extended Data Fig. 2c

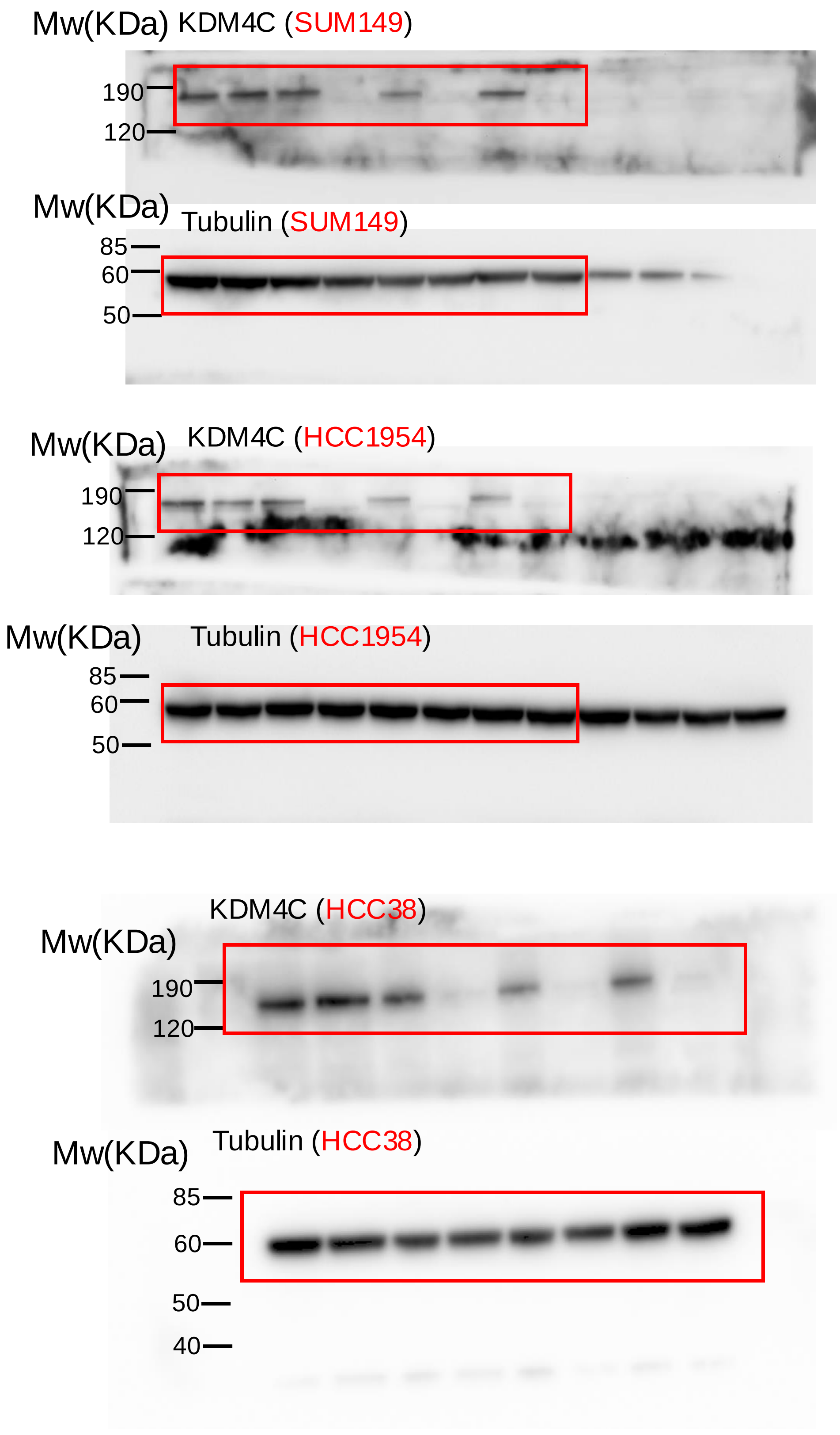

Extended Data Fig. 2 Uncropped blots

Extended Data Fig. 2c (Continued)

Mw(KDa) KDM4C (HDQP1)

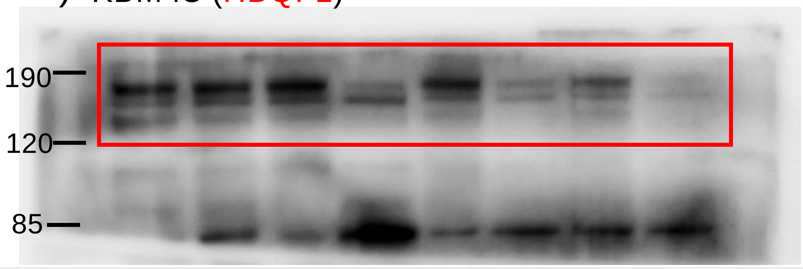

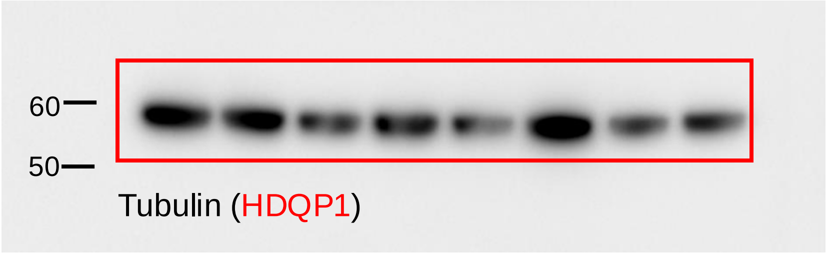

Mw(KDa) KDM4C (HCC1806)

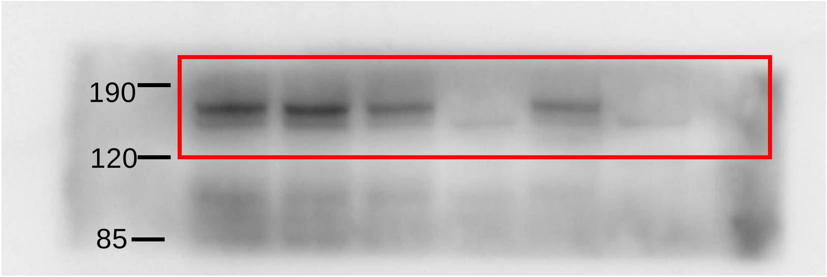

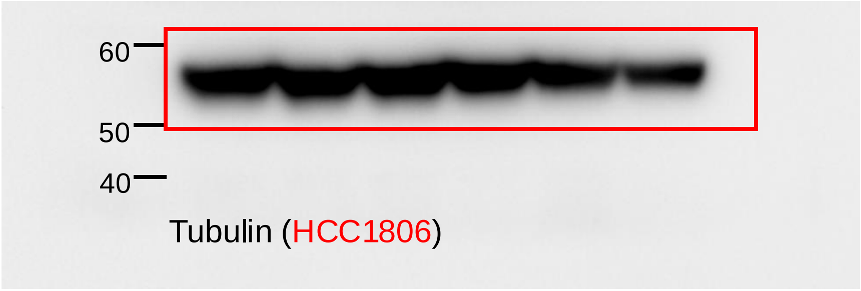

Supplement: Supplementary file 26 — Unprocessed western blots. [file 41588_2025_2197_MOESM26_ESM.pdf]
