# Supplementary material for: KDM4C inhibition blocks tumor growth in basal breast cancer by promoting cathepsin L-mediated histone H3 cleavage
Source: Nat Genet. 2025 Jun 2;57(6):1463–77. doi: 10.1038/s41588-025-02197-z (PMC12165855; doi:10.1038/s41588-025-02197-z)

Extended Data Fig. 6 Uncropped blots

Extended Data Fig. 6a

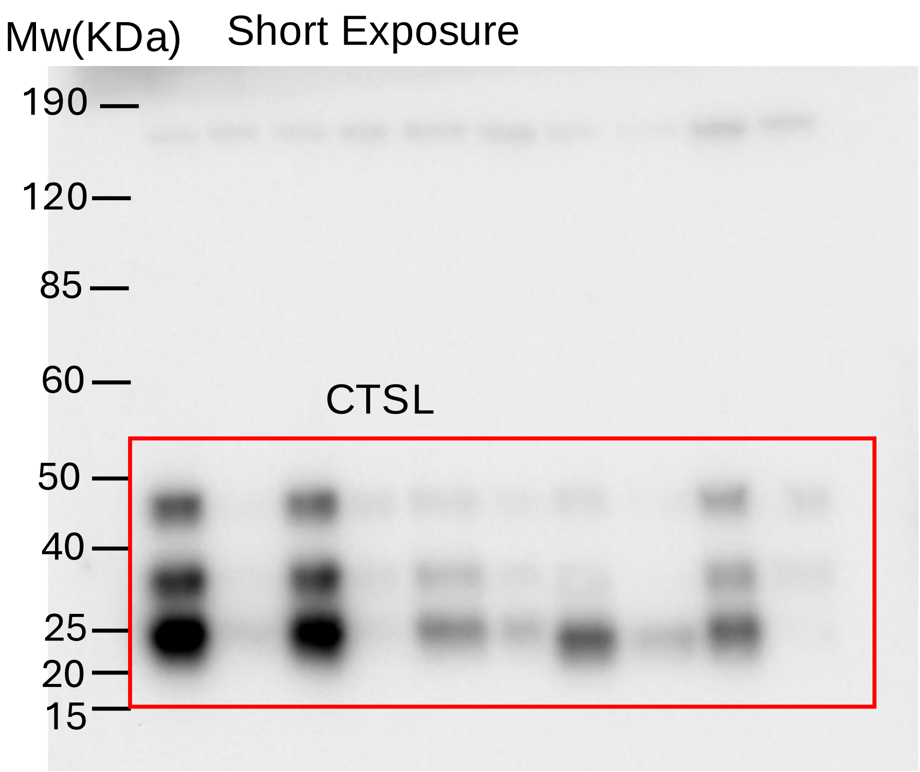

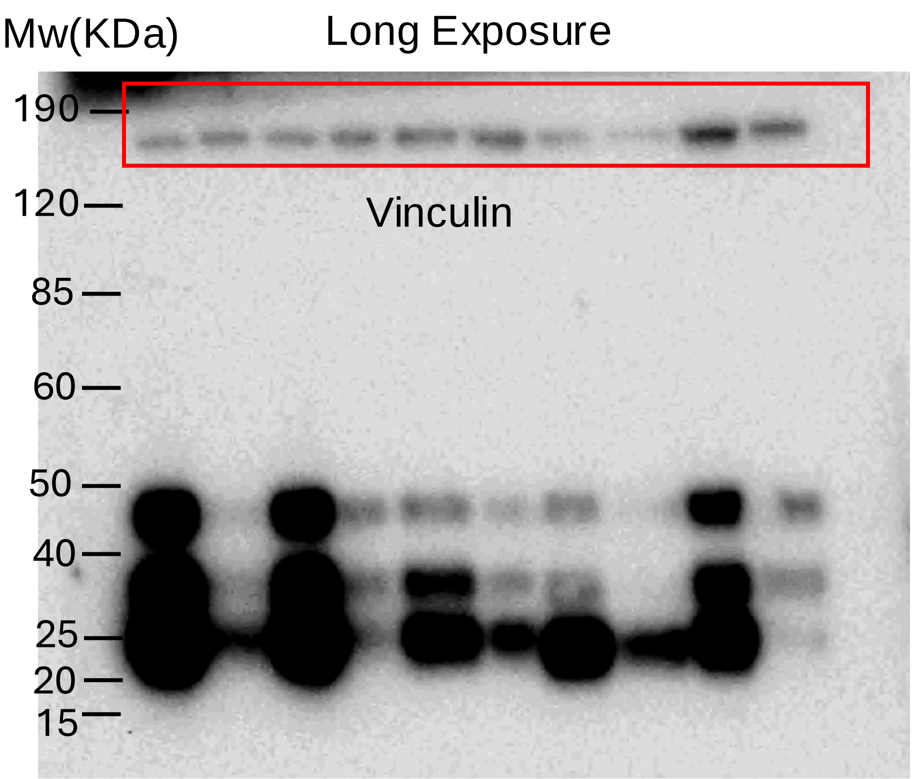

Extended Data Fig. 6c

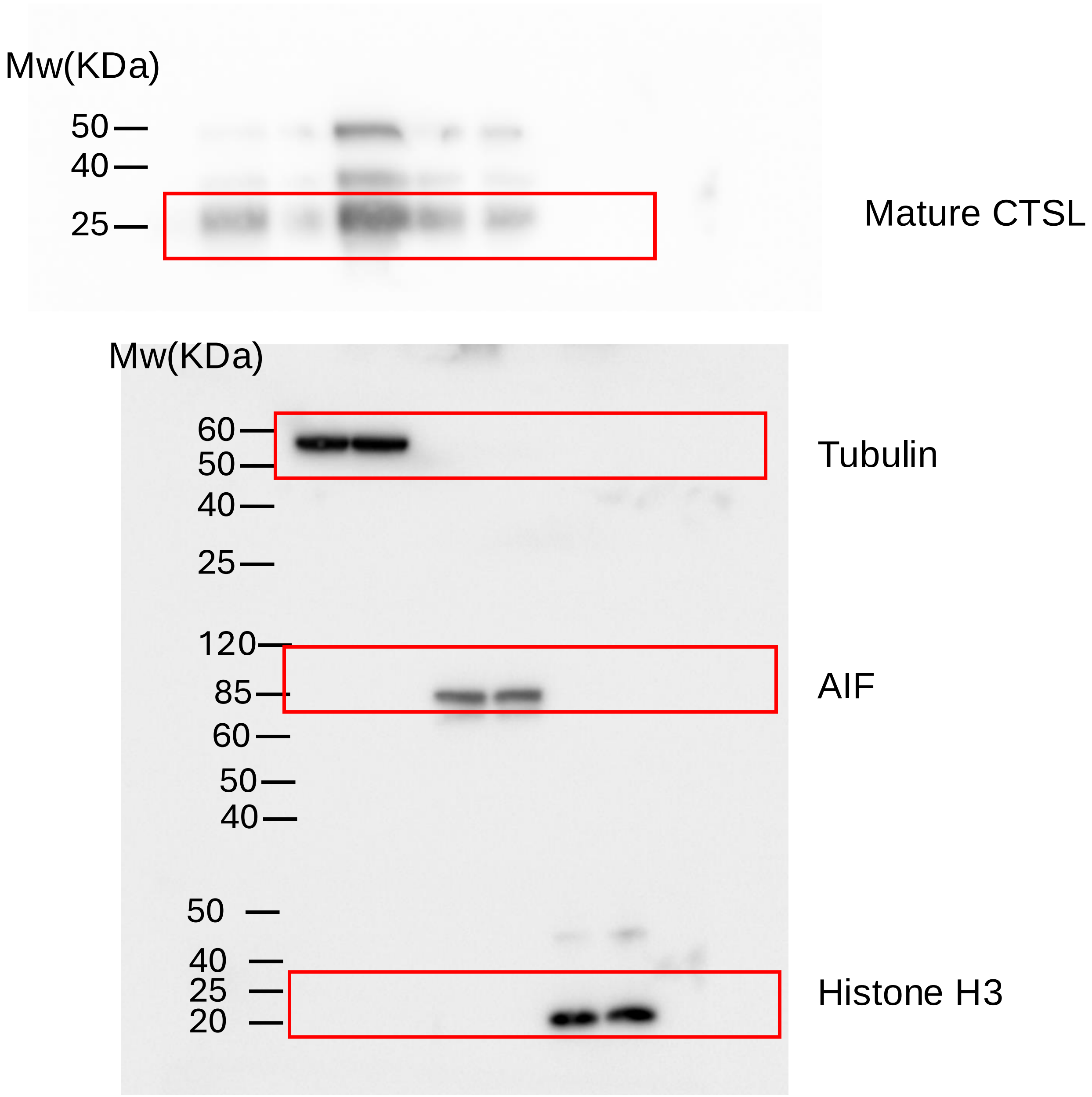

Cell fraction markers were run on separate gels using the same lysates

Extended Data Fig. 6 Uncropped blots

Extended Data Fig. 6e

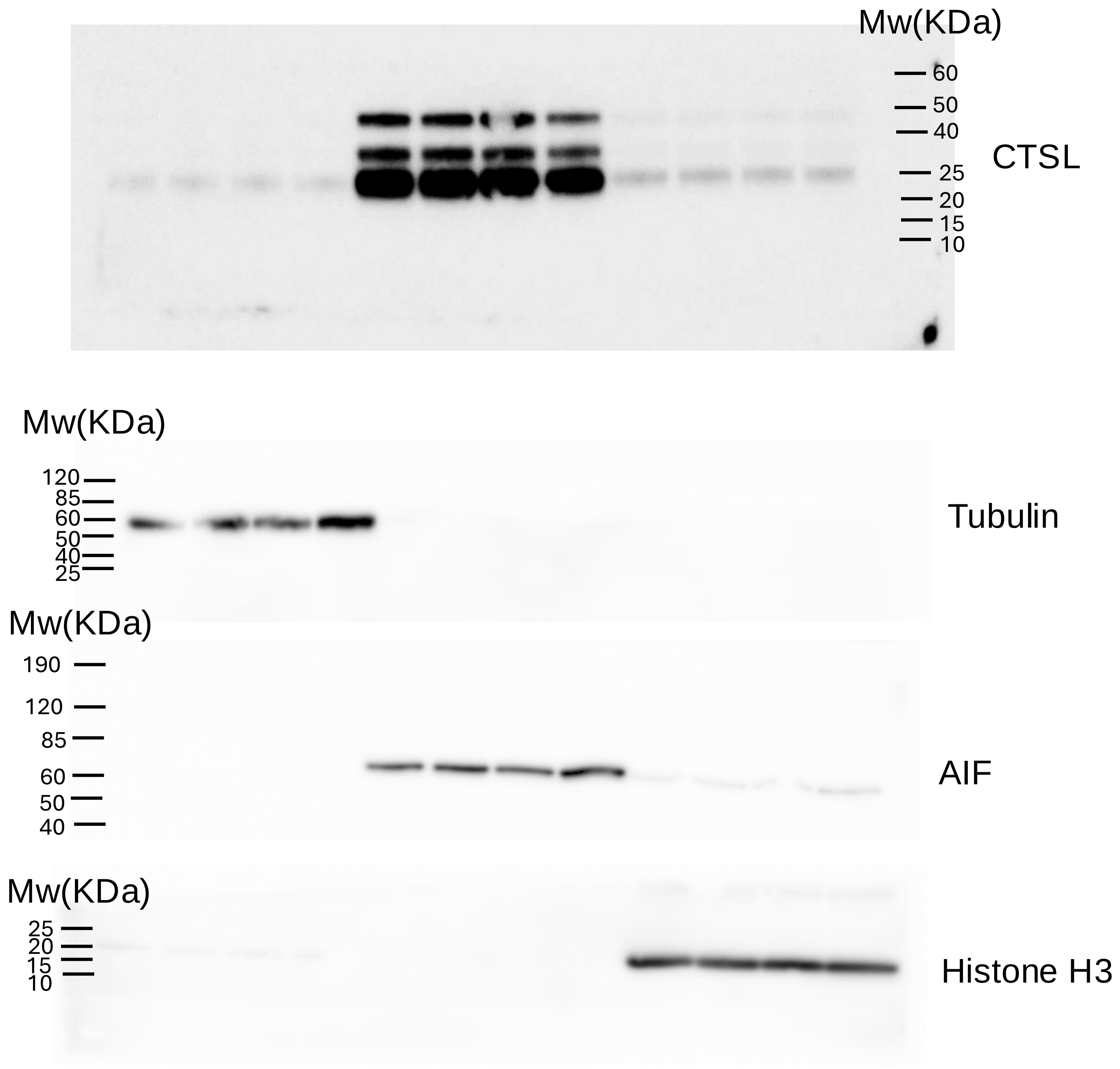

Cell fraction markers were run on separate gels using the same lysates

Extended Data Fig. 7 Uncropped blots

Extended Data Fig. 6f

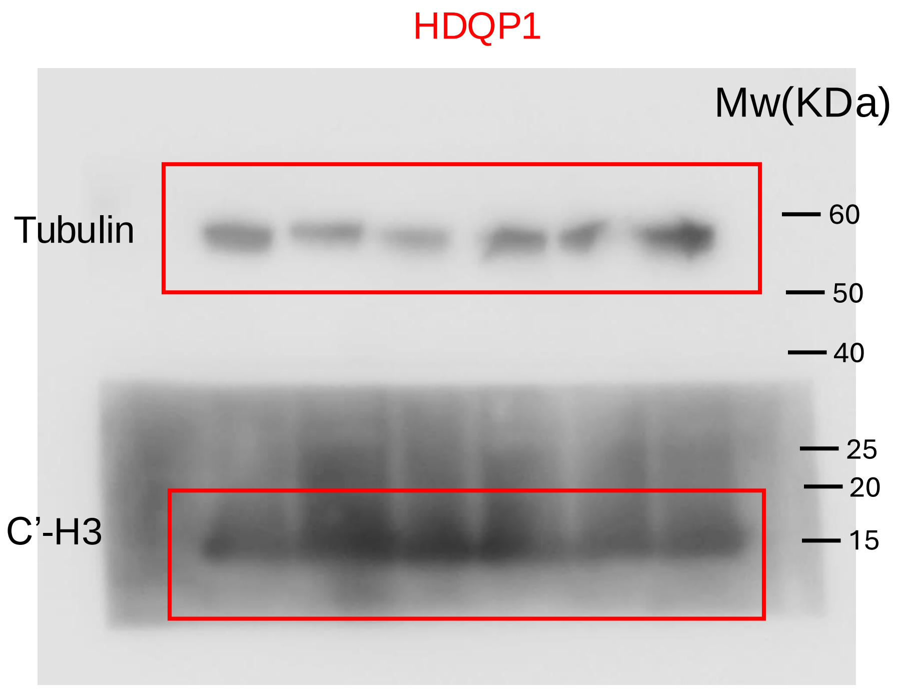

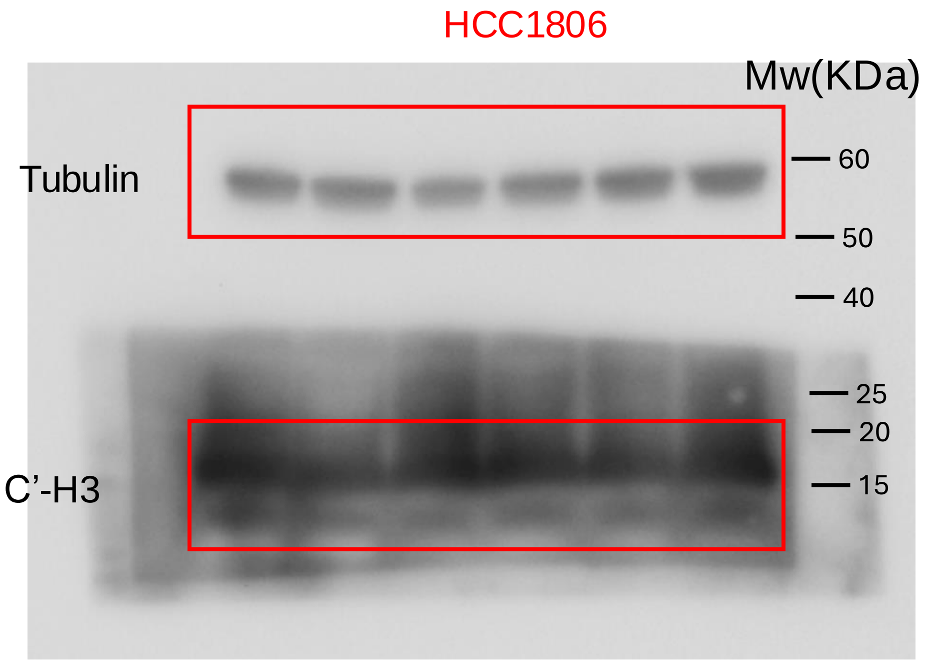

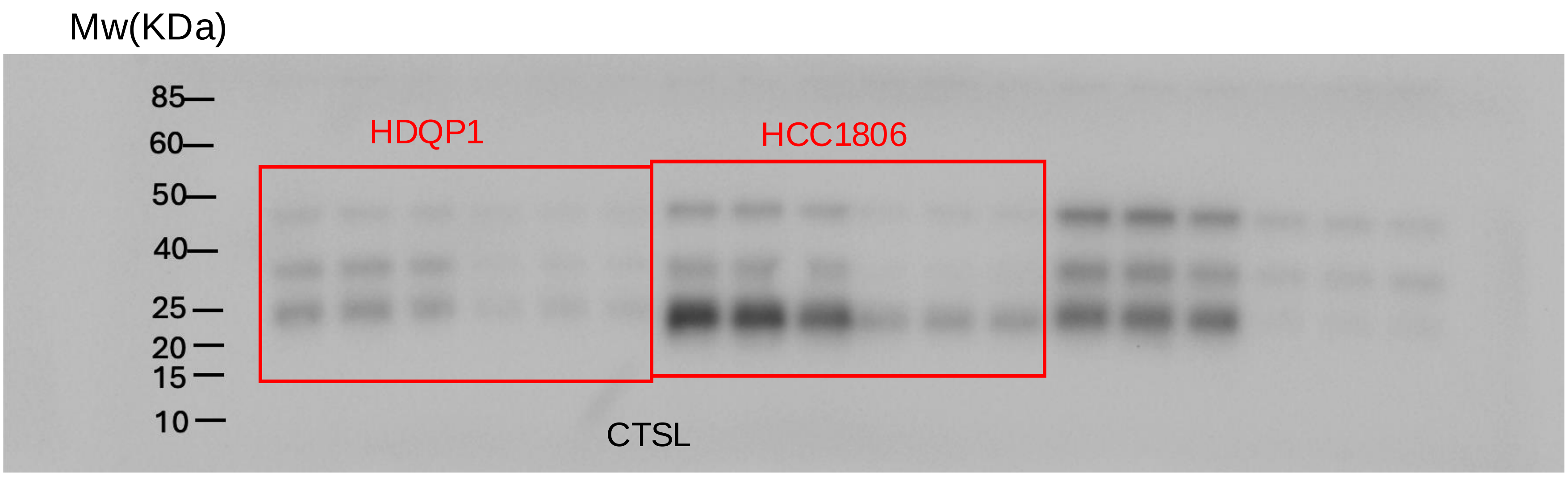

Extended Data Fig. 6 Uncropped blots

Extended Data Fig. 6i

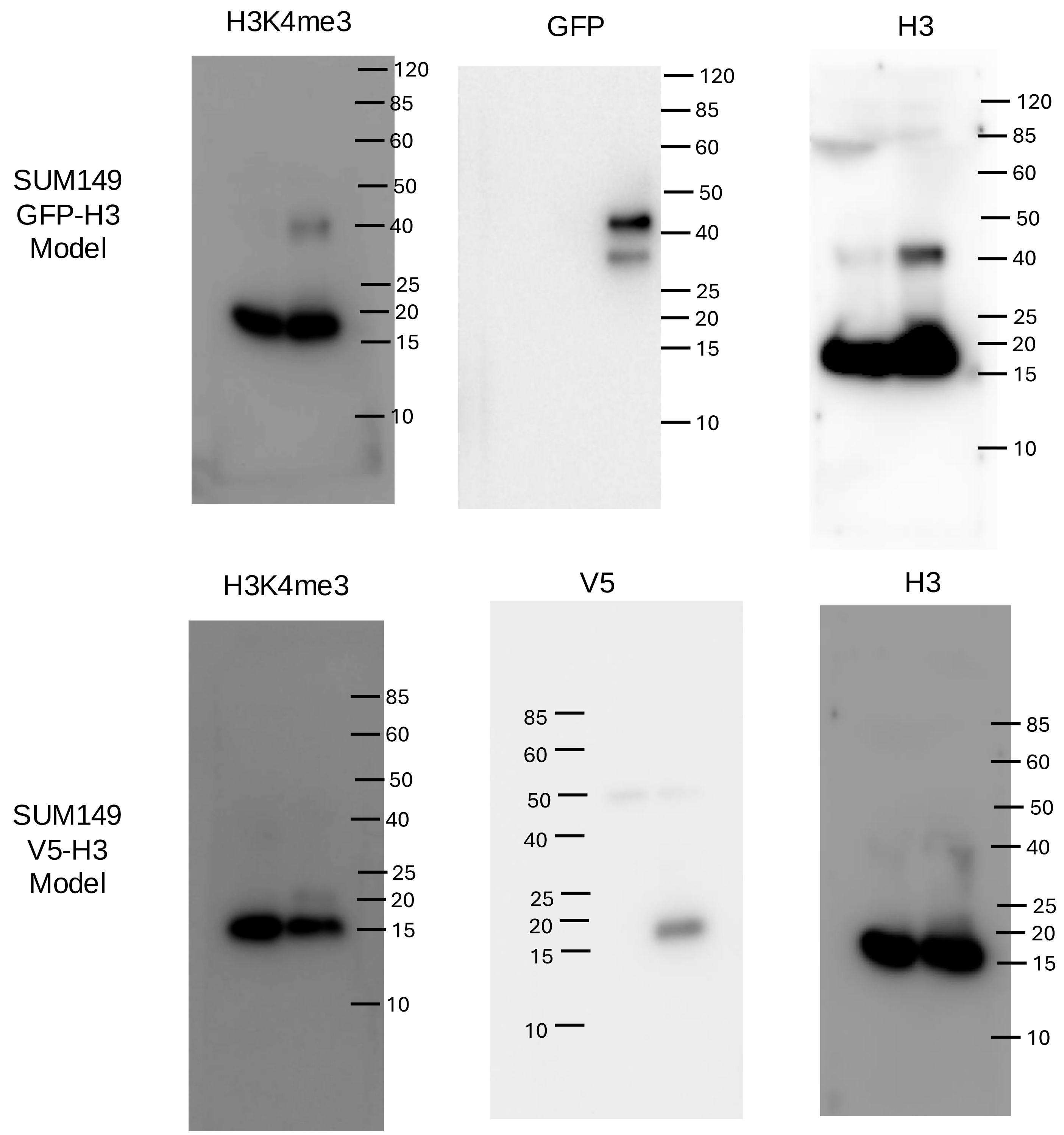

Supplement: Supplementary file 30 — Unprocessed western blots. [file 41588_2025_2197_MOESM30_ESM.pdf]
