# Supplementary material for: KDM4C inhibition blocks tumor growth in basal breast cancer by promoting cathepsin L-mediated histone H3 cleavage
Source: Nat Genet. 2025 Jun 2;57(6):1463–77. doi: 10.1038/s41588-025-02197-z (PMC12165855; doi:10.1038/s41588-025-02197-z)
Supplement: Supplementary file 32 — Unprocessed western blots. [file 41588_2025_2197_MOESM32_ESM.pdf]

Extended Data Fig. 9 Uncropped blots

Extended Data Fig. 9c

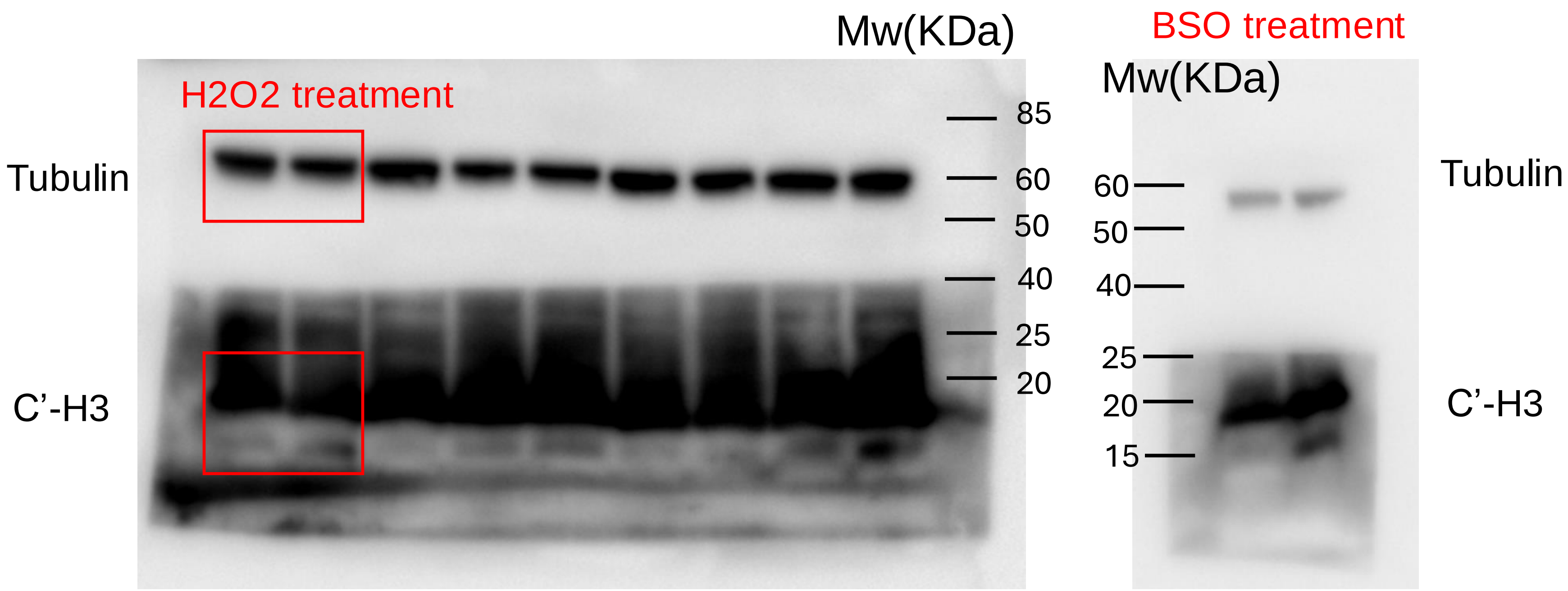

Extended Data Fig. 9l

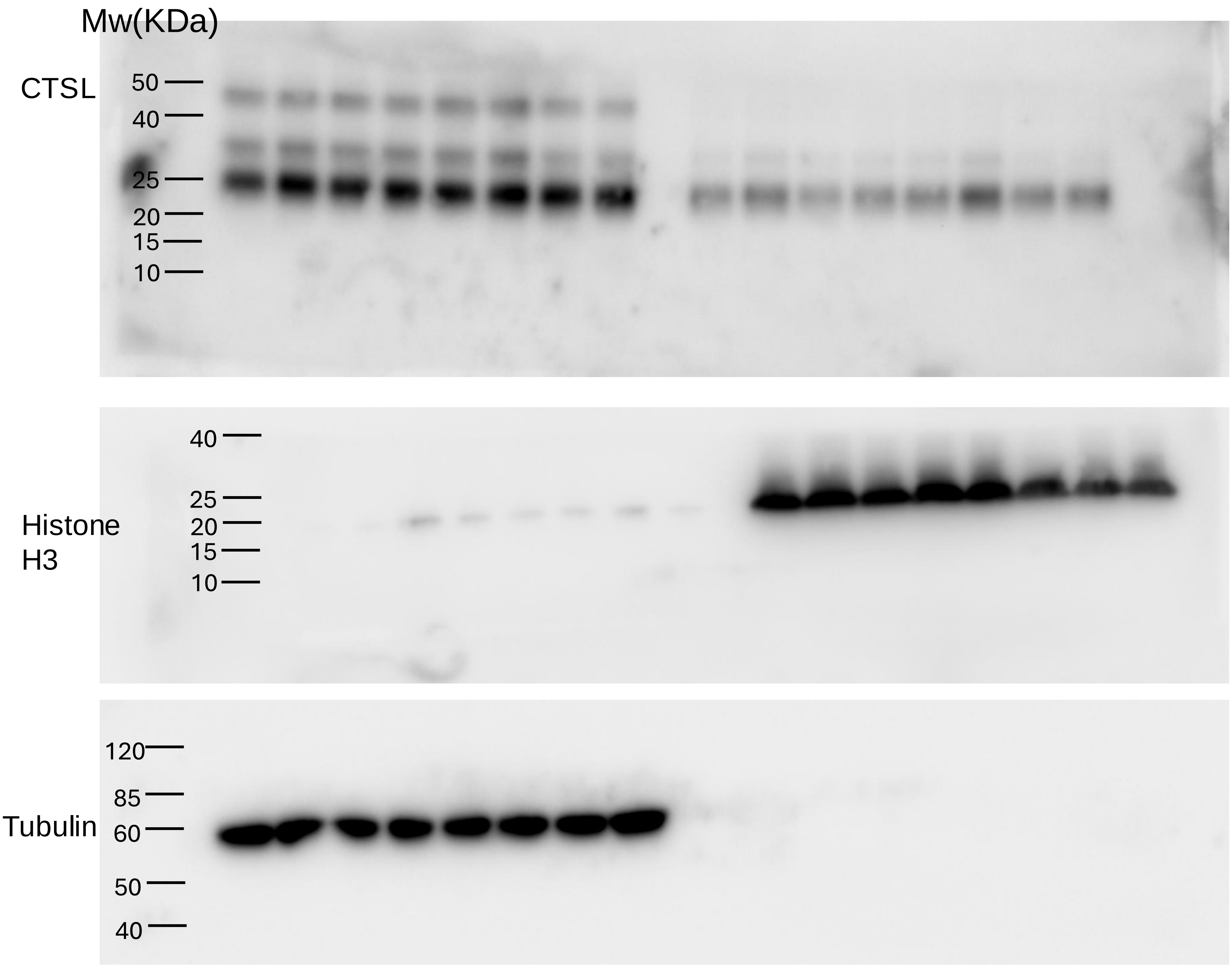

Cell fraction markers were run on separate gels using the same lysates.
